# Catalytic, *Z*-Selective, Semi-Hydrogenation of Alkynes with a Zinc–Anilide Complex

**DOI:** 10.1021/jacs.3c02301

**Published:** 2023-03-27

**Authors:** Greg J. Baker, Andrew J. P. White, Ian J. Casely, Damian Grainger, Mark R. Crimmin

**Affiliations:** † Department of Chemistry, Molecular Sciences Research Hub, Imperial College London, 82 Wood Lane, White City, London W12 0BZ, United Kingdom; ‡ Johnson Matthey Technology Centre, Blounts Court, Sonning Common, Reading RG4 9NH, United Kingdom; § Johnson Matthey, 28 Cambridge Science Park, Milton Road, Cambridge CB4 0FP, United Kingdom

## Abstract

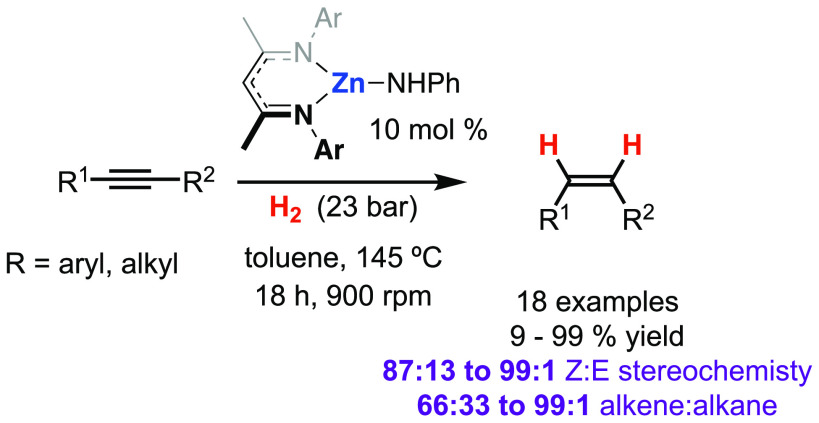

The reversible activation of dihydrogen with a molecular zinc anilide complex is reported. The mechanism of this reaction has been probed through stoichiometric experiments and density functional theory (DFT) calculations. The combined evidence suggests that H_2_ activation occurs by addition across the Zn–N bond *via* a four-membered transition state in which the Zn and N atoms play a dual role of Lewis acid and Lewis base. The zinc hydride complex that results from H_2_ addition has been shown to be remarkably effective for the hydrozincation of C=C bonds at modest temperatures. The scope of hydrozincation includes alkynes, alkenes, and a 1,3-butadiyne. For alkynes, the hydrozincation step is stereospecific leading exclusively to the syn-isomer. Competition experiments show that the hydrozincation of alkynes is faster than the equivalent alkene substrates. These new discoveries have been used to develop a catalytic system for the semi-hydrogenation of alkynes. The catalytic scope includes both aryl- and alkyl-substituted internal alkynes and proceeds with high alkene: alkane, *Z*:*E* ratios, and modest functional group tolerance. This work offers a first example of selective hydrogenation catalysis using zinc complexes.

## Introduction

The activation of dihydrogen is a phenomenon that, until recent years, was associated primarily with transition metals.^1,2^ Significant developments have seen main group complexes reported for the activation of dihydrogen^3−6^ and applications of these systems in catalytic hydrogenation.^7−10^ The activation of dihydrogen using zinc compounds has received little attention. Zinc, as a post-transition metal, does not employ its d-electrons, and so its reactivity is likely to mimic that of a main group center. Dihydrogen activation at zinc is an unusual observation. Although there have been previous reports of the use of zinc-based catalysts for hydrogenation, the fundamental understanding of this process has received limited attention. For example, Beller and co-workers reported the use of zinc triflate, Zn(OTf)_2_, as a pre-catalyst for the hydrogenation of imines.^11^ Stephan and co-workers also used a zinc-based catalyst for the hydrogenation of imines and ketones.^12,13^ Milstein and co-workers provided an example of zinc-catalyzed hydrogenation of imines and ketones, using a PNP pincer ligand to facilitate dihydrogen activation through metal-ligand cooperation.^14^ Very recently, Lacy and co-workers have reported a well-defined Zn catalyst for the hydrogenation of benzophenone and N-benzyl-1-phenylmethanimine.^15^ Despite these reports, the application of zinc to hydrogenation catalysis remains understudied, with substrates limited to highly polarized species. Furthermore, the issue of selective hydrogenation with these types of catalysts is yet to be addressed.

The semi-hydrogenation of alkynes to selectively form alkenes is an important transformation in the synthesis of vitamins and other natural products,^16,17^ as well as being industrially relevant in fields such as polymerization catalysis. The current industrial standard with regard to this process is the palladium-based heterogeneous Lindlar catalyst;^18^ however, in recent years, many homogeneous transition metal systems have also been developed.^19−33^ Due to the current imperative to replace expensive and toxic transition metal catalyst systems, such as those based on palladium, rhodium, ruthenium, or iridium, we were interested in the application of nontransition metal elements to this reaction.

Frustrated Lewis pair (FLP) systems, featuring boranes in combination with a Lewis basic moiety, are some of the most successful homogeneous nontransition metals capable of the selective semi-hydrogenation of internal alkynes.^34−40^ During the revision of this work, Milstein and co-workers reported a Mg-based catalyst for alkyne semi-hydrogenation.^41^ Herein, we report the activation of H_2_ by the 1,2-addition across a Zn–N bond of a novel zinc anilide complex. Further, we show that this key step can be exploited in a highly chemo- and stereoselective semi-hydrogenation of alkynes to form *Z*-alkenes. The approach is inspired by pioneering studies that showed copper hydride reagents can be generated by the 1,2-addition of H_2_ across a Cu–O bond.^42^

## Results

### Synthesis of Zinc Anilides

**Scheme 1:**
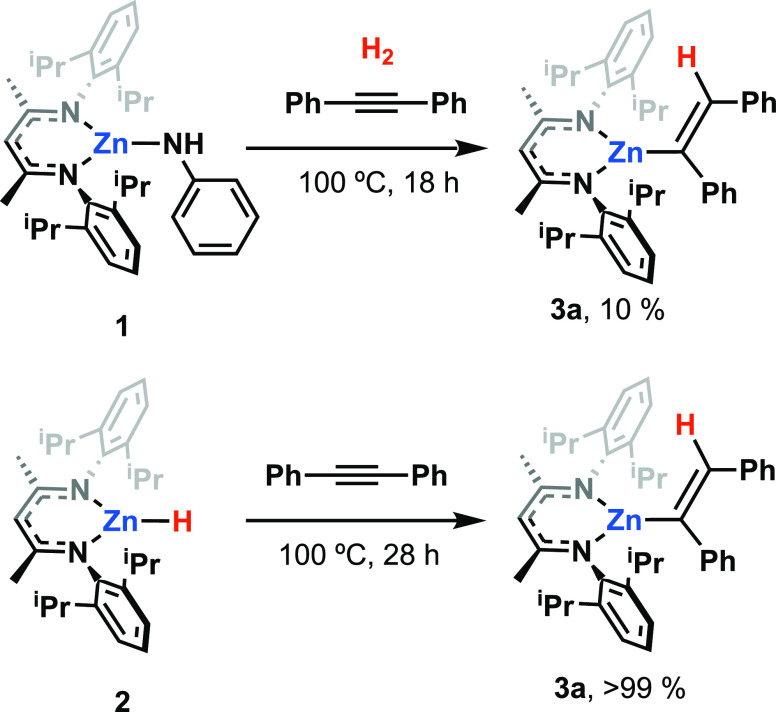
Stoichiometric Reactions of **1** with H_2_ and Diphenylacetylene ^1^H NMR yields.

The zinc anilide complex **1** was synthesized in one-pot by the stepwise reaction of ZnCl_2_, with the lithiated ligand and LiNHPh. The family of terminal, primary zinc–anilide complexes has only a couple of structurally characterized examples,^43,44^ and the reactivity of these species has not been investigated in any detail. **1** was proposed to react with H_2_. An NMR scale reaction of **1** with H_2_ (1 bar) in C_6_D_6_ at 100 °C however yielded only <1% of aniline and the known zinc hydride **2**.^45^ The equilibrium is displaced almost entirely toward the starting materials. Repeating this experiment in the presence of a suitable trapping agent for **2** led to unambiguous evidence of dihydrogen activation at zinc. Hence, the reaction of **1**, H_2_, and diphenylacetylene after 18 h at 100 °C formed the zinc vinyl complex **3a** in a low but non-negligible yield of ∼10%, again the mass balance is unreacted starting materials (Scheme 1).

In separate experiments, **2** reacted cleanly with diphenylacetylene at 100 °C over 28 h to form **3a** in near quantitative yield. These results demonstrate slow dihydrogen activation by **1** and the hydrozincation of an alkyne with the resultant zinc hydride **2**.

### Dihydrogen Activation

**Figure 1:**
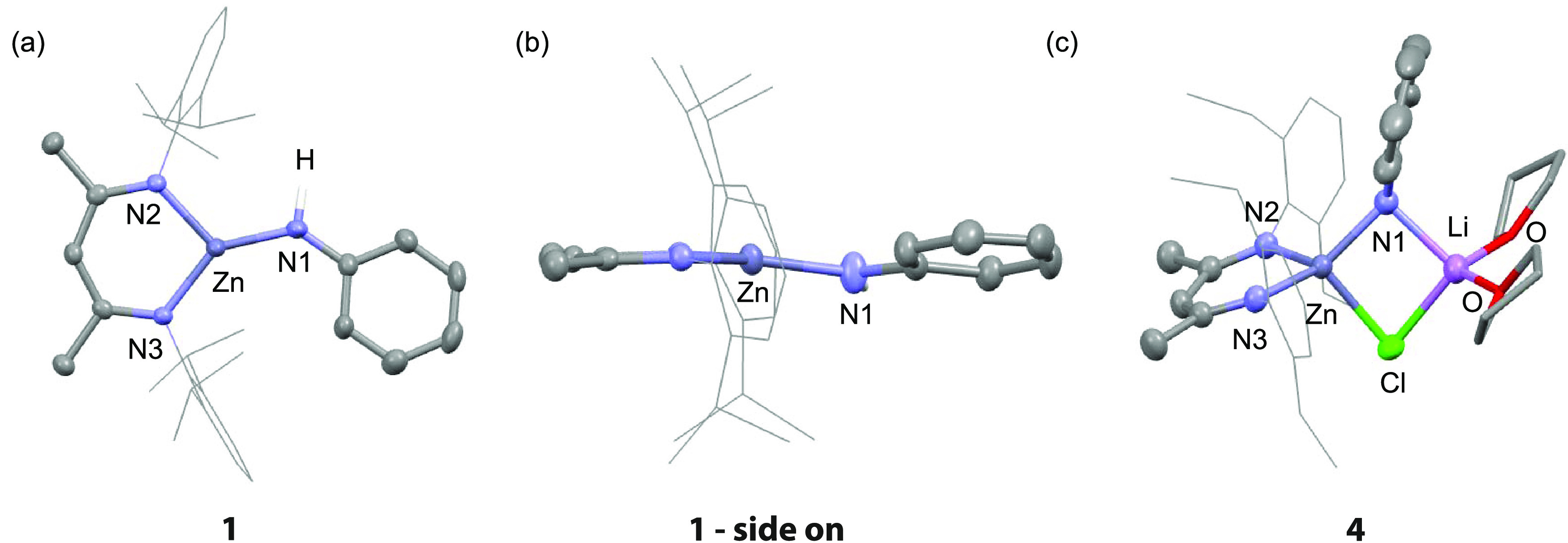
Crystal structure **1** (a) end-on and (b) side-on, along with (c) **4** (50% probability ellipsoids, selected hydrogen atoms omitted for clarity). Only one molecule (1A) of the two molecules in the unit cell of **1** is shown. Hydrogen atoms omitted for clarity. Selected bond distances (Å) **1**: Zn–N1 1.855(2), Zn–N2 1.9140(19), Zn–N3 1.9424(18), N1–Zn–N2 120.38(8), N2–Zn–N3 99.37(8), N1–Zn–N3 139.82(8). **4**: Zn–N1 1.980(5), Zn–N2 1.995(3), Zn–N3 1.995(3) Zn–Cl, 2.3873(12).

**Figure 2:**
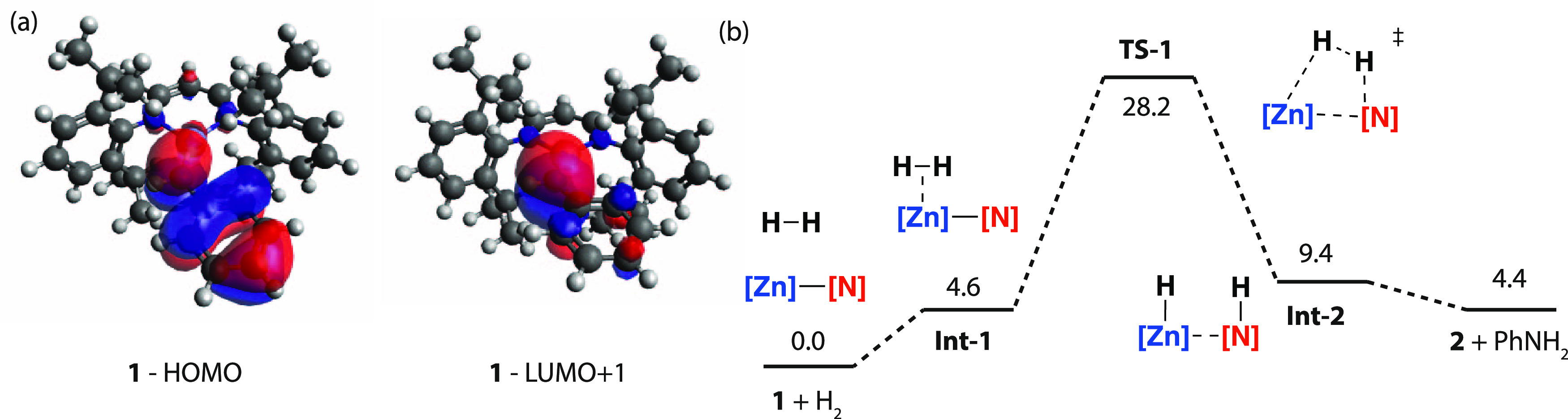
(a) Calculated NLMOs showing the key donor and acceptor orbitals of **1**. (b) Calculated Gibbs Free Energy profile for H_2_ activation using **1**, at standard conditions using the M06L functional and def2TZVPP basis-set. Energies in kcal mol^–1^.

To explore the energetic feasibility of the postulated dihydrogen activation process, we performed density functional theory (DFT) calculations using the M06L functional and a def2TZVPP basis-set (see Supporting Information for details). In the solid state, **1** possesses an arrangement in which the core of the β-diketiminate ligand, the Zn–N motif, and the phenyl ring of the anilide all lie within the same plane (Figure 1). This conformation is reproduced by DFT calculations and results in an electronic structure in which the frontier molecular orbitals of **1** are perfectly set up to react with dihydrogen. Hence, the natural localized molecular orbitals (NLMOs) of **1** allow the visualization of the 2p-orbital on N and the 4s-orbital on Zn, which contribute to the HOMO and LUMO + 1, respectively (Figure 2a). The dual Lewis acidic/Lewis basic behavior of the Zn–N bond of **1** was further supported by attempts to prepare an analogue of this compound. Variation of the steric demands of the ligand on zinc (Ar = 2,6-diisopropylphenyl vs 2,6-diethylphenyl) led to the isolation of the zincate complex **4**, in which an equiv of Li–Cl is coordinated to the Zn–N bond (Figure 1).

Dihydrogen activation by **1** was calculated to occur through a stepwise process involving the formation of a weakly bound encounter complex **Int-1**, which leads to **TS-1**. **Int-1** is an unstable encounter complex of zinc with dihydrogen. While there is a component of bonding that involves the donation of electron density from the H–H bond to the vacant 4s-orbital of Zn in **Int-1**, it is not significant enough to assign this as a dihydrogen complex of zinc.

Dihydrogen splitting occurs by translation of the H_2_ molecule across the zinc center, toward the anilide nitrogen atom, elongating both the H–H and Zn–N distances as it nears the transition state geometry. In **TS-1**, the population of the σ*-orbital of H_2_ occurs through the donation of electron density from the N-based LP, breaking the H–H bond and forming **Int-2**, a weakly bound adduct of **2** and H_2_NPh. The observations that dihydrogen activation by **1** occurs slowly at 100 °C and that there is a strong preference for the reverse reaction suggest a large energy barrier to H_2_ activation and an endergonic reaction. These expectations were confirmed by the calculated Δ*G*_298K_^°^ and Δ*G*_298K_^‡^ of dihydrogen activation by **1** which at 298 K and 1 bar are +4.4 and +28.2 kcal mol^–1^, respectively (Figure 2).

### Hydrozincation

Direct hydrozincation of unsaturated bonds is well known, with most reports focusing on polar substrates.^46−52^ However, the direct hydrozincation of alkynes has received little attention. Ingleson and co-workers reported that a cationic two-coordinate zinc hydride species was capable of adding across the triple bond of various alkyne substrates.^53^ They also went on to develop a catalytic system for di- and triborylation of terminal alkynes and showed that their cationic, two-coordinate complex outperformed complex **2** in this system.^54^ Direct hydrozincation was also proposed to occur in the zinc-catalyzed hydroboration of alkynes reported by Geetharani and co-workers.^55^ These reports cover terminal and internal alkynes; however, methods for the uncatalyzed hydrozincation of alkenes are rare.

**Figure 3:**
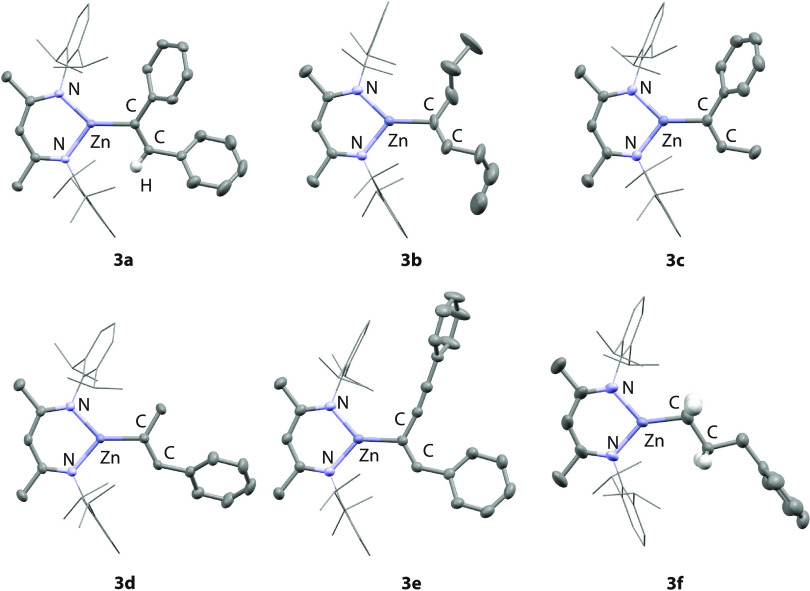
Crystal structures of the products **3a**–**f** (50% probability ellipsoids, selected hydrogen atoms omitted for clarity).

**Scheme 2:**
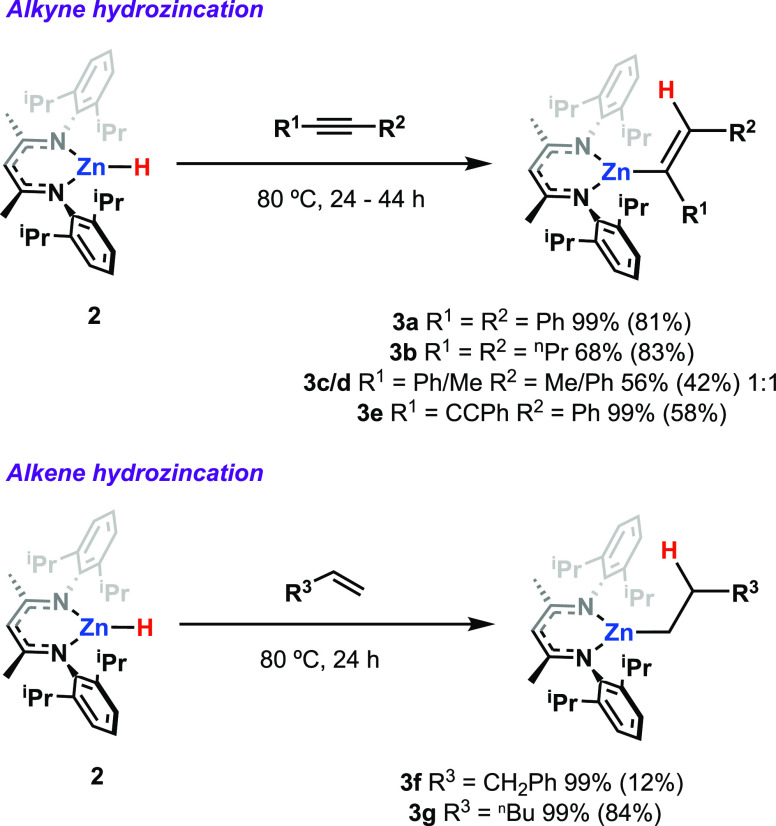
Reactions of **2** with Alkenes and Alkynes to Form Products **3a**–**g**^1^H NMR Yields Shown (Isolated Yields in Parentheses)

The reactivity of intermediate zinc hydride **2** toward a range of unsaturated substrates was investigated. Alkynes readily underwent hydrozincation using **2**, as did terminal alkenes, while internal alkene substrates were generally more sluggish to react. Reaction of **2** with oct-4-yne cleanly forms **3b**. Reaction of **2** with 1-phenyl-1-propyne at 80 °C resulted in the formation of a mixture of two regioisomeric hydrozincation products, **3c** and **3d**, in an approximately 1:1 ratio, 42% yield. The reaction of **2** with 1,4-diphenybutadiyne led to the selective hydrozincation of one of the alkynyl groups to yield **3e**, and in this case, only a single regioisomer was observed in quantitative yield at 80 °C. Reactions of **2** with terminal alkenes 1-allylbenzene and hex-1-ene at 80 °C also formed the corresponding zinc alkyl compounds **3f** and **3g**, respectively (Scheme 2). Based on the consistent stereochemistry of the zinc alkenyl products **3a**–**e** (Figure 3), it is clear the reaction proceeds via a *syn*-addition of Zn–H to the alkyne substrate.^53^ The regiochemistry of hydrozincation is notable. The addition to a phenylprop-1-yne is nonselective, yielding a mixture of Markovnikov and anti-Markovnikov products. 1,4-Diphenylbutadiene proceeds with high selectivity for the Markovnikov product. Terminal alkenes yield anti-Markovnikov products exclusively. These results are predicted based on established trends in hydrometallation chemistry and the expected stabilization of partial charges in the insertion transition state. The hydrozincation step also appeared susceptible to steric effects as internal alkenes react only very slowly with **2**. For example, (*E*)-1,2-diphenylethylene and (*Z*)-1,2-diphenylethylene give only partial conversion (<15%) to the corresponding hydrozincated products after at least 15 h at 100 °C. 1,3-bis(trimethylsilyl)acetylene did not react with **2** under these conditions.

There is the potential for the hydrozincation step to be reversible with regeneration of the starting materials via a β-hydride elimination mechanism. Mixtures of **3c**/**3d** were used to investigate the reversibility. Monitoring the formation **3c**/**3d** as a function of time revealed no change in the 1:1 product ratio with conversion, suggesting that these products do not interconvert at 80 °C. Furthermore, manual separation of crystals of **3c** and **3d** allowed isolation of a sample enriched in **3d** (2.4:1). Re-exposing the enriched mixture to reaction conditions did not result in an equilibration to a 1:1 mixture of **3c:3d**. Similarly, an experiment in which **3g** was heated with allylbenzene to 80 °C did not lead to the formation of the cross-over products. These findings strongly suggest that the hydrozincation step is non-reversible and β-hydride elimination is not in operation for either alkyne or alkene substrates up to temperatures of 80 °C.

An Eyring analysis on the reaction of **2** with diphenylacetylene in C_6_D_6_ under pseudo-first-order conditions (10 equiv) was conducted. Data sets across the 313–353 K temperature range at 10 K intervals were fitted using initial rates and returned activation parameters of Δ*H*^‡^ = 20.6 kcal mol^–1^, Δ*S*^‡^ = −20.5 cal K^–1^ mol^–1^, and Δ*G*_298K_^‡^ = 26.7 kcal mol^–1^. The negative activation entropy is consistent with an intermolecular hydrozincation step, which involves an ordered transition state.

### Catalytic Semi-Hydrogenation of Alkynes

**Figure 4:**
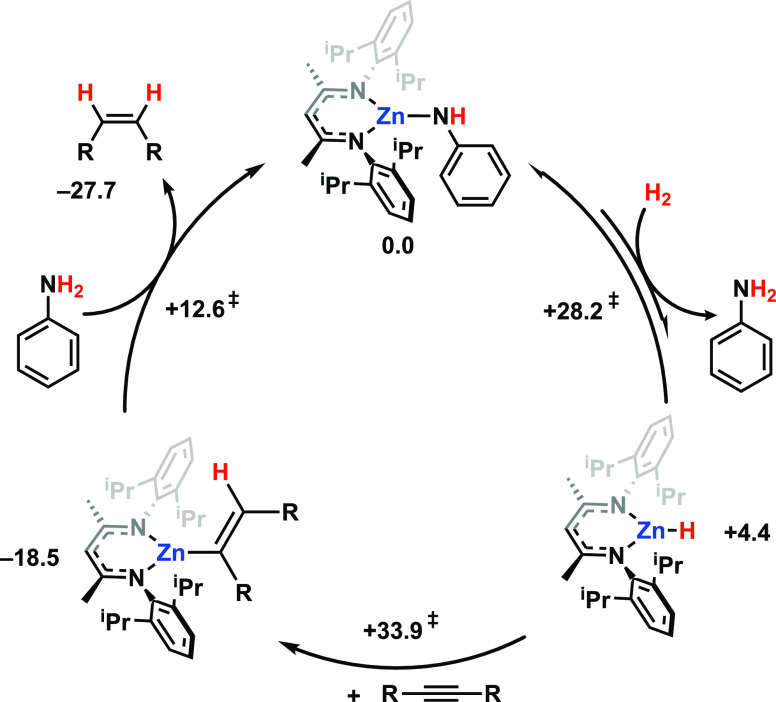
Alkyne semi-hydrogenation catalyzed by **1**. DFT calculated barriers and thermodynamics for R = Ph (kcal mol^–1^).

We postulated that if aniline could protonate the vinyl fragment of **3a**, then the system could be rendered catalytic. The addition of aniline to **3a** at 80 °C led to slow production of **1** and (*Z*)-1,2-diphenylethylene. From these stoichiometric reactions, it is easy to envisage a catalytic cycle very similar to those put forward for copper alkoxide catalysis,^42^ toward (*Z*)-selective alkyne semi-hydrogenation. DFT calculations were used to ascertain that each of the steps involved in catalytic turnover had an accessible Gibbs activation energy (Figure 4). The global energy barrier for turnover is Δ_298_^≠^ = +33.9 kcal mol^–1^, consistent with a reaction that requires forcing conditions. This barrier is associated with two steps, a reversible H_2_ splitting and subsequent diphenylacetylene hydrozincation.

We calculated the local barrier to hydrozincation to be Δ*G*_298K_^‡^ = +29.5 kcal mol^–1^. Similar barriers for the hydrozincation of 1-phenyl-1-propyne with cationic zinc complexes Δ*G*_298K_^‡^ = +28.6 to +31.2 kcal mol^–1^ have been calculated by Ingleson and co-workers.^53^ The calculated thermochemistry is in reasonable agreement with the experimental activation parameters determined for the hydrozincation of diphenylacetylene by **2**, Δ*G*_298K_^‡^ = 26.7 kcal mol^–1^.

**Figure 5:**
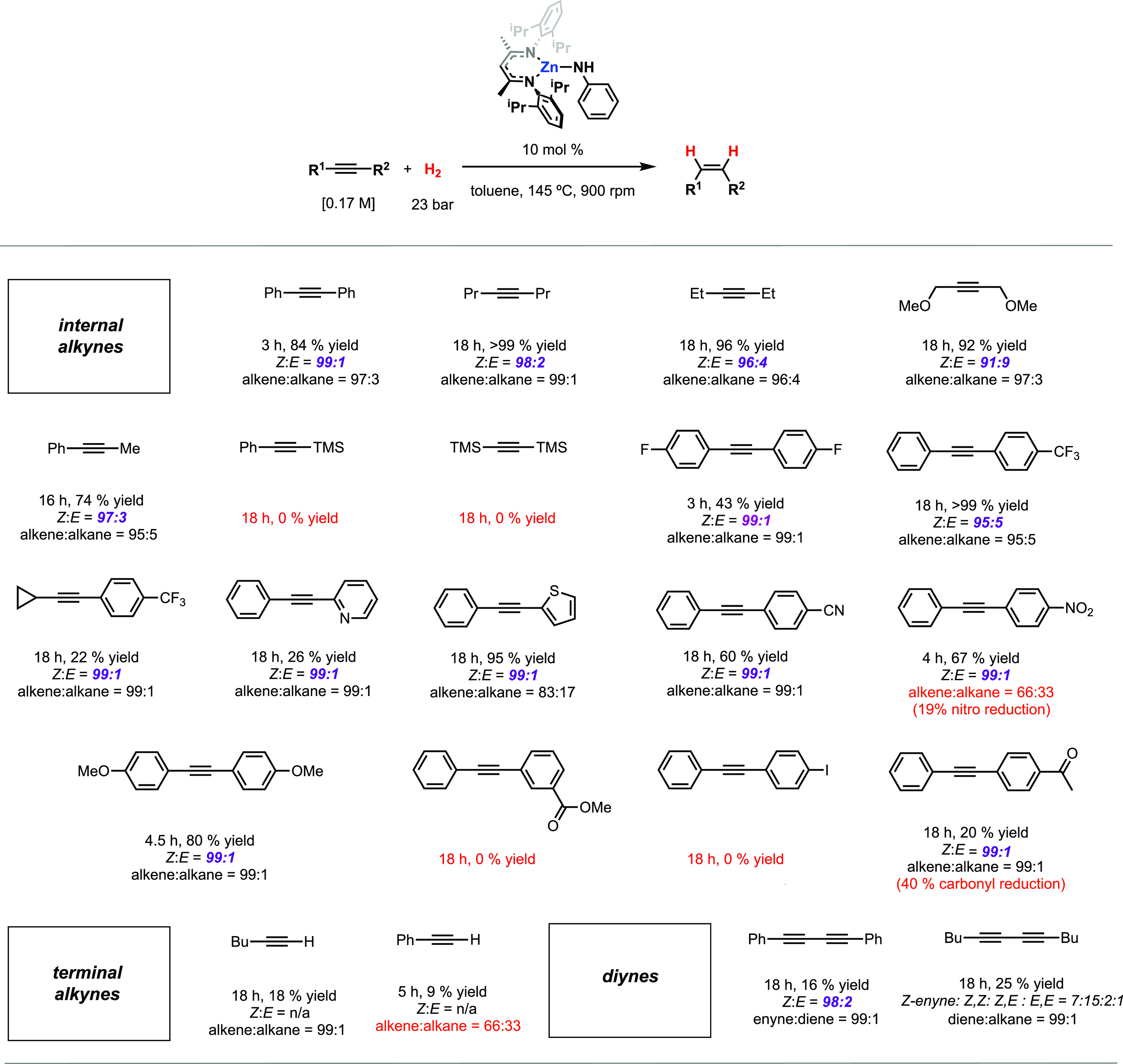
Scope of semi-hydrogenation using **1**.

Hydrogenation reactions were performed at a substrate concentration of 0.17 M in 2–7 mL of solvent. At 145 °C and 23 bar H_2_ pressure, the optimal yield and selectivity for the semi-hydrogenation of diphenylacetylene with 10 mol % **1** were obtained (Figure 5). This reaction produces (*Z*)-1,2-diphenylethylene in >80% with >99:1 stereoselectivity and ∼97:3 chemoselectivity with only small amounts of over hydrogenation to the alkane. Further NMR scale experiments show that **1** is a remarkably robust, albeit slow, catalyst. **1** is stable for 18 h at 178 °C in toluene-d_8_ in a flame-sealed tube. A negative control using no catalyst showed no conversion. 10 mol % **3a** is a poor catalyst leading to only a small amount of product (7%) likely from a stoichiometric reaction. Addition of aniline however could be used to re-establish catalysis. Hence, a mixture of 10 mol % **3a** + 10 mol % PhNH_2_ catalytically converts diphenylacetylene to (*Z*)-1,2-diphenylethylene in >99% with >99:1 stereoselectivity and ∼86:14 chemoselectivity after 4 h at 145 °C and 23 bar H_2_ pressure. **4** was also catalytically active for the semi-hydrogenation of diphenylacetylene to (*Z*)-1,2-diphenylethylene in high yield and selectivity (Table S3).

The proposed mechanism based on DFT calculations predicts that a combination of a reversible hydrogen splitting step and non-reversible alkyne hydrozincation is turnover limiting (pre-equilibrium case). This model predicts that the rate of the reaction should be dependent on both H_2_ pressure and initial alkyne concentration. Turnover frequencies after 18 h at 140 °C (TOF_18h_) were measured for a series of batch reactions run at different H_2_ pressures. TOF_18h_ was found to increase with increasing pressure (5 bar, TOF_18h_ = 0.019 h^–1^; 10 bar, TOF_18h_ = 0.067 h^–1^; 15 bar, TOF_18h_ = 0.411 h^–1^; 20 bar, TOF_18h_ = 0.550 h^–1^). The effect of initial alkyne concentration proved more complex. Turnover frequencies were measured after 3 h at 160 °C (TOF_3h_) for a series of initial alkyne concentrations. These experiments suggest that the rate of reaction increases with increasing [alkyne]_0_ but quickly reaches a plateau and then begins to decrease (see Supporting Information). The data are consistent with catalyst deactivation occurring at higher [alkyne]_0_, due to the alkyne acting as an inhibitor or promoting off-cycle events.

The reaction conditions were tested with 22 alkyne substrates (Figure 5). Diarylethynes were ideal substrates, affording high conversion and high selectivities within relatively short reaction times, with electron-rich and -deficient systems giving similar results. Mixed alkyl/aryl and dialkyl internal alkynes also gave good conversion and selectivities, although the longer reaction times required. Halogen, ether, thienyl, pyridyl, nitrile, trifluoromethyl, and cyclopropyl functional groups were all tolerated. While in some cases yields were low, *Z*:*E* stereoselectivity and alkene:alkane chemoselectivity were very high. The hydrogenation of dimethoxybut-2-ene leads to a useful building block for the potential synthesis of ring systems such as pyridoxine (relevant to vitamin B_6_).^56^

Carbonyl and nitro functional groups were less well tolerated under the reaction conditions. Both ketones and nitro groups underwent competitive reduction with the alkyne, leading to mixtures containing either alkenes/alcohols or alkenes/amines. Nevertheless, in the case of 1-nitro-4-(phenylethynyl)benzene, the *Z*-alkene remains the major product of the reaction. An ester-substituted substrate did not participate in hydrogenation catalysis, suggesting this group may act as an inhibitor for turnover. Bis(trimethylsilyl)ethyne and 1-phenyl-2-trimethylsilylethyne did not undergo hydrogenation, consistent with the observations regarding the hydrozincation of these substrates using **2** (*vide supra*). The hydrogenation of terminal alkynes (hex-1-yne, phenylacetylene) was also investigated in this system, and low conversions were achieved for these substrates (Figure 5 and Table S3). Terminal alkynes can potentially deactivate the catalyst. For example, when **1** was combined with hex-1-yne, facile protonolysis of the anilide ligand of **1** led to the formation of the corresponding catalytically inactive zinc acetylide species.^54^ 1,4-Diphenylbutadiyne could also be selectively hydrogenated to either form the *Z*-enyne product in low yield, while dodeca-5,7-diyne formed a mixture including the *Z*,*Z*-diene as the major product.

For comparison, copper(I) or magnesium(II) semi-hydrogenation catalysts show limited scope for nitrile and nitro functional groups. There are only a handful of examples of sp^2^C–CN substituted substrates that undergo selective hydrogenation with homogeneous copper catalysts, and those that are reported tend not to be diarylalkynes.^57−62^ Similarly, there is only one example of a copper(1) semi-hydrogenation system that reports the reaction with a substrate bearing a sp^2^C–NO_2_ group, and this does not use H_2_ a reductant. Examples that do use H_2_ either lead to no conversion or complete and unselective over-reduction of the sp^2^C–NH_2_ amine.^57,59,61,63−65^ Furthermore, selective diyne reduction with copper catalysis has been reported to form *Z*,*Z*-dienes for diaryl-substituted substrates and *E*,*E*-dienes for dialkyl-substituted substrates.^66,67^ The zinc-base catalyst reported herein, while proceeding with low conversion appears to give access to complementary selectivities.

### Origin of Selectivity

**Scheme 3:**
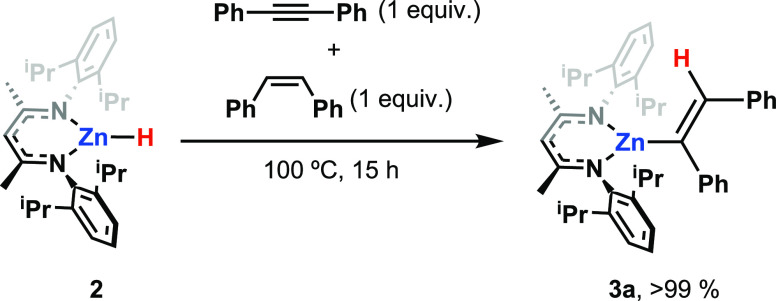
Competition Reactions of **2** with Internal Alkenes and Alkynes

The origin of the remarkable selectivity achieved by **1** in semi-hydrogenation catalysis is likely determined by the reactivity of the intermediate **2** toward unsaturated substrates. The hydrozincation of alkynes with **2** occurs by a stereospecific reaction to form the *syn*-isomer, and this leads exclusively to the *Z*-alkene on protonation, resulting in high *Z*:*E* ratios. The high alkene:alkane ratios can be understood by considering the relative rates of hydrozincation of alkynes and alkenes with **2**. Competition reactions in which a 1:1 mixture of diphenylacetylene: (*Z*)-1,2-diphenylethene (or diphenylacetylene: (*E*)-1,2-diphenylethene) were reacted with **2** at 100 °C for 15 h resulted in exclusive and quantitative formation of **3a**, and no zinc-alkyl species was observed (Scheme 3).

## Conclusions

In summary, a novel system for H_2_ activation using a zinc anilide complex has been demonstrated, where dihydrogen is split across a Zn–N bond to yield a reactive zinc hydride species. This observation has been supported by a detailed mechanistic investigation using a combined experimental and computational approach. Molecular orbital analysis allows us to visualize the synergic donor-acceptor interactions that leads to this activation event. Additionally, we have investigated the scope of direct hydrozincation using a terminal zinc hydride species and probed the selectivity of this process. Through this mechanistic understanding, we were able to rationally design a system capable of semi-hydrogenation of alkynes. This system was shown to be effective for the highly *Z*-selective semi-hydrogenation of a range of alkynes, at moderate temperature and pressure. This system represents a rare post-transition metal alternative for semi-hydrogenation catalysis. This work introduces a novel solution to the challenge of dihydrogen activation and significantly broadens the remit of zinc and post-transition metal catalysis.

## Supporting Information Available

Synthetic procedures, kinetic experiments, NMR spectra of all compounds, crystallographic data, and computational methods (PDF)
