# Supplementary material for: Catalytic, Z-Selective, Semi-Hydrogenation of Alkynes with a Zinc–Anilide Complex
Source: J Am Chem Soc. 2023 Mar 27;145(13):7667–74. doi: 10.1021/jacs.3c02301 (PMC10080692; doi:10.1021/jacs.3c02301)
Supplement: Supplementary file 1 — ja3c02301_si_001.pdf [file ja3c02301_si_001.pdf]

Supporting Information for:

Catalytic, *Z*-Selective, Semi-Hydrogenation of Alkynes with a Zinc–Anilide Complex

Greg J. Baker[†], Andrew J. P. White[†], Ian J. Casely*[,‡], Damian Grainger*[,§], Mark R. Crimmin*[,†]

[†]Department of Chemistry, Molecular Sciences Research Hub, Imperial College London, White City, London, W12 0BZ.

[‡]Johnson Matthey Technology Centre, Blounts Court, Sonning Common, Reading, RG4 9NH.

[§]Johnson Matthey, 28 Cambridge Science Park, Milton Road, Cambridge, CB4 0FP.

Table of Contents

1. General Experimental……S2
2. Synthetic Procedures……S4
   2.1. Syntheses……S4
   2.2. NMR Scale Reactions……S12
   2.3. Eyring Analysis……S19
   2.4. Investigation of Potential Pd Contamination of Substrates……S23
   2.5. Catalytic Procedures……S24
3. X-ray Crystallography……S31
4. Computational Studies……S39
5. XYZ Coordinates……S46
6. NMR Spectra……S56
7. References……S65

1. General Experimental

All manipulations were carried out under standard Schlenk-line and glovebox techniques under an inert atmosphere of argon or dinitrogen. An MBraun Labmaster glovebox was employed operating at <0.1 ppm O_2 and <0.1 ppm H_2O. NMR scale reactions were performed in J. Young tap NMR tubes equipped with internal standard capillaries of ferrocene or *bis*(trimethylsilyl)methane in C_6D_6 and were prepared in a glovebox. Solvents were dried over activated alumina from an SPS (solvent purification system) based upon the Grubbs design and degassed before use. Glassware was dried for 12 hours at 120 °C prior to use. Benzene-d_6 and toluene-d_8 were freeze-pump-thaw degassed and stored over 3 Å molecular sieves prior to use. NMR spectra were obtained on BRUKER 400 or 500 MHz machines, all peaks are referenced against residual solvent peak (C_6D_5H δ 7.16 ppm, $C_6D_5CD_2H$ δ 2.09 ppm) with values quoted in ppm. Data were processed in MestReNova. Crystallographic data was collected using Agilent Xcalibur PX Ultra A or Agilent Xcalibur 3E diffractometers, and the structures were refined using the SHELXTL[1] and SHELX-2013[2] program systems.

The β-diketiminate proligands (^{Dipp}BDI)H[3] and (^{Dep}BDI)H[4], complex **2**[5] and 1,2-bis(4-fluorophenyl)ethylene and 1,2-bis(4-methoxyphenyl)ethylene substrates[6] were prepared according to literature procedures. Solvents were freeze-pump-thaw degassed and stored over 3 Å molecular sieves prior to use. Chemicals purchased from Sigma Aldrich, Alfa Aesar, Honeywell or Fluorochem and used without further purification unless stated. Where liquids at 25 °C, reagents were dried over 3 Å sieves and freeze-pump-thaw degassed prior to use. All gases were supplied by BOC and used without further purification or drying. and 1,2-bis(4-methoxyphenol)ethylene[7] were synthesised according to the literature report by Grieco and co-workers.

Quantitative gas chromatography (GC) analyses were performed with an Agilent Technologies 7820A GC with FID detector using either an Agilent DB-WAX or HP-PLOT Q column. The carrier gas used was helium. Durene was used as internal standard.

High pressure (>10 bar) reactions were performed in a Parr Instrument Company Series 4790 GP 25 mL reactor (ID = 1.0”, internal depth = 2.0”) with a 4848-reactor controller. This reactor was used as new, was used solely for this project, and not exposed to transition metal compounds during the duration of the project. Heating was supplied by a hot plate stirrer and metal bead bath or aluminium heating block and monitored using an internal thermocouple. Stirring was provided using a dedicated PTFE coated magnetic stirrer (0.6” x 0.2” x 0.2”) and the reactor was fitted with PTFE liners, both were used solely for this project and not exposed to transition metal salts. All PTFE liners and stirrers were subject to a rigorous cleaning procedure after each reaction. This procedure was: an acetone rinse, then an aqua regia or concentrated HCl soak (10 – 20 minutes) followed by a thorough wash with distilled water and a final rinse with acetone. This cleaning procedure was coupled with appropriate control reactions to minimise any background catalysis.

2. Synthetic Procedures

2.1 Synthesis of Zn Compounds 1-4

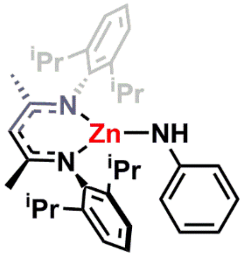

Synthesis of 1: To a clean, dry schlenk under argon, (^{Dipp}BDI)H (5 g, 11.9 mmol) was added and dissolved in THF (20 mL). ^{n}BuLi (1.6 M in hexanes, 7.5 mL, 11.9 mmol) was added dropwise at -78 °C, the reaction was then allowed to warm to 25 °C and stirred for 16 h. In a separate schlenk, anhydrous $ZnCl_2$ (1.6 g, 11.9 mmol) was dissolved in THF (10 mL). The solution of (^{Dipp}BDI)Li was transferred dropwise via cannula onto the solution of $ZnCl_2$ at -78 °C. This mixture was allowed to warm to 25 °C and stirred for 16 h. In a separate schlenk, ^{n}BuLi (1.6 M in n-hexane, 7.5 mL, 11.9 mmol) was added dropwise at -78 °C to a stirred solution of aniline (1.1 mL, 11.9 mmol) in THF (15 mL), this solution was allowed to warm to 25 °C and stirred for 16 h. The solution of LiNHPh was transferred dropwise via cannula onto the solution of (^{Dipp}BDI)ZnCl at 25 °C and stirred for 16 h. Recrystallisation from toluene, washing with n-hexane (2 x 5 mL) and drying under vacuum yielded pure product as an off-white crystalline solid (3.2 g, 5.6 mmol, 42 % yield).

^{1}H NMR (400 MHz, C_6D_6) δ 7.21 – 7.18 (m, 2H, *p*-Dipp), 7.14 – 7.12 (m, 4H, *m*-Dipp), 6.80 (dd, $^{3}J_{HH}$ = 8.5, 7.2 Hz, 2H, Zn-N(*m*-Ph)), 6.47 (tt, $^{3}J_{HH}$ = 7.2 Hz, $^{4}J_{HH}$ = 1.1 Hz, 1H, Zn-N(*p*-Ph)), 5.59 (d, $^{3}J_{HH}$ = 7.6 Hz, 2H, Zn-N(*o*-Ph)), 4.95 (s, 1H, backbone C*H*), 3.15 (hept, $^{3}J_{HH}$ = 6.9 Hz, 4H, ^{i}Pr-C*H*), 2.98 (s, 1H, Zn-N(*H*)), 1.67 (s, 6H, backbone CH_3), 1.24 (d, $^{3}J_{HH}$ = 6.9 Hz, 12H, ^{i}Pr-CH_3), 1.14 (d, $^{3}J_{HH}$ = 6.9 Hz, 12H, ^{i}Pr-CH_3).

^{13}C{^{1}H} NMR (100 MHz, C_6D_6) δ 168.9 (backbone *C*CH$_3$), 154.6 (Zn-N(*ipso*-Ph)), 143.2 (*ipso*-Dipp), 141.6 (*o*-Dipp), 128.8 (Zn-N(*m*-Ph)), 126.4 (*p*-Dipp), 124.0 (*m*-Dipp), 115.8 (Zn-N(*o*-Ph)), 113.6 (Zn-N(*p*-Ph)), 94.8 (backbone *C*H), 28.2 (^{i}Pr-*C*H), 23.9 (^{i}Pr-*C*H$_3$), 23.1 (backbone *C*H$_3$), 23.0 (^{i}Pr-*C*H$_3$).

Anal. Calc. (found) for $C_{35}H_{47}N_3Zn$: C 73.09 (72.91) H 8.24 (8.39) N 7.31 (6.91) %.

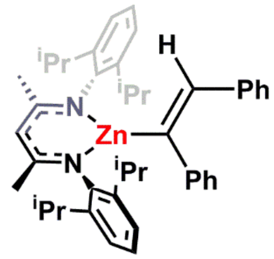

Synthesis of 3a: In a N_2 glovebox, (^{Dipp}BDI)ZnH (100 mg, 0.221 mmol) and diphenylacetylene (37 mg, 0.21 mmol) were dissolved in C_6D_6 and transferred into a J. Young NMR tube. The solution was heated to 100 °C for 28 h in an isothermal bath. A ^{1}H NMR spectrum recorded after the end of the reaction revealed complete conversion of (^{Dipp}BDI)ZnH into the zinc vinyl product and a small amount of the unreacted alkyne. The solvent was removed under reduced pressure and the residue was recrystallised from a minimal amount of n-pentane and a few drops of toluene (5 mL in total) at -35 °C to give **3a** as colourless crystals which were dried *in vacuo* (111 mg, 0.168 mmol, 81 % yield). Crystals suitable for X-ray diffraction were grown from a concentrated *n*-hexane solution at -35 °C.

^{1}H NMR (400 MHz, C_6D_6) δ 7.26 – 7.22 (m, 2H, *p*-Dipp), 7.15 – 7.13 (m, 4H, *m*-Dipp), 6.93 – 6.83 (m, 7H, Zn-C(Ph*H*), Zn-C=C(*m*-Ph)), 6.80 – 6.76 (m, 1H, Zn-C=C(*p*-Ph)), 6.48 (dd, $^3J_{HH}$ = 7.4 Hz $^4J_{HH}$ = 2.0 Hz, 2H, Zn-C=C(*o*-Ph)), 5.77 (s, 1H, Zn-C=C(*H*)), 5.01 (s, 1H, backbone C*H*), 3.17 (hept, $^3J_{HH}$ = 6.9 Hz, 4H, ^{i}Pr-C*H*), 1.71 (s, 6H, backbone C*H*$_3$), 1.18 (d, $^3J_{HH}$ = 6.9 Hz, 12H, ^{i}Pr-C*H*$_3$), 1.14 (d, $^3J_{HH}$ = 6.9 Hz, 12H, ^{i}Pr-C*H*$_3$).

^{13}C{^{1}H} NMR (100 MHz, C_6D_6) δ 167.9 (backbone *C*CH$_3$), 157.5 (Zn-CC(*ipso*-Ph)), 149.0 (Zn-*C*), 145.0 (*ipso*-Dipp), 141.4 (*o*-Dipp), 140.5 (Zn-C=*C*H), 139.1 (Zn-C(*ipso*-Ph)), 129.4 (Zn-C=C(*m*-Ph)), 128.1 (Zn-C(*m*-Ph)), 127.5 (Zn-C(*p*-Ph)), 126.2 (Zn-C=C(*o*-Ph)), 125.9 (*p*-Dipp), 125.6 (Zn-C=C(*p*-Ph)), 123.9 (*m*-Dipp), 123.1 (Zn-C(*o*-Ph)), 95.1 (backbone *C*H), 28.3 (^{i}Pr-*C*H), 23.6 (^{i}Pr-*C*H$_3$), 23.3 (^{i}Pr-*C*H$_3$), 23.0 (backbone C*C*H$_3$).

Anal. Calc. (found) for $C_{43}H_{52}N_2Zn$: C 77.98 (77.95) H 7.91 (7.98) N 4.23 (4.29) %.

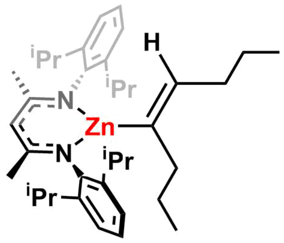

Synthesis of 3b: In a N_2 glovebox, (^{Dipp}BDI)ZnH (204 mg, 0.421 mmol) and 4-octyne (65 µL, 0.443 mmol) were added to a 20 mL J. Young valve ampoule and dissolved in toluene (7 mL). A magnetic stirrer bar was added and the ampoule sealed and heated to 80 °C in a mineral oil bath with stirring for 44 hours. The solvent was removed under reduced pressure to give the product **3b** as a pale green solid (228 g, 0.344 mmol, 83 % yield). Crystals of the product were grown from a concentrated *n*-hexane solution at -35 °C.

^{1}H NMR (400 MHz, C_6D_6) δ 7.18 – 7.11 (m, 6H, *m*-Dipp, *p*-Dipp), 5.02 (s, 1H, backbone C*H*), 4.59 (dd, $^3J_{HH}$ = 7.2, 6.0 Hz, 1H, Zn-CC*H*), 3.21 (hept, $^3J_{\underline{HH}}$ = 6.8 Hz, 4H, ^{i}Pr-C*H*), 1.97 (dt, $^3J_{HH}$ = 7.0 Hz, 2H, Zn-C=CHC*H*$_2$), 1.91 – 1.87 (m, 2H, Zn-CC*H*$_2$), 1.73 (s, 6H, backbone C*H*$_3$), 1.34 (d, $^3J_{HH}$ = 6.9 Hz, 12H, ^{i}Pr-C*H*$_3$), 1.24 – 1.16 (m, 14H, Zn-C=CHCH$_2$C*H*$_2$, iPr-C*H*$_3$), 0.89 – 0.79 (m, 5H, Zn-CCH$_2$C*H*$_2$, Zn-C=CHCH$_2$CH$_2$C*H*$_3$), 0.73 (t, $^3J_{HH}$ = 7.0 Hz, 3H, Zn-CCH$_2$CH$_2$C*H*$_3$).

^{13}C{^{1}H} NMR (100 MHz, C_6D_6) δ 167.3 (backbone *C*CH$_3$), 151.1 (Zn-*C*), 145.3 (*ipso*-Dipp), 142.8 (Zn-C=*C*H), 141.2 (*o*-Dipp), 125.6 (*p*-Dipp), 123.7 (*m*-Dipp), 94.9 (backbone *C*H), 36.9 (Zn-C*C*H$_2$), 30.0 (Zn-C=CH*C*H$_2$), 28.2 (^{i}Pr-*C*H), 23.9 (^{i}Pr-*C*H$_3$), 23.6 (Zn-CCH$_2$*C*H$_2$, Zn-C=CHCH$_2$*C*H$_2$), 23.5 (^{i}Pr-*C*H$_3$), 23.1 (backbone *C*H$_3$), 14.4 (Zn-CCH$_2$CH$_2$*C*H$_3$), 13.9 (Zn-C=CHCH$_2$CH$_2$*C*H$_3$).

Anal. Calc. (found) for $C_{37}H_{56}N_2Zn$: C 74.78 (74.42) H 9.50 (9.56) N 4.71 (4.72) %.

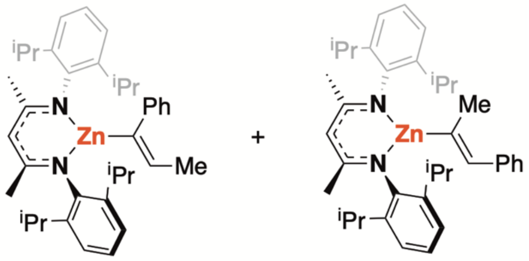

3c **3d**

Synthesis of 3c/3d: In a N_2 glovebox, (^{Dipp}BDI)ZnH (100 mg, 0.21 mmol) and 1-phenyl-1-propyne (26 µL, 0.21 mmol) were added to a 20 mL J. Young valve ampoule and dissolved in toluene (7 mL). A magnetic stirrer bar was added, and the ampoule sealed and heated to 80 °C in a mineral oil bath with stirring for 24 hours. The solvent was removed under reduced pressure and the ampoule was returned to the glovebox. The ratio of **3c : 3d** in the crude mixture was 1:1 as determined by NMR spectroscopy. The crude reaction mixture was dissolved in n-hexane (5 mL). Crystals of the product were grown from a concentrated *n*-hexane solution at -35 °C (52 mg, 0.09 mmol, 42 % yield) as a mixture of **3c : 3d**. The ratio of **3c : 3d** that crystallised varied from sample to sample (between 4:1 and 1.4:1).

^{1}H NMR (400 MHz, C_6D_6) δ 7.07 – 7.77 (m, 8H, Ar*H*, **3c** + **3d**), 6.87 – 6.96 (m, 2H, Ar*H*, **3c** + **3d**), 6.42 – 6.44 (m, 1H, Ar*H*, **3c** + **3d**), 5.79 (q, $^4J_{HH}$ = 2.0 Hz, 1H, Zn-CC*H*, **3d**), 5.04 (q, $^3J_{HH}$ = 7.0 Hz, 1H, Zn-CC*H*, **3c**), 5.02 (s, 1H, backbone C*H,* **3d**), 4.92 (s, 1H, backbone C*H,* **3c**), 3.23 (hept, $^3J_{HH}$ = 6.0 Hz, 4H, ^{i}Pr-C*H*, **3d**), 3.15 (hept, $^3J_{HH}$ = 6.0 Hz, 4H, ^{i}Pr-C*H*, **3c**), 1.73 (s, 6H, backbone C*H*$_3$, **3d**), 1.70 (s, 6H, backbone C*H*$_3$, **3c**), 1.66 (d, $^3J_{HH}$ = 2.0 Hz, 3H, Zn-CC*Me*, **3d**), 1.48 (d, $^3J_{HH}$ = 7.0 Hz, 3H, Zn-CC*Me*, **3c**), 1.31 (d, $^3J_{HH}$ = 6.0 Hz, 12H, iPr-C*H*$_3$, **3d**), 1.19 (d, $^3J_{HH}$ = 6.5 Hz, 12H, iPr-C*H*$_3$, **3c**), 1.17 (d, $^3J_{HH}$ = 6.0 Hz, 12H, iPr-C*H*$_3$, **3d**), 1.13 (d, $^3J_{HH}$ = 6.5 Hz, 12H, iPr-C*H*$_3$, **3c**).

^{13}C{^{1}H} NMR (100 MHz, C_6D_6) δ 168.1 (backbone *C*CH$_3$, **3c**), 167.8 (backbone *C*CH$_3$, **3d**), 154.3 (Zn-*C*, **3c**), 151.7 (Zn-*C*, **3d**), 148.6, 145.3, 145.2, 142.0 (Zn-C*C*H, **3d**), 141.6, 141.6, 140.0, 137.0 (Zn-C*C*H, **3c**), 129.4, 128.4, 127.9, 127.1, 126.3, 126.1, 125.5, 124.2, 124.1, 123.1, 95.4 (backbone *C*H, **3c**), 95.3 (backbone *C*H, **3d**), 28.6 (^{i}Pr-*C*H, **3c**), 28.6 (^{i}Pr-*C*H, **3d**), 24.3 (^{i}Pr-*C*H$_3$, **3d**), 24.0 (^{i}Pr-*C*H$_3$, **3c**), 23.7 (^{i}Pr-*C*H$_3$, **3c**), 23.6 (^{i}Pr-*C*H$_3$, **3d**), 23.4 (backbone *C*H$_3$, **3d**), 23.2 (backbone *C*H$_3$, **3c**), 22.3 (Zn-CC*Me*, **3d**), 16.3 (Zn-CC*Me*, **3c**).

Anal. Calc. (found) for $C_{38}H_{50}N_2Zn$: C 76.04 (75.55) H 8.40 (8.34) N 4.67 (4.53) %.

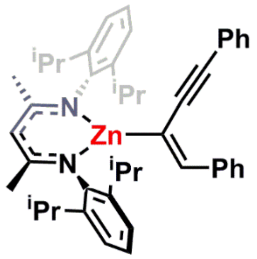

Synthesis of 3e: In a N_2 glovebox, (^{Dipp}BDI)ZnH (100 mg, 0.21 mmol) and 1,4-diphenylbutadiyne (42 mg, 0.21 mmol) were added to a 20 mL J. Young valve ampoule and dissolved in toluene (7 mL). A magnetic stirrer bar was added, and the ampoule sealed and heated to 80 °C in a mineral oil bath with stirring for 24 hours. The solvent was removed under reduced pressure and the ampoule returned to the glovebox. The product was dissolved in n-hexane (5 mL) and filtered through a glass fibre. Crystals of the product **3e** were grown from a concentrated *n*-hexane solution at -35 °C (80 mg, 0.12 mmol, 58 % yield).

^{1}H NMR (400 MHz, C_6D_6) δ 7.89 (d, $^3J_{HH}$ = 8.4 Hz, 2H, *Ar*H), 7.18 – 7.02 (m, 12H, *m*-Dipp, *p*-Dipp, Ar*H*), 7.01 – 6.95 (m, 1H, *Ar*H), 6.94 – 6.91 (m, 1H, *Ar*H), 5.70 (s, 1H, Zn-CC*H*), 5.02, (s, 1H, backbone C*H*), 3.21 (hept, $^3J_{\underline{HH}}$ = 6.8 Hz, 4H, ^{i}Pr-C*H*), 1.68 (s, 6H, backbone CH_3), 1.34 (d, $^3J_{HH}$ = 6.8 Hz, 12H, ^{i}Pr-CH_3), 1.15 (d, $^3J_{HH}$ = 6.8 Hz, 12H, ^{i}Pr-CH_3).

^{13}C{^{1}H} NMR (100 MHz, C_6D_6) δ 168.6 (backbone *C*CH$_3$), 150.0 (Zn-C=*C*H), 144.9 (*ipso*-Dipp), 141.9 (*o*-Dipp), 140.0 (Zn-*C*=CH), 131.5, 130.4, 128.9, 128.2, 127.9, 127.5, 126.7, 126.5, 126.4, 124.6 (*m*-Dipp), 100.3 (Zn-C=C-C≡*C*), 96.5 (Zn-C=C-*C*), 95.7 (backbone *C*H), 28.7 (^{i}Pr-*C*H), 24.5 (^{i}Pr-*C*H$_3$), 23.5 (^{i}Pr-*C*H$_3$), 23.4 (backbone *C*H$_3$).

Anal. Calc. (found) for $C_{45}H_{52}N_2Zn$: C 78.75 (77.58) H 7.64 (7.81) N 4.08 (4.03) %.

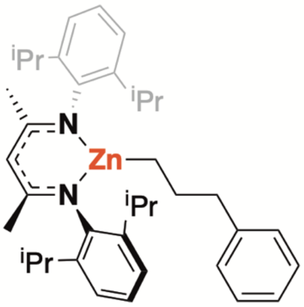

Synthesis of 3f: In a N_2 glovebox, (^{Dipp}BDI)ZnH (100 mg, 0.21 mmol) and allylbenzene (27 µL, 0.21 mmol) were added to a Schlenk tube and dissolved in toluene (7 mL). A magnetic stirrer bar was added. The Schlenk was sealed, removed from the glovebox, and heated to 80 °C with stirring for 24 hours. The solvent was removed under reduced pressure, and the Schlenk returned to the glovebox. The crude product was dissolved in minimum n-pentane (2 mL), filtered, and stored at -35 °C. Crystals of **3f** were grown from this concentrated *n*-pentane solution at -35 °C (15 mg, 0.03 mmol, 12 % yield).

^{1}H NMR (400 MHz, C_6D_6) δ 7.21 – 7.13 (m, 8H, *m*-Dipp, *p*-Dipp, Ar*H*), 7.08 (t, $^3J_{HH}$ = 8.0 Hz, 1H, *p*-Ar*H*), 6.97 (d, $^3J_{HH}$ = 7.2 Hz, 2H, *o*-Ar*H*), 5.04, (s, 1H, backbone C*H*), 3.22 (hept, $^3J_{\underline{HH}}$ = 6.8 Hz, 4H, ^{i}Pr-C*H*), 2.27 – 2.23 (m, 2H, C*H$_2$*Ph), 1.75 (s, 6H, backbone C*H*$_3$), 1.55 – 1.45 (m, 2H, ZnCH$_2$C*H*$_2$-), 1.28 (d, $^3J_{HH}$ = 6.8 Hz, 12H, ^{i}Pr-C*H*$_3$), 1.20 (d, $^3J_{HH}$ = 6.8 Hz, 12H, ^{i}Pr-C*H*$_3$), 0.36 – 0.32 (m, 2H, ZnC*H$_2$*CH$_2$).

^{13}C{^{1}H} NMR (100 MHz, C_6D_6) δ 167.5 (backbone *C*CH$_3$), 145.0 (*ipso*-Dipp), 143.9 (*ipso-Ar*), 141.6 (*o*-Dipp), 128.8 (*o*-Ar), 128.1, 126.0, 125.4 (*p*-Ar), 123.9 (*m*-Dipp), 95.5 (backbone *C*H), 42.7 (*C*H$_2$Ph), 30.1 (Zn*C*H$_2$CH$_2$), 28.6 (^{i}Pr-*C*H), 24.3 (^{i}Pr-*C*H$_3$), 23.6 (^{i}Pr-*C*H$_3$), 23.4 (backbone *C*H$_3$), 7.9 (Zn*C*H$_2$CH$_2$).

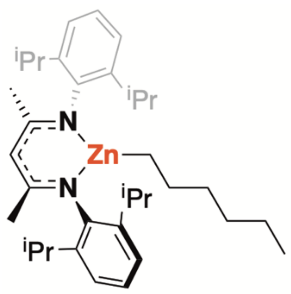

Synthesis of 3g: In a N_2 glovebox, (^{Dipp}BDI)ZnH (100 mg, 0.21 mmol) and hex-1-ene (48 µL, 0.30 mmol) were added to a Schlenk tube and dissolved in toluene (7 mL). A magnetic stirrer bar was added. The Schlenk was sealed, removed from the glovebox, and heated to 80 °C with stirring for 24 hours. The solvent was removed under reduced pressure, and the Schlenk returned to the glovebox. The crude product was dissolved in minimum n-pentane (2 mL), filtered. Crystals of **3g** were grown from slow evaporation of a n-pentane solution (100 mg, 0.18 mmol, 84 % yield).

^{1}H NMR (400 MHz, C_6D_6) δ 7.15 – 7.11 (m, 6H, *m*-Dipp, *p*-Dipp), 5.00, (s, 1H, backbone C*H*), 3.19 (hept, $^{3}J_{HH}$ = 6.8 Hz, 4H, ^{i}Pr-C*H*), 1.70 (s, 6H, backbone CH_3), 1.28 (d, $^{3}J_{HH}$ = 6.8 Hz, 12H, ^{i}Pr-CH_3), 1.22 – 1.06 (m, 6H, CH_2), 1.16 (d, $^{3}J_{HH}$ = 6.8 Hz, 12H, ^{i}Pr-CH_3), 1.00 – 0.90 (m, 2H, CH_2), 0.81 (t, 3H, -CH_2CH_3), 0.29 – 0.25 (m, 2H, $ZnCH_2$).

^{13}C{^{1}H} NMR (100 MHz, C_6D_6) δ 167.4 (backbone *C*CH_3), 145.1 (*ipso*-Dipp), 141.6 (*o*-Dipp), 126.0 (*p*-Dipp), 123.9 (*m*-Dipp), 95.5 (backbone *C*H), 36.3 (CH_2), 32.2 (CH_2), 28.6 (^{i}Pr-*C*H), 28.2 (CH_2), 24.3 (^{i}Pr-CH_3), 24.0 (^{i}Pr-CH_3), 23.5 (backbone CH_3), 23.0 (CH_2), 14.5 (-CH_2CH_3), 8.0 ($ZnCH_2$).

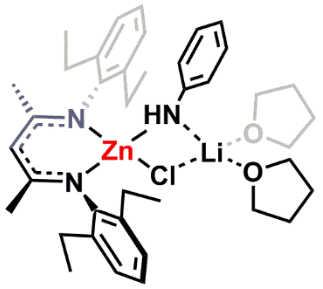

Synthesis of 4: To a clean, dry schlenk under argon, (^{Dep}BDI)H (2 g, 5.5 mmol) was added and dissolved in THF (20 mL). ^{n}BuLi (1.6 M in n-hexane, 3.4 mL, 5.5 mmol) was added dropwise at -78 °C. The reaction was allowed to warm to 25 °C and stirred for 16 h. The solution of (^{Dep}BDI)Li was transferred dropwise via cannula onto a stirred solution of anhydrous $ZnCl_2$ (750 mg, 5.5 mmol) in THF (10 mL) at -78 °C. The reaction was allowed to warm to 25 °C and stirred for 72 h. In a separate schlenk, ^{n}BuLi (1.6 M in n-hexane, 3.4 mL, 5.5 mmol) was added dropwise to a stirred solution of aniline (501 µL, 5.5 mmol) in THF (10 mL) at -78 °C. The reaction was allowed to warm to 25 °C and stirred for 16 h. The solution of LiNHPh was transferred dropwise via cannula onto the solution of (^{Dep}BDI)ZnCl at 25 °C. The reaction was stirred for 16 h. The reaction mixture was concentrated in vacuo and stored at -20 °C to yield large, pale yellow crystals of product (1.9 g, 2.8 mmol, 50 % yield).

^{1}H NMR (400 MHz, toluene-d_8, 353 K) δ 7.07 – 7.01 (br m, 6H, *p*-Dep, *m*-Dep), 6.67 (br t, $^{3}J_{HH}$ = 7.7 Hz, 2H, Zn-N(*m*-Ph)), 6.35 (br t, $^{3}J_{HH}$ = 7.0 Hz, 1H, Zn-N(*p*-Ph)), 5.72 (br d, $^{3}J_{HH}$ = 7.0 Hz, 2H, Zn-N(*o*-Ph)), 4.86 (s, 1H, backbone C*H*), 2.83 (br s, 1H, Zn-N*H*), 2.43 (br q, $^{3}J_{HH}$ = 6.5 Hz, 8H, Et-CH_2), 1.59 (s, 6H, backbone CH_3), 1.11 (t, $^{3}J_{HH}$ = 7.5 Hz, 12H, Et-CH_3).

^{13}C{^{1}H} NMR (100 MHz, toluene-d_8, 353 K) δ 168.6 (backbone *C*CH), 154.7 (Zn-N(*ipso*-Ph)), 146.2 (*ipso*-Dep), 137.6 (*o*-Dep), 129.2 (Zn-N(*m*-Ph)), 126.8 (*m*-Dep), 125.8 (*p*-Dep), 117.8 (Zn-N(*o*-Ph)), 115.4 (Zn-N(*p*-Ph)), 95.1 (backbone *C*H), 24.8 (Et-*C*H_2), 23.2 (backbone *C*H_3), 14.0 (Et-*C*H_3).

^{7}Li NMR (155 MHz, toluene-d_8, 353 K) δ 1.49 (br s).

Anal. Calc. (found) for $C_{39}H_{55}LiN_3O_2ClZn$: C 66.38 (66.46) H 7.86 (7.71) N 5.95 (5.98) %.

2.2 NMR Scale Reactions

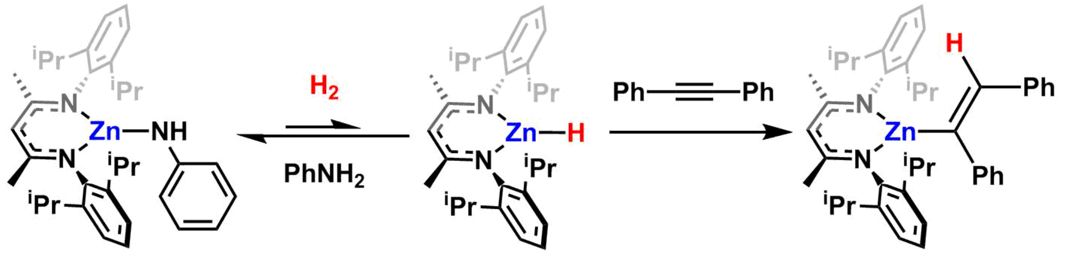

1 **2** **3a**

H_2 activation using 1: In a N_2 glovebox, (^{Dipp}BDI)ZnNHPh **1** (10 mg, 0.017 mmol) and diphenylacetylene (3.1 mg, 0.017 mmol) were added to a weighing vial via C_6D_6 stock solutions. *Bis*(trimethylsilyl)methane (1 µL, 0.005 mmol) was added directly as an internal standard. The reaction volume was made up to 0.6 mL with C_6D_6, before being transferred to a clean, dry J. Young tap NMR tube. The tube contents were freeze-pump-thaw degassed inside the glovebox and the tube was sealed under vacuum. The tube was removed from the glovebox and ~1 atm H_2 added. An initial ^{1}H NMR spectrum was recorded, and then the tube was heated to 100 °C for 18 h. A final ^{1}H NMR spectrum was recorded.

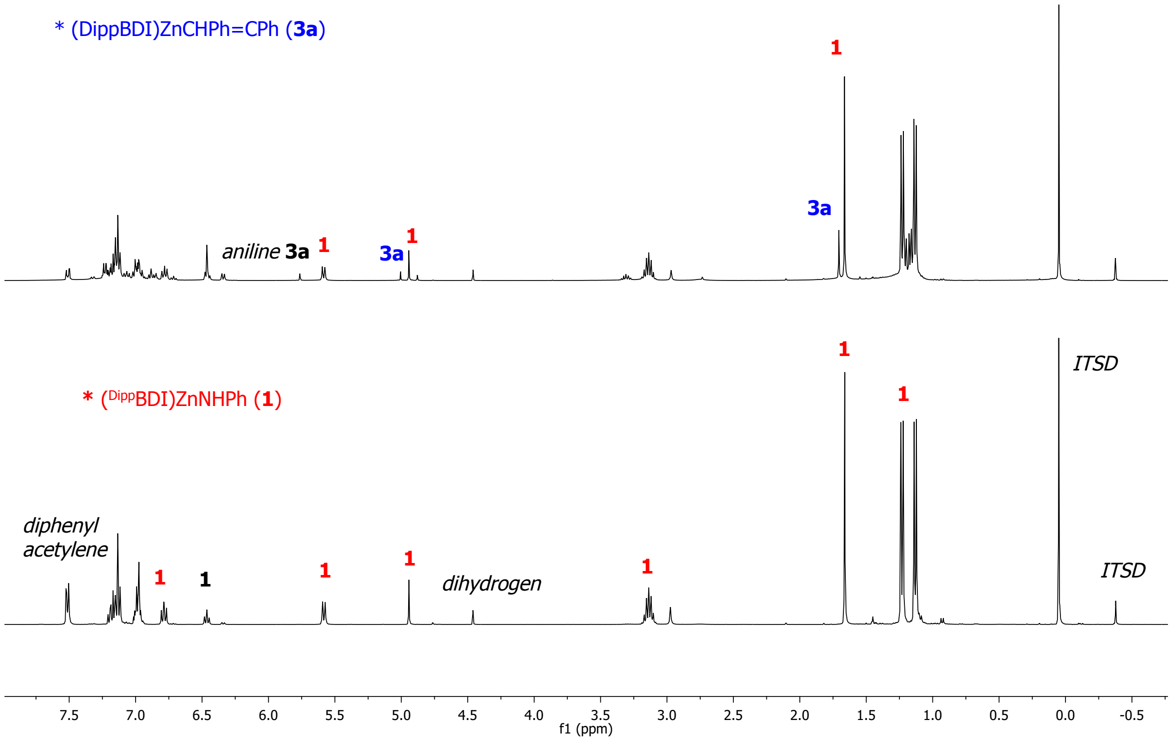

Figure S1. *Initial (bottom) and final (top) ^{1}H NMR spectra of tandem hydrogenation and alkyne insertion of **1**, in C_6D_6.*

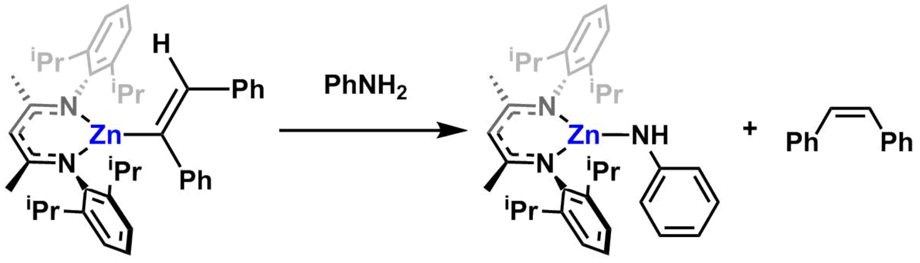

3a **1**

Protonolysis of 3a with aniline: In a N_2 glovebox, (^{Dipp}BDI)ZnC(Ph)CHPh **3** (27.8 mg, 0.042 mmol) and aniline (3.8 µL, 0.042 mmol) were added directly to a clean, dry J. Young NMR tube. *Bis*(trimethylsilyl)methane (1 µL, 0.005 mmol) was added directly as an internal standard. The reaction volume was made up to 0.6 mL with C_6D_6, and the tube was sealed and removed from the glovebox. An initial ^{1}H NMR spectrum was recorded, and then the reaction mixture was heated to 100 °C for 4 days. A final ^{1}H NMR spectrum was recorded.

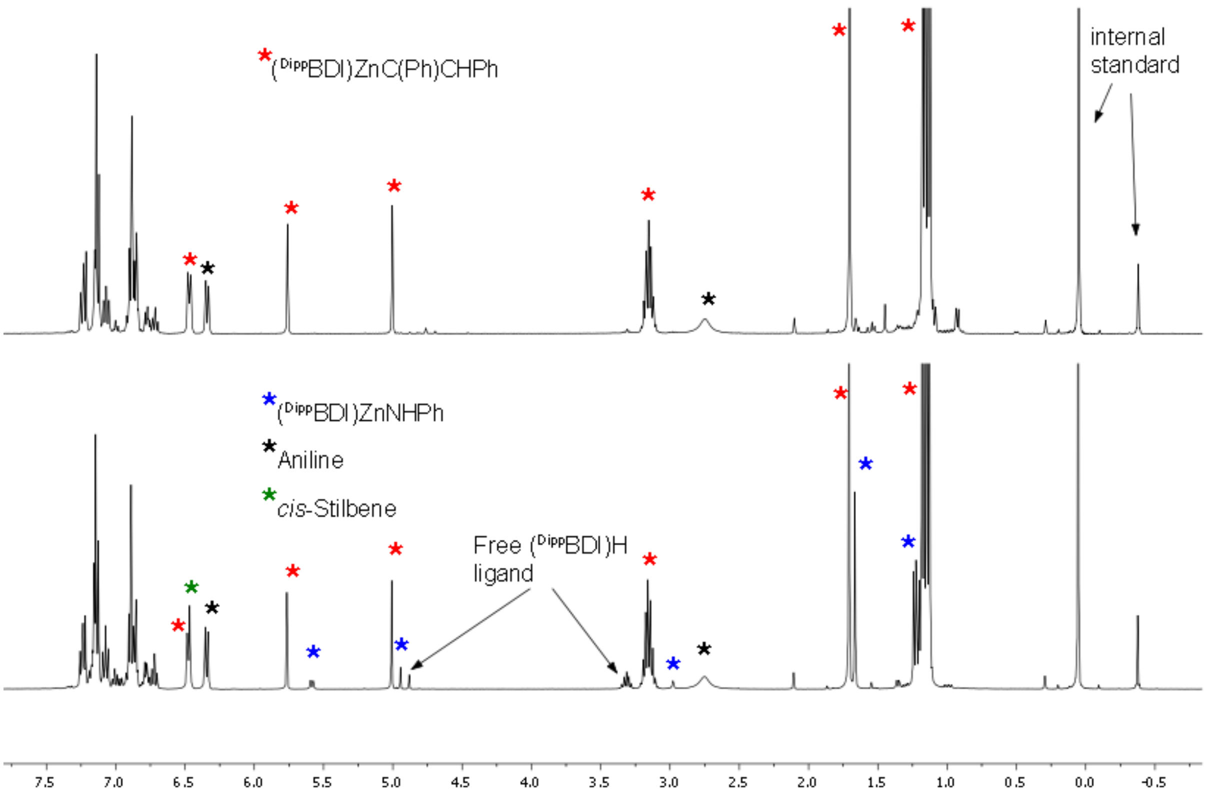

Figure S2. *Initial (top) and final (bottom) ^{1}H NMR spectra of protonolysis reaction of **3a**, in C_6D_6.*

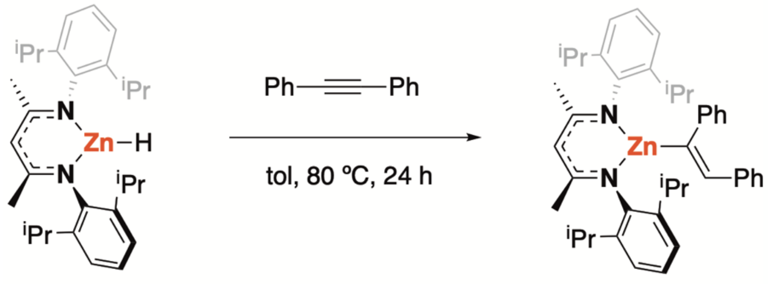

2 **3a**

Hydrozincation of diphenylacetylene with 2: In a N_2 glovebox, (^{Dipp}BDI)ZnH (**2,** 20 mg, 0.042 mmol) and diphenylacetylene (7.4 mg, 0.042 mmol, 1 equiv.) were added directly to a clean, dry J. Young NMR tube. *Ferrocene* (0.005 mmol) was added directly as an internal standard. The reaction volume was made up to 0.6 mL with C_6D_6, and the tube was sealed and removed from the glovebox. An initial ^{1}H NMR spectrum was recorded, and then the reaction mixture was heated to 100 °C for 28 h. A final ^{1}H NMR spectrum was recorded.

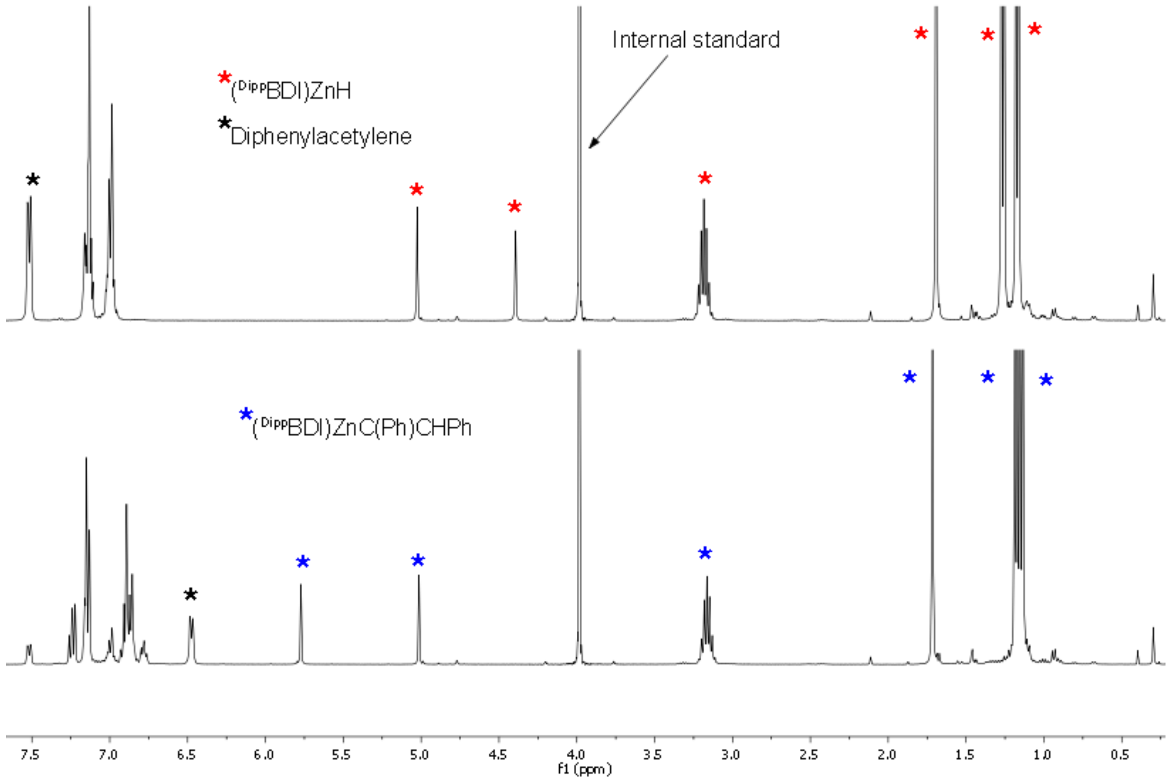

Figure S3. *Initial (top) and final (bottom) ^{1}H NMR spectra of diphenylacetylene hydrozincation using (^{Dipp}BDI)ZnH **2**, in C_6D_6.*

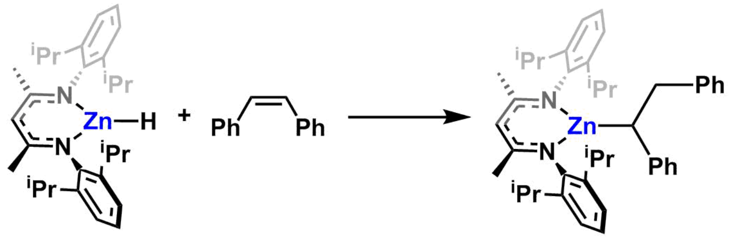

2 *8% NMR yield*

Hydrozincation of Z-1,2-diphenylethene with 2: In a N_2 glovebox, (^{Dipp}BDI)ZnH **2** (7 mg, 0.014 mmol) and *cis*-stilbene (2.6 µL, 0.014 mmol) were added via C_6D_6 stock solutions to a clean, dry J. Young NMR tube. *Bis*(trimethylsilyl)methane (1 µL, 0.005 mmol) was added directly to the tube as an internal standard. The reaction volume was made up to 0.6 mL with C_6D_6, and the tube was sealed and removed from the glovebox. An initial ^{1}H NMR spectrum was recorded, and then the reaction mixture was heated to 100 °C for 19 h. A final ^{1}H NMR spectrum was recorded.

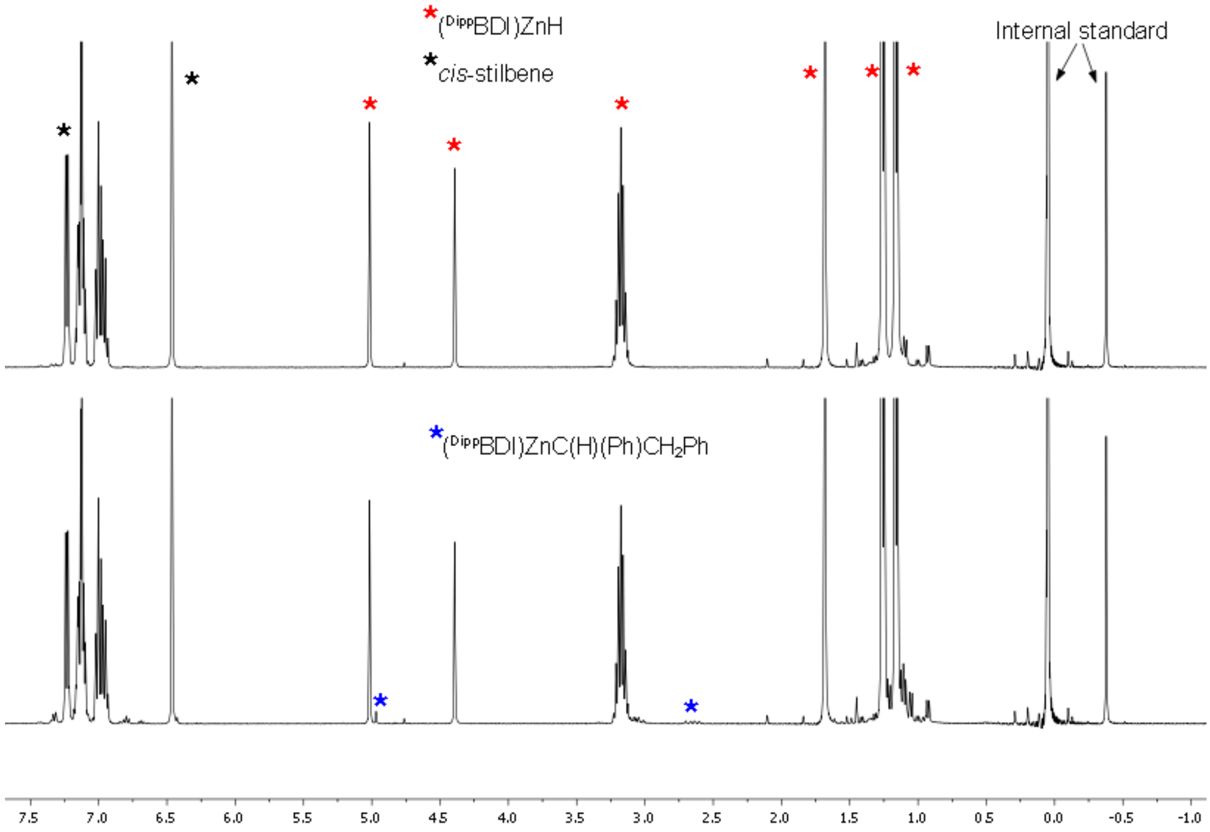

Figure S4. *Initial (top) and final (bottom) ^{1}H NMR spectra of cis-stilbene hydrozincation reaction using (^{Dipp}BDI)ZnH **2**, in C_6D_6.*

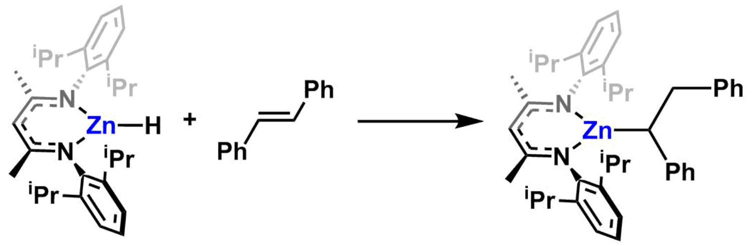

2

14% NMR yield

Hydrozincation of E-1,2-diphenylethene with 2: In a N_2 glovebox, (^{Dipp}BDI)ZnH **2** (7 mg, 0.014 mmol) and *trans*-stilbene (2.6 mg, 0.014 mmol) were added via C_6D_6 stock solutions to a clean, dry J. Young NMR tube. *Bis*(trimethylsilyl)methane (1 µL, 0.005 mmol) was added directly to the tube as an internal standard. The reaction volume was made up to 0.6 mL with C_6D_6, and the tube was sealed and removed from the glovebox. An initial ^{1}H NMR spectrum was recorded, and then the reaction mixture was heated to 100 °C for 15 h. A final ^{1}H NMR spectrum was recorded.

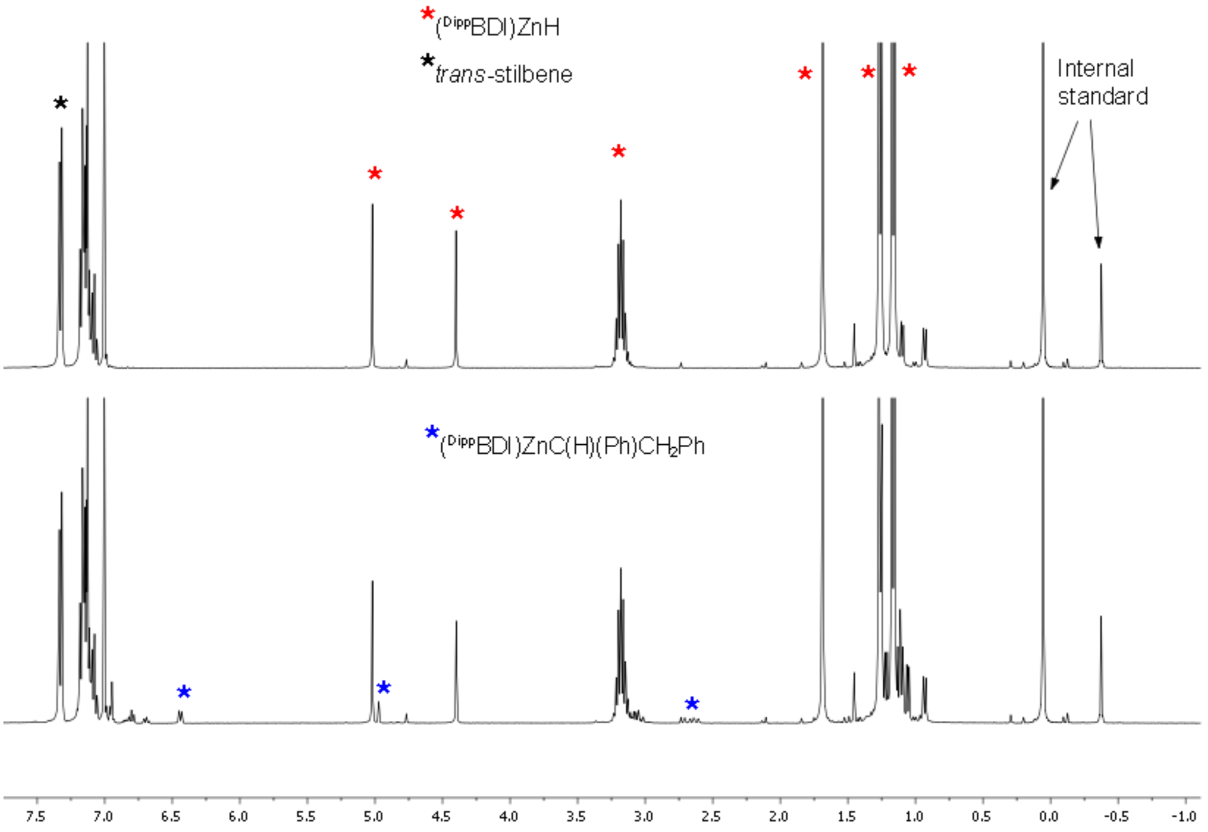

Figure S5. *Initial (top) and final (bottom) ^{1}H NMR spectra of trans-stilbene hydrozincation reaction using (^{Dipp}BDI)ZnH **2**, in C_6D_6.*

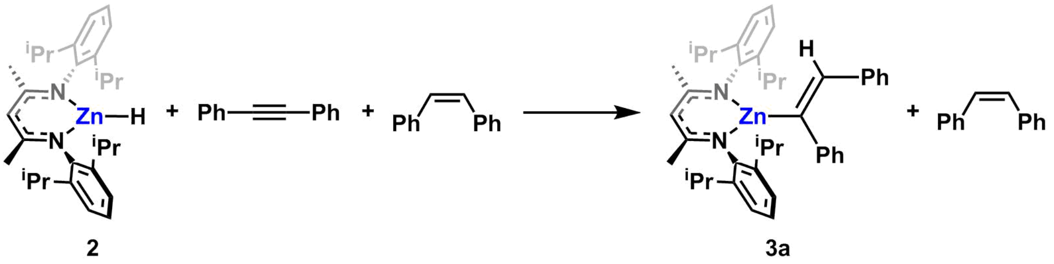

Competitive Alkyne and Alkene Hydrozincation with 2: In a N_2 glovebox, (^{Dipp}BDI)ZnH **2** (7 mg, 0.014 mmol), diphenylacetylene (2.6 mg, 0.014 mmol) and *cis*-stilbene (2.6 µL, 0.014 mmol) were added via C_6D_6 stock solutions to a clean, dry J. Young NMR tube. *Bis*(trimethylsilyl)methane (1 µL, 0.005 mmol) was added directly to the tube as an internal standard. The reaction volume was made up to 0.6 mL with C_6D_6, and the tube was sealed and removed from the glovebox. An initial ^{1}H NMR spectrum was recorded, and then the reaction mixture was heated to 100 °C for 18 h. A final ^{1}H NMR spectrum was recorded.

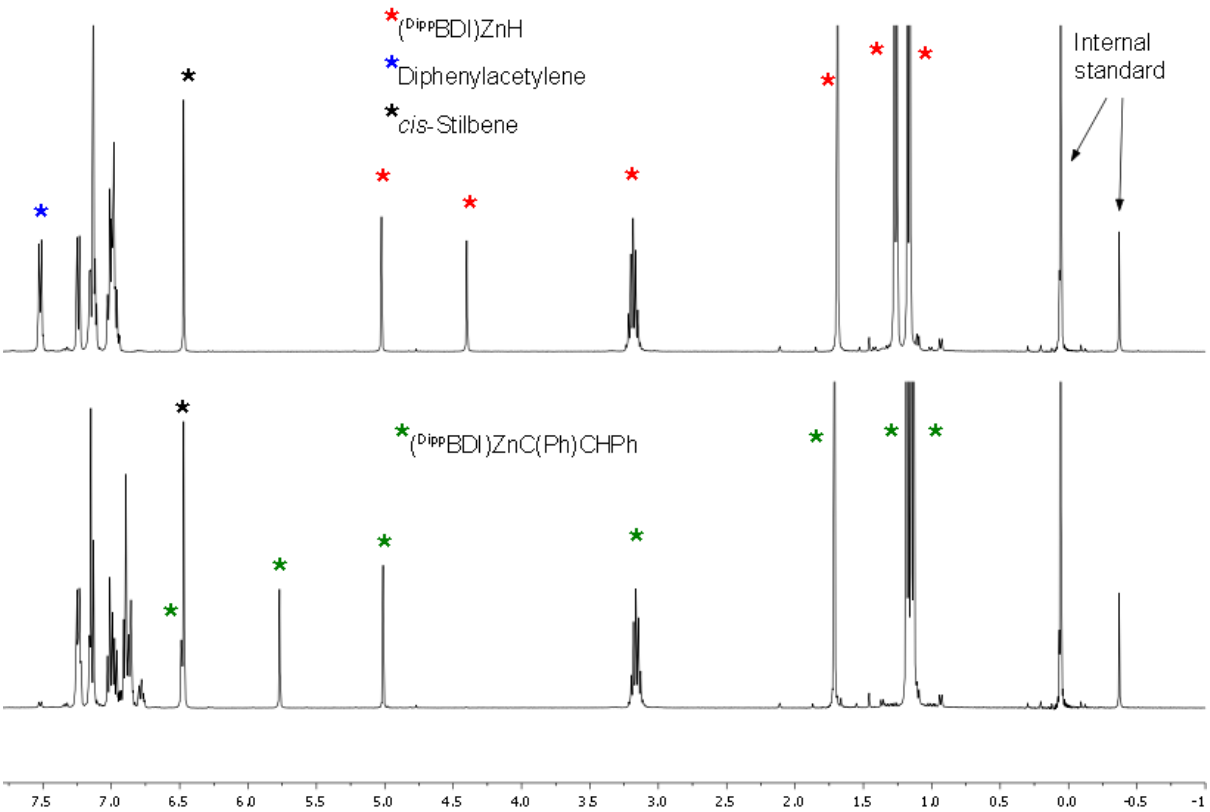

Figure S6. *Initial (top) and final (bottom)* ^{1}*H NMR spectra of alkyne/cis-alkene hydrozincation competition reaction using (*^{Dipp}*BDI)ZnH* ***2****, in* C_6D_6*.*

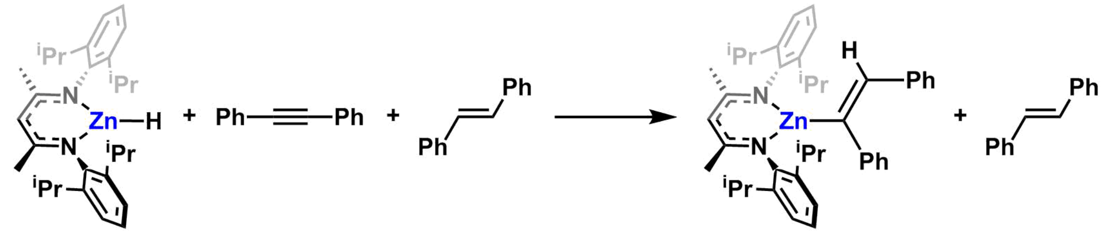

Competitive Alkyne and Alkene Hydrozincation with 2: In a N_2 glovebox, (^{Dipp}BDI)ZnH **2** (7 mg, 0.014 mmol), diphenylacetylene (2.6 mg, 0.014 mmol) and *trans*-stilbene (2.6 mg, 0.014 mmol) were added via C_6D_6 stock solutions to a clean, dry J. Young NMR tube. *Bis*(trimethylsilyl)methane (1 µL, 0.005 mmol) was added directly to the tube as an internal standard. The reaction volume was made up to 0.6 mL with C_6D_6, and the tube was sealed and removed from the glovebox. An initial ^{1}H NMR spectrum was recorded, and then the reaction mixture was heated to 100 °C for 15 h. A final ^{1}H NMR spectrum was recorded.

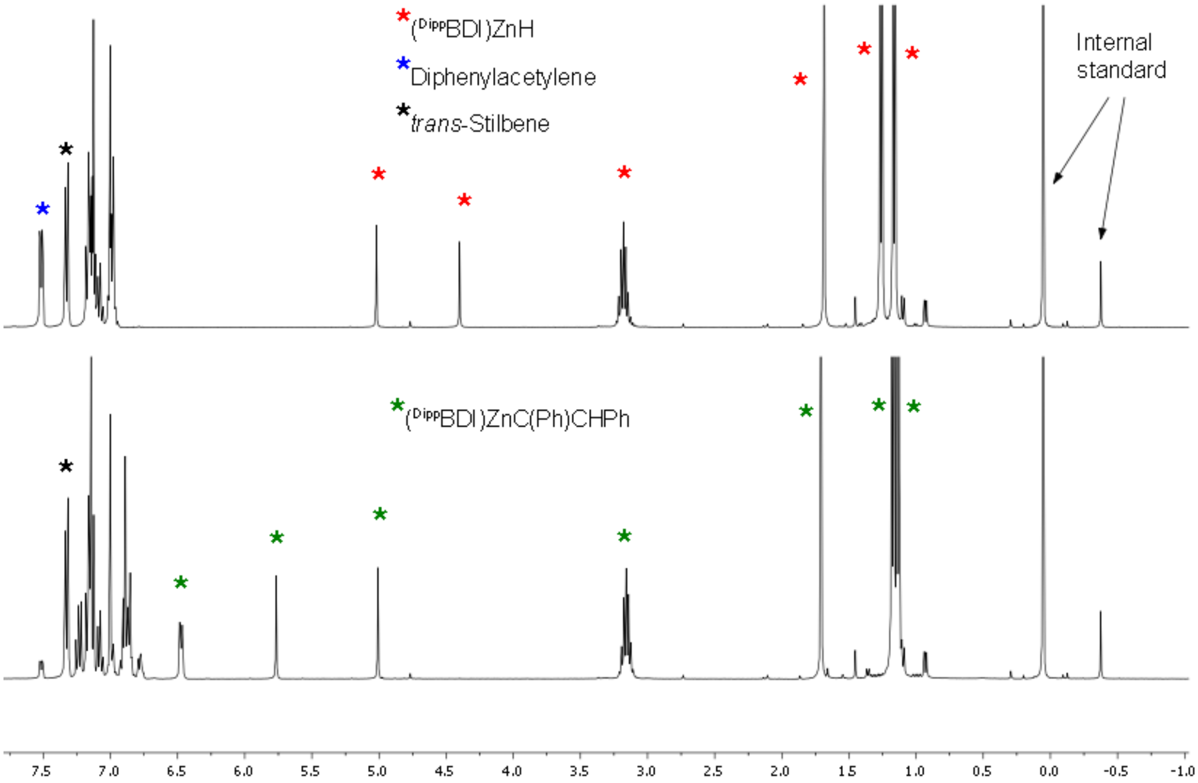

Figure S7. *Initial (top) and final (bottom) ^{1}H NMR spectra of alkyne/trans-alkene hydrozincation competition reaction using (^{Dipp}BDI)ZnH **2**, in C_6D_6.*

2.3 Eyring Analysis for Hydrozincation of Diphenylacetylene with 2

In a N_2 filled glovebox, a 0.1 M stock solution of **2** (50 mg, 0.104 mmol) was prepared in C_6D_6 (1.04 mL), and aliquoted into five J Young NMR tubes. Simultaneously, a 0.5 M stock solution of diphenylacetylene (185.4 mg, 1.04 mmol) was prepared in C_6D_6 (2 mL), and aliquoted into the five NMR tubes. The J Young NMR tubes were equipped with ferrocene capillary tubes to be used as an external standard. The NMR tubes were kept in an ice bath until insertion into a preheated NMR spectrometer (40 – 80 °C). The reaction was monitored by ^{1}H NMR spectroscopy, and known resonances integrated against the external standard. Five rate constants were determined in the temperature range (40 – 80 °C) using the initial rates method, by determining the gradient in the linear section of a concentration vs time plot for each temperature (data was plotted to 50 % conversion). A plot of $\ln(k_{obs}/T)$ against 1/T allowed calculation of the thermodynamic parameters using the Eyring equation.

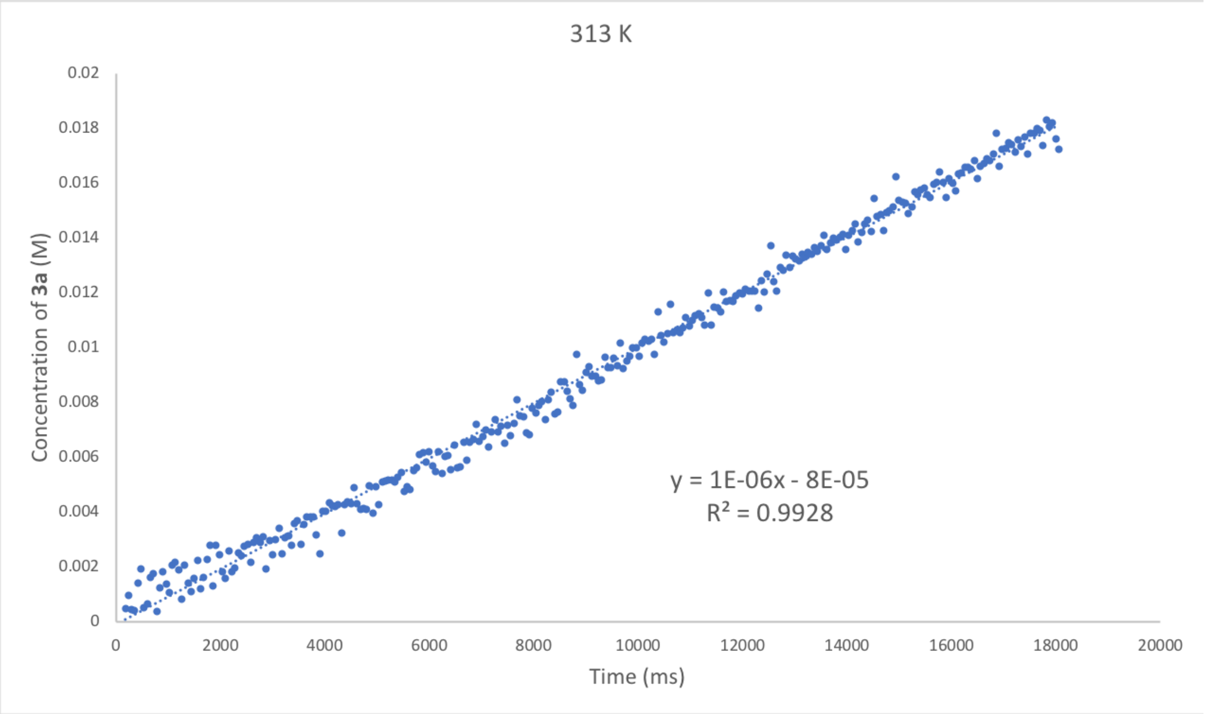

Figure S8. Kinetic data for hydrozincation of diphenylacetylene with 2 at 313 K.

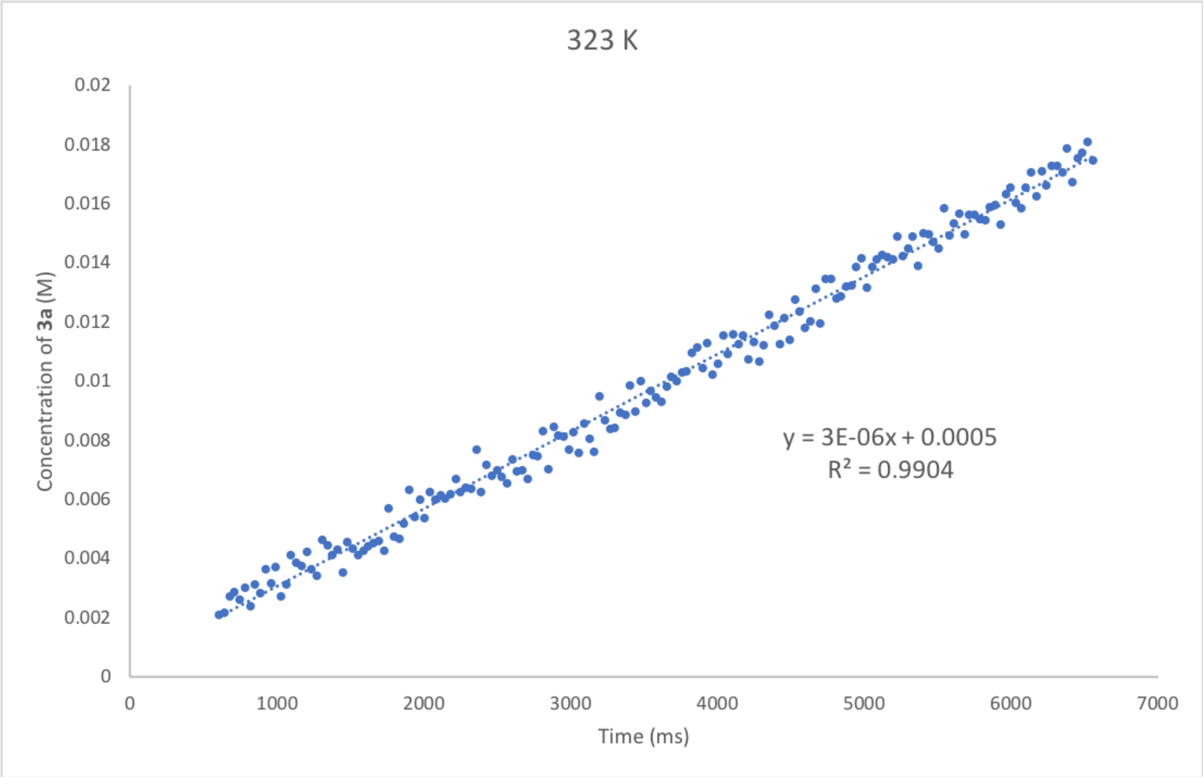

Figure S9. Kinetic data for hydrozincation of diphenylacetylene with 2 at 323 K.

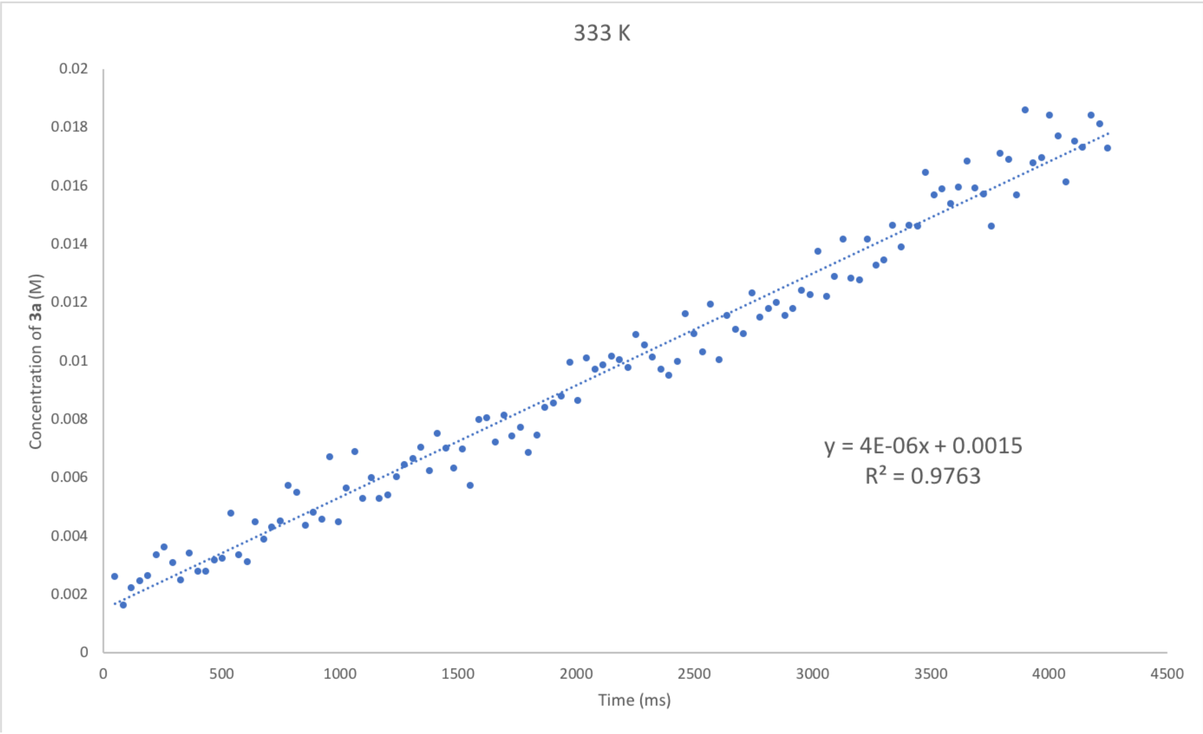

Figure S10. Kinetic data for hydrozincation of diphenylacetylene with 2 at 333 K.

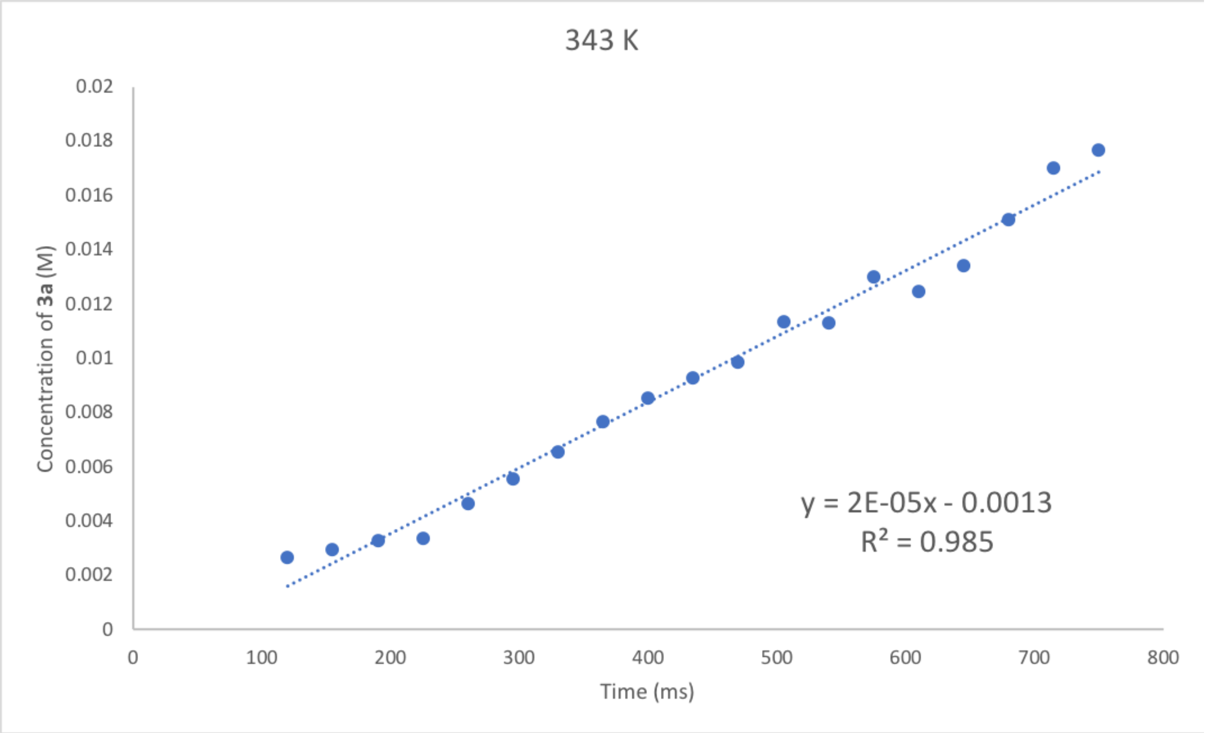

Figure S11. Kinetic data for hydrozincation of diphenylacetylene with 2 at 333 K.

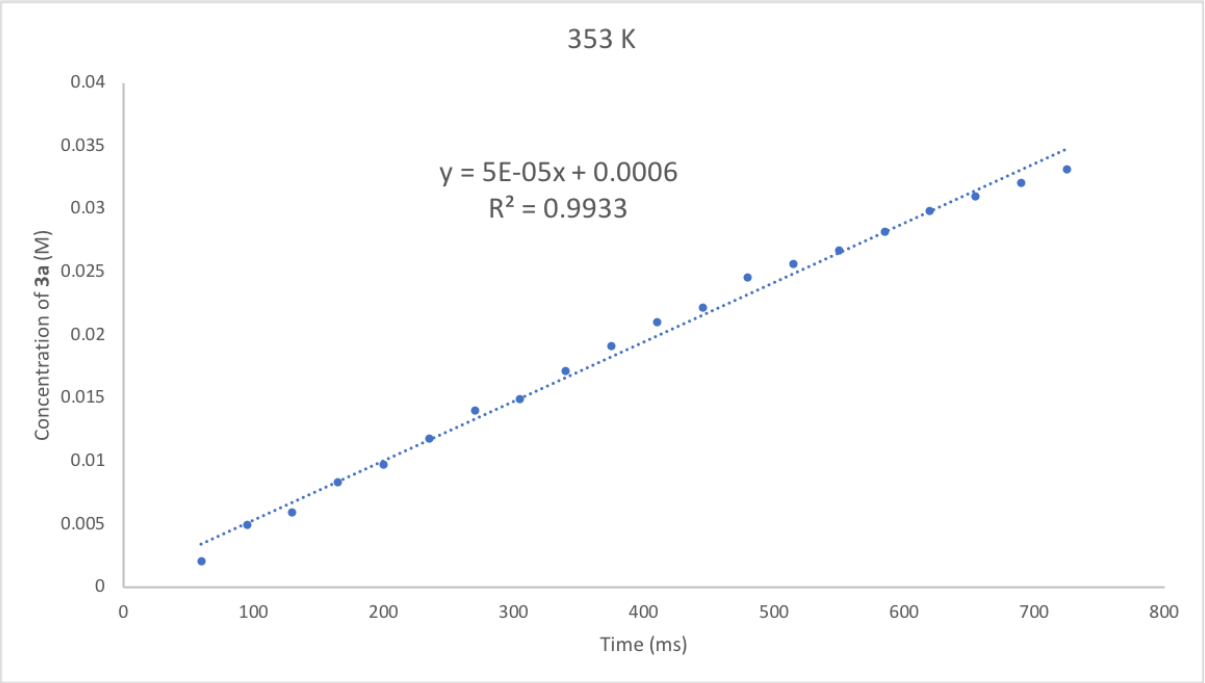

Figure S12. Kinetic data for hydrozincation of diphenylacetylene with 2 at 353 K.

Temperature (K)	1/T	k (s^{-1})	ln(k/T)
313	0.00319	1.00E-06	-19.562
323	0.00310	3.00E-06	-18.495
333	0.00300	4.00E-06	-18.237
343	0.00292	2.00E-05	-16.658
353	0.00283	5.00E-05	-15.770

Table S1. Table of constants derived from kinetic experiments.

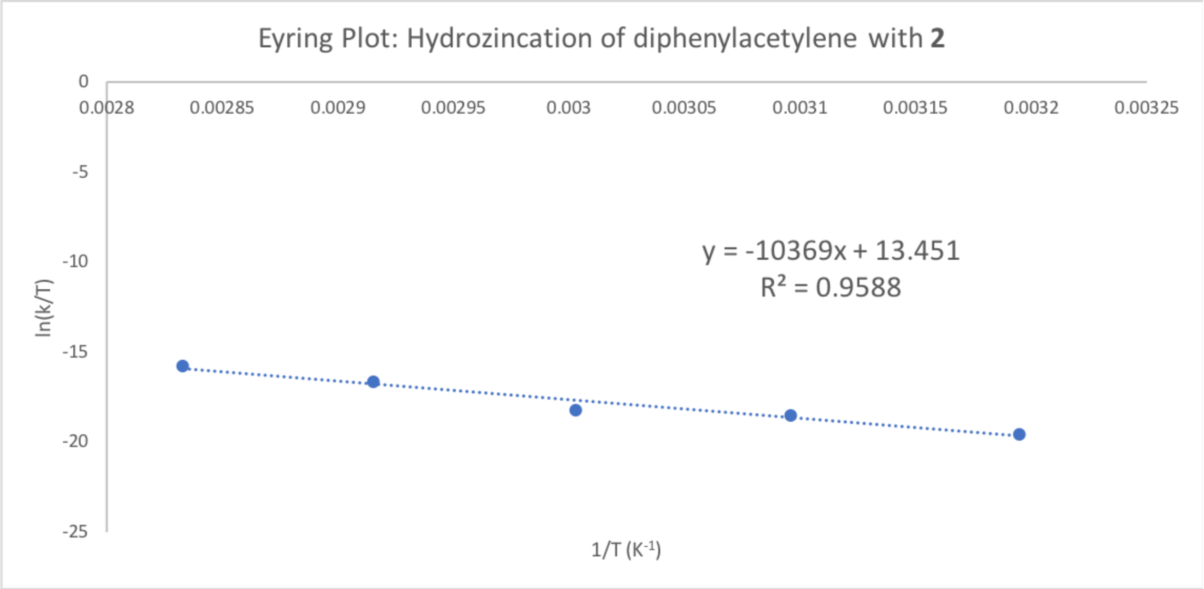

Figure S13. Eyring plot of data from Table S1.

2.4 Investigation of Potential Pd Contamination of Substrates

ICP data were recorded using Perkin Elmer ICP OES 2000 DV, the detection limits from the calibration data are: Palladium (20 µg L^{-1}). Batches of substrates prepared by Pd cross-coupling were subject to ICP analysis to test for trace metals, this analysis was combined with suitable negative control experiments to ensure catalytic activity originated from the Zn-based catalyst.

Table S2. *ICP-OES analysis of substrates synthesised by Pd-catalysis*

Substrate	Pd content by ICP
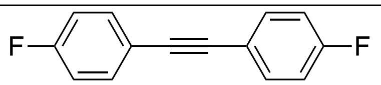	0.0006%
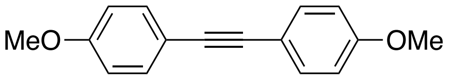	0.0168%

2.5 Catalytic Reaction Procedures

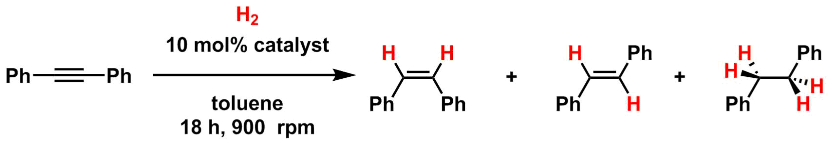

General procedure for high pressure (>10 bar) catalytic reactions: In a N_2 glovebox, **1** and alkyne (10 equiv., 1.3 mmol) were weighed into separate scintillation vials, dissolved in toluene (2 - 7.5 mL) to make a 0.17 M solution. The reaction mixture was transferred to a 25 mL pressure vessel fitted with a PTFE liner. A 0.5 mL aliquot of the reaction mixture was taken for NMR analysis (t_0 vs ferrocene capillary). The pressure vessel was sealed and removed from the glovebox. Inlet and outlet lines were fitted to the vessel, and the inlet valve was purged for 5 minutes with N_2 before the vessel was opened to the gas line. The vessel was then purged with N_2 (10 x 5 bar) followed by H_2 (3 x 3 bar), before being pressurised to 16 bar with H_2 and sealed. The reaction was heated to 145 °C to give a final pressure of 23 bar. Stirring was set to 900 rpm and the reaction was run for 3-18 h. The reactor was cooled to room temperature, H_2 vented, the system purged with N_2. The reactor was then opened and a 0.5 mL aliquot of the reaction mixture was taken for NMR analysis (t_0 vs ferrocene capillary). In cases where NMR analysis proved ineffective for identification and quantification of any alkane side-products (e.g. hydrogenation of hex-3-yne, oct-4-yne) GC-FID analysis was used.

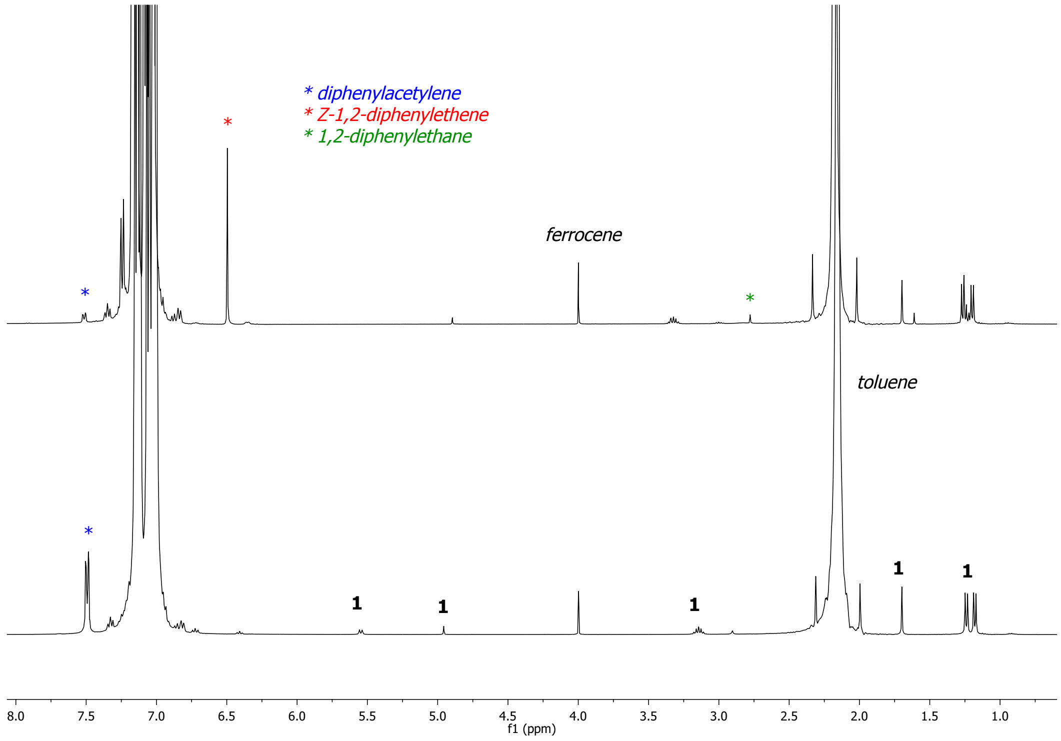

Figure S14. *Initial (top) and final (bottom) ^{1}H NMR spectra of semi-hydrogenation of diphenylacetylene catalysed by **1**, in toluene.*

Table S3. *Alkyne Semi-Hydrogenation Catalysis with Control Reactions.*

Substrate	Catalyst	Volume / mL	Time / h	Conv. / %	Yield / %	*Z : E*	*alkene : alkane*
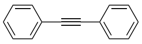	-	2	18	4	4	-	-
	**1** (10 mol%)	**2**	**3**	**84**	**84**	**99 : 1**	**97 : 3**
	**1** (10 mol%)	**2**	**6**	**>99**	**>99**	**99 : 1**	**91 : 9**
	**3a** (10 mol%)	2	3	7	7	99 : 1	99 : 1
	**3a** (10 mol%) + aniline (10 mol%)	2	**4**	**>99**	**>99**	99 : 1	86 : 14
	**4** (10 mol%)	**2**	**18**	**>99**	**>99**	**99 : 1**	**98 : 2**
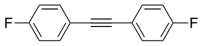	-	2	3	2	2	-	-
	**1** (10 mol%)	**2**	**3**	**43**	**43**	**99 : 1**	**99 : 1**
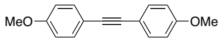	-	2	3	<1	<1	-	-
	**1** (10 mol%)	**2**	**4.5**	**80**	**80**	**99 : 1**	**99 : 1**
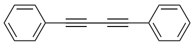	-	7	18	<1	<1	-	-
	**1** (10 mol%)	**7**	**18**	**16**	**16**	**98 : 2**[d]	**99 : 1**
	**1** (10 mol%)	**2.7**	**18**	**25**	**25**	**7:15:2:1**[e]	**99:1**[f]
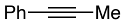	-	7	18	<1	<1		
	**1** (10 mol%)	**7**	**16**	**74**	**74**	**97 : 3**	**95 : 5**
	-	7	18	11	11	92 : 8	91 : 9
	**1** (10 mol%)	**7**	**18**	**96**	**96**	**96 : 4**	**96 : 4**
	-	7	18	<1	<1	-	-
	**1** (10 mol%)	**7**	**18**	**>99**	**>99**	**99 : 1**[c]	**98 : 2**[c]
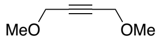	-	7	18	2	2	-	-
	**1** (10 mol%)	**7**	**18**	**92**	**92**	**91 : 9**	**97 : 3**
	-	7	5	2	2	-	-
	**1** (10 mol%)	**7**	**5**	**18**	**18**	**-**	**2 : 1**
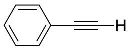	-	30[g]	18	2	2	-	-
	**1** (10 mol%)	**30**[g]	**18**	**9**	**9**	**-**	**99 : 1**[g]
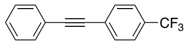	-	2.7	18	<1	<1	-	-
	**1** (10 mol%)	**2.7**	**18**	**>99**	**>99**	**95 : 1**	**95 : 5**
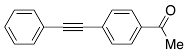	-	2.7	18	>99[h]	>99[h]	1 : 99	99 : 1
	**1** (10 mol%)	**2.7**	**18**	**60**[i]	**20**	**99 : 1**	**99 : 1**
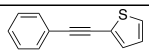	-	2.7	18	5	5	-	-
	**1** (10 mol%)	**2.7**	**18**	**>99**	**95**	**99 : 1**	**83 : 17**
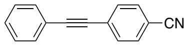	-	2.7	18	5	5	-	-
	**1** (10 mol%)	**2.7**	**18**	**65**	**60**	**99 : 1**	**99 : 1**
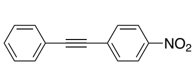	-	2.7	18	5	5	-	-
	**1** (10 mol%)	**2.7**	**18**	**83**[j]	**32**[j]	**99 : 1**	**50 : 50**
	**1** (10 mol%)	**2.7**	**4**	**81**	**67**[k]	**99 : 1**	**66: 33**
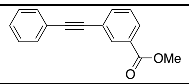	**-**	2.7	18	-	-	-	-
	**1** (10 mol%)	**2.7**	**18**	**10**	**<1**	**-**	**-**
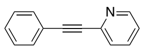	**-**	2.7	18	5	5	-	-
	**1** (10 mol%)	**2.7**	**18**	**30**	**26**	**99 : 1**	**99 : 1**
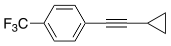	**-**	2.7	18	-	-	-	-
	**1** (10 mol%)	**2.7**	**18**	**25**	**22**	**99 : 1**	**99 : 1**
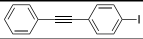	**1** (10 mol%)	**2.7**	**18**	**<5%**	**-**	**-**	**-**

a) The reactions were carried out in an autoclave with H_2, substrate (1.2 mmol) and catalyst (0.12 mmol) in toluene (7 mL) and stirred at 900 rpm for 18 h. b) product distribution measured by NMR. c) product distribution measured by NMR and GC-FID. d) (Z)-enyne to (E)-enyne. e) Z-ene/yne : Z:Z : Z:E : E:E diene. f) ene-yne+diene : alkane, g) reaction conducted in 300 mL Parr 5500 reactor. h) formation of the (E)-alkene product. i) partial reduction of the carbonyl functionality (40 %). j) partial reduction of the NO_2 functionality to the respective amines (ca. 50 %). k) partial reduction of the NO_2 functionality to the respective amines (ca. 17 %).

The products of hydrogenation were assigned by comparison with authentic samples including, cis-stilbene (CAS = 645-49-8), trans-stiblene (CAS = 103-30-0), trans-β-methylstyrene (CAS = 873-66-5), trans- β-methylstyrene (CAS = 766-90-5), cis-hex-3-ene (CAS = 7642-09-3), trans-hex-3-ene (CAS = 13269-528), cis-oct-4-ene (CAS = 7642-15-1), trans-oct-4-ene (CAS = 14850-23-8), hex-1-ene(CAS = 209-753-1), or styrene ((CAS = 100-42-5). This was achieved through spiking of samples used for NMR or GC-FID analysis. Where commercial samples were unavailable hydrogenated products were assigned based on comparison of selected ^{1}H NMR resonances to literature data. In case of substrates for which an assignment of the diagnostic signals directly from the reaction solution was not possible, toluene was evaporated under reduced pressure and the crude reaction mixture analysed by NMR spectroscopy in $CDCl_3$ (ferrocene (10 mol%) was added as an internal standard).

(Z)-1,2-bis(4-fluorophenyl)ethene: ^{1}H NMR (400 MHz, C_6D_6): δ 7.26 (d, 4H, J = 8.2 Hz), 6.70 (d, 4H, J = 8.2 Hz), 6.48 (s, 2H), 3.35 (s, 6H). [Lit.[8] H NMR (300 MHz, $CDCl_3$): δ 7.46 – 7.42 (m, 4H), 6.94 (s, 2H), 6.87 – 6.90 (m, 4H), 3.89 (s, 6H).]

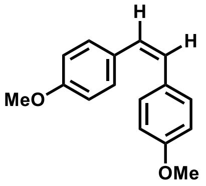

(Z)-1,2-bis(4-methoxyphenyl)ethene: ^{1}H NMR (400 MHz, C_6D_6): δ 6.98 – 6.92 (m, 8H), 6.29 (s, 2H). ^{19}F NMR (400 MHz, C_6D_6): -113.9. [Lit.[8] ^{1}H NMR (300 MHz, $CDCl_3$): δ 7.52 – 7.47 (m, 4H), 7.40 – 7.26 (m, 4H), 6.71 (s, 2H).]

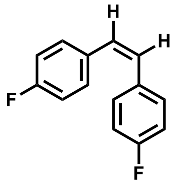

(Z)-1,4-dimethoxybut-2-ene: ^{1}H NMR (400 MHz, C_6D_6): δ 5.70 (t, 2H, J = 3.7 Hz), 3.87 (d, 4H, J = 3.7 Hz), 3.17 (s, 6H). [Lit.[7] ^{1}H NMR (300 MHz, $CDCl_3$): δ 5.72 (t, 2H, J = 4.8 Hz), 4.01 (d, 3H, J = 4.8 Hz, 3.34 (s, 6H).]

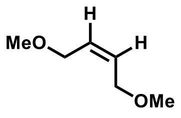

(Z)-1,4-diphenyl-1-buten-3-yne: ^{1}H NMR (400 MHz, C_6D_6): δ 7.95 – 7.92 (d, 2H, J = 7.2 Hz), 7.46 – 7.43 (m, 2H), 6.44 (d, 1H, J = 12.0 Hz), 5.82 (d, 1H, J = 12.0 Hz), remaining

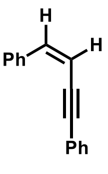

resonances obscured by toluene. [Lit.[9] ^{1}H NMR (400 MHz, $CDCl_3$): δ 7.85 (d, J = 7.4 Hz, 2H), 7.41-7.44 (m, 2H), 7.12-7.30 (m, 6H), 6.63 (d, J = 11.8 Hz, 1H), 5.85 (d, J = 11.8 Hz, 1H).]

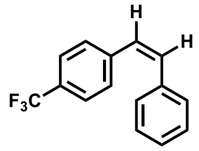

(*Z*)-1-styryl-4-(trifluoromethyl)benzene (diagnostic resonances): ^{1}H NMR (400 MHz, $CDCl_3$) δ 6.77 (d, *J* = 12.2 Hz, 1H, C*H*=CH), 6.64 (d, *J* = 12.2 Hz, 1H, CH=C*H*). ^{13}C NMR (101 MHz, $CDCl_3$) δ 132.4 (1C, *C*H=CH), 128.8 (1C, CH=*C*H). ^{19}F NMR (377 MHz, $CDCl_3$) δ -62.30. [Lit.[10] ^{1}H NMR (400 MHz, $CDCl_3$) δ 6.72 (d, *J* = 12.3 Hz, 1H, C*H*=CH), 6.59 (d, *J* = 12.3 Hz, 1H, CH=C*H*). ^{13}C NMR (101 MHz, $CDCl_3$) δ 132.3 (1C, *C*H=CH), 128.7 (1C, CH=*C*H).]

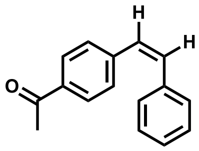

(Z)-1-(4-styrylphenyl)ethanone (diagnostic resonances): ^{1}H NMR (400 MHz, $CDCl_3$) δ 6.76 (d, *J* = 12.2 Hz, 1H, C*H*=CH), 6.64 (d, *J* = 12.2 Hz, 1H, C*H*=CH), 2.59 (s, 2H, C*H*$_3$). ^{13}C NMR (101 MHz, $CDCl_3$) δ 132.5 (1C, *C*H=CH), 129.2 (1C, CH=*C*H), 26.6 (1C, *C*H$_3$). [Lit.[11] ^{1}H NMR (400 MHz, $CDCl_3$) δ 6.73 (d, *J* = 12.4 Hz, 1H, C*H*=CH), 6.61 (d, *J* = 12.4 Hz, 1H, CH=C*H*). ^{13}C NMR (101 MHz, $CDCl_3$) δ 132.4 (1C, *C*H=CH), 129.1 (1C, CH=*C*H), 26.6 (1C, *C*H$_3$).]

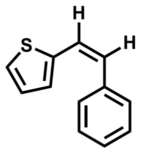

(Z)-2-Styrylthiophene (diagnostic resonances): ^{1}H NMR (400 MHz, $CDCl_3$) δ 6.75 (d, *J* = 12.0 Hz, 1H, C*H*=CH), 6.63 (d, *J* = 12.0 Hz, 1H, C*H*=CH). ^{13}C NMR (101 MHz, $CDCl_3$) δ 128.9 (1C, *C*H=CH), 123.4 (1C, CH=*C*H). [Lit.[12] ^{1}H NMR (300 MHz, $CDCl_3$) δ 6.77 (d, *J* = 11.7 Hz, 1H, C*H*=CH), 6.64 (d, *J* = 11.7 Hz, 1H, C*H*=CH). ^{13}C NMR (75 MHz, $CDCl_3$) δ 129.1 (1C, *C*H=CH), 123.6 (1C, CH=*C*H).]

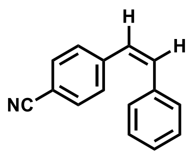

(*Z*)-4-styrylbenzonitrile (diagnostic resonances): ^{1}H NMR (400 MHz, $CDCl_3$) δ 6.80 (d, *J* = 12.2 Hz, 1H, C*H*=CH), 6.60 (d, *J* = 12.2 Hz, 1H, CH=C*H*). ^{13}C NMR (101 MHz, $CDCl_3$) δ 133.3 (1C, *C*H=CH), 127.9 (1C, CH=*C*H). [Lit.[10] ^{1}H NMR (400 MHz, $CDCl_3$) δ 6.79 (d, *J* = 12.2 Hz, 1H, C*H*=CH), 6.60 (d, *J* = 12.2 Hz, 1H, CH=C*H*). ^{13}C NMR (101 MHz, $CDCl_3$) δ 133.3 (1C, *C*H=CH), 127.8 (1C, CH=*C*H).]

(*Z*)-1-nitro-4-styrylbenzene (diagnostic resonances): ^{1}H NMR (400 MHz, $CDCl_3$) δ 6.85 (d, *J* = 12.2 Hz, 1H, C*H*=CH), 6.65 (d, *J* = 12.2 Hz, 1H, CH=C*H*). ^{13}C NMR (101 MHz, $CDCl_3$) δ 134.0 (1C, *C*H=CH), 128.3 (1C, CH=*C*H). [Lit.[13] ^{1}H NMR (300 MHz, $CDCl_3$) δ 6.81 (d, *J* = 12.1 Hz, 1H, C*H*=CH), 6.61 (d, *J* = 12.1 Hz, 1H, CH=C*H*). ^{13}C NMR (75 MHz, $CDCl_3$) δ 134.0 (1C, *C*H=CH), 128.1 (1C, CH=*C*H).]

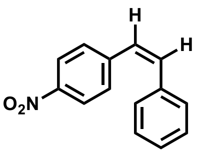

(Z)-2-styrylpyridine (diagnostic resonances): ^{1}H NMR (400 MHz, $CDCl_3$) δ 6.84 (d, *J* = 12.3 Hz, 1H, C*H*=CH), 6.73 (d, *J* = 12.3 Hz, 1H, CH=C*H*). ^{13}C NMR (101 MHz, $CDCl_3$) δ 133.4 (1C, *C*H=CH), 130.4 (1C, CH=*C*H). [Lit.[13] ^{1}H NMR (400 MHz, $CDCl_3$) δ 6.82 (d, *J* = 12.3 Hz, 1H, C*H*=CH), 6.68 (d, *J* = 12.3 Hz, 1H, CH=C*H*). ^{13}C NMR (101 MHz, $CDCl_3$) δ 133.3 (1C, *C*H=CH), 130.5 (1C, CH=*C*H).]

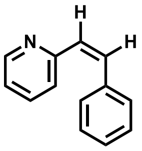

(*Z*)-2-cyclopropylethenyl-4-(trifluoromethyl)benzene (diagnostic resonances): ^{1}H NMR (400 MHz, $CDCl_3$) δ 6.38 (d, *J* = 11.5 Hz, 1H, CH=C*H*), 5.20 (dd, *J* = 11.5, 10.0 Hz, 1H, C*H*=CH), 1.87 (m, 1H, CH_2C*H*CH_2). ^{19}F NMR (377 MHz, $CDCl_3$) δ -62.37 (3F). ^{13}C NMR (101 MHz, $CDCl_3$) δ 139.0 (1C, *C*H=CH), 126.1 (1C, CH=*C*H), 11.1 (1C, CH_2*C*HCH_2).

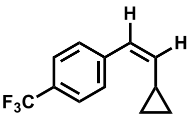

(*Z*)-dodec-5-en-7-yne: 5.80 (dt , *J* = 10.8, 7.2 Hz, 1H), 5.41-5.45 (m, 1H) [Lit.[14] ^{1}H NMR (500 MHz, $CDCl_3$, δ, ppm): 0.87-0.95 (m, 6H), 1.28-1.56 (m, 8H), 2.26-2.37 (m, 4H), 5.41-5.45 (m, 1H), 5.80 (dt , *J* = 10.8, 7.2 Hz, 1H)]. **(5Z,7Z)-dodeca-5,7-diene**: ^{1}H NMR (400 MHz, C_6D_6): δ 6.40 (m, 2H), 5.48 (m, 2H), 0.90 (m,3H) [Lit.[14] ^{1}H NMR (400 MHz, C_6D_6): δ 6.39 (m, 2H), 5.46 (m, 2H), 2.15 (m, 4H), 1.29 (m, 8H), 0.85 (t, J = 7.1 Hz, 3H).]

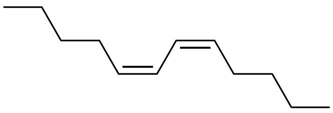

Effect of H_2 pressure: In a N_2 glovebox, **1** (26 mg, 0.046 mmol) and diphenylacetylene (82 mg, 0.46 mmol) were weighed into separate scintillation vials, dissolved in toluene (2.7 mL) to make a 0.17 M solution. The reaction mixture was transferred to a 25 mL pressure vessel fitted with a PTFE liner. A 0.5 mL aliquot of the reaction mixture was taken for NMR analysis (t_0 vs ferrocene capillary). The pressure vessel was sealed and removed from the glovebox. Inlet and outlet lines were fitted to the vessel, and the inlet valve was purged for 5 minutes with N_2 before the vessel was opened to the gas line. The vessel was then purged with N_2 (10 x 5 bar) followed by H_2 (3 x 3 bar), before being pressurised to 3.6, 7.2, 10.8 or 14.4 bar with H_2 and sealed. The reaction was heated to 140 °C to give a final pressure of 5, 10, 15 or 20 bar, respectively. Stirring was set to 900 rpm and the reaction was

run for 18 h. The reactor was cooled to room temperature, H_2 vented, the system purged with N_2. The reactor was then opened and a 0.5 mL aliquot of the reaction mixture was taken for NMR analysis (t_0 vs ferrocene capillary).

Table S4. *Semi-hydrogenation of diphenylacetylene at different pressures of H_2.*

P (bar)	% Conversion	TOF_{18h} (h^{-1})
5	3.5	0.019
10	12	0.067
15	74	0.411
20	99	0.550

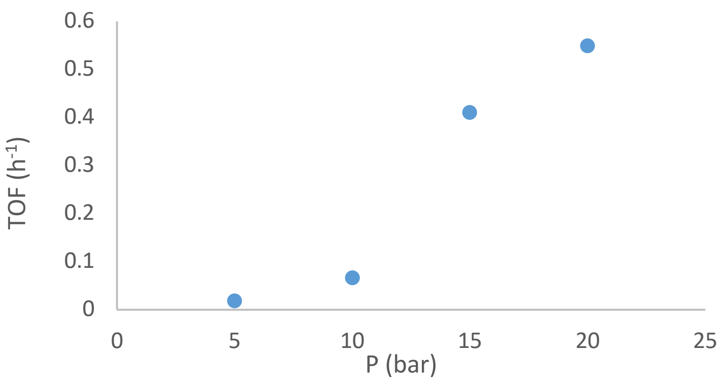

Figure S15*. TOF (h^{-1}) versus H_2 pressure (bar) plot for of semi-hydrogenation of diphenylacetylene catalysed by **1**, in toluene.*

Effect of initial alkyne concentration: In a N_2 glovebox, **1** (26 mg, 0.046 mmol) and diphenylacetylene (82, 205, 327 or 410 mg, 0.46, 1.15, 1.84 or 2.3 mmol respectively) were weighed into separate scintillation vials, dissolved in toluene (2.7 mL). The reaction mixture was transferred to a 300 mL pressure vessel fitted with four glass vials equipped with a stirrer bar and septum pierced by a needle (**Note**: this reactor setup allowed reactions to be run in parallel and likely has different gas mass transfer compared with the 25 Ml reactor used to investigate H2 pressure and for substrate screening). A 0.5 mL aliquot of the reaction mixture was taken for NMR analysis (t_0 vs ferrocene capillary). The pressure vessel was sealed and removed from the glovebox. Inlet and outlet lines were fitted to the vessel, and the inlet valve was purged for 5 minutes with N_2 before the vessel was opened to the gas line. The vessel was then purged with N_2 (10 x 5 bar) followed by H_2 (3 x 3 bar), before being

pressurised to 10.8 bar with H_2 and sealed. The reaction was heated to 160 °C to give a final pressure of 18 bar. Stirring was set to 900 rpm and the reaction was run for 3 h. The reactor was cooled to room temperature, H_2 vented, the system purged with N_2. The reactor was then opened and a 0.5 mL aliquot of the reaction mixture was taken for NMR analysis (t_0 vs ferrocene capillary).

Table S5. *Semi-Hydrogenation of diphenylacetylene at different initial concentration of alkyne.*

**[Alkyne]$_0$ (M)**	**% Conversion**	**TOF$_{3h}$*10^{-3} (h^{-1})**
0.17	13	0.917
0.43	9	1.587
0.68	3	0.846
0.85	1	0.353

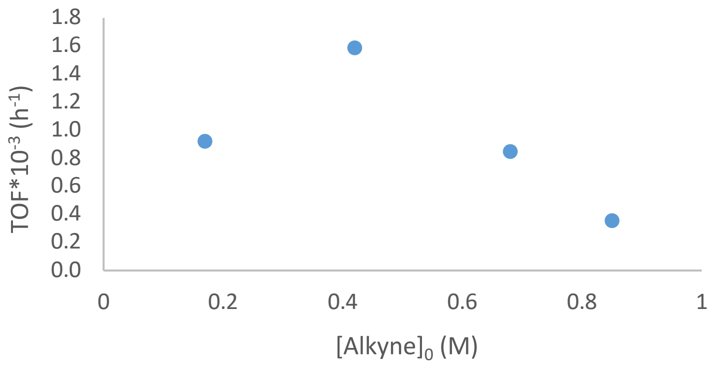

Figure S16*. TOF*10^{-3} (h^{-1}) versus initial alkyne concentration (M) plot for of semi-hydrogenation of diphenylacetylene catalysed by* ***1****, in toluene.*

3. X-Ray Crystallography Data

Table S6*. Crystal Data, Data Collection and Refinement Parameters for the structures of* ***1, 3a, 3b, 3c, 3d, 3e, 3f*** *and* ***4****.*

data	1	3a	3b	3c
**formula**	$C_{35}H_{47}N_3Zn$	$C_{43}H_{52}N_2Zn$	$C_{37}H_{56}N_2Zn$	$C_{38}H_{50}N_2Zn$
**solvent**	—	—	—	—
**formula weight**	575.12	662.23	594.20	600.17
**colour, habit**	colourless laths	colourless [illegible]	colourless [illegible]	colourless [illegible]
**temperature / K**	173	173	173	173
**crystal system**	monoclinic	monoclinic	monoclinic	triclinic
**space group**	$P2_1/c$ (no. 14)	$P2_1/n$ (no. 14)	$I2/a$ (no. 15)	P-1 (no. 2)
a **/ Å**	18.4765(5)	11.3784(2)	29.6965(10)	11.2481(9)
b **/ Å**	8.6228(3)	15.3573(3)	11.3418(4)	12.7827(10)
c **/ Å**	40.7573(16)	21.4017(4)	21.1045(7)	12.9402(11)
**α / deg**	90	90	90	109.689(7)
**β / deg**	99.404(3)	92.5051(17)	94.219(3)	91.861(7)
**γ / deg**	90	90	90	99.363(6)
V **/ Å^3**	6406.1(4)	3736.18(13)	7089.0(4)	1720.8(2)
Z	8 [c]	4	8	2
D_c **/ g cm^{-3}**	1.193	1.177	1.114	1.158
**radiation used**	Mo-Kα	Mo-Kα	Mo-Kα	Mo-Kα
**μ / mm^{-1}**	0.793	0.688	0.718	0.740
**no. of unique reflns**				
**measured (R_{int})**	12822 (0.0314)	7500 (0.0187)	7076 (0.0225)	7249 (0.0736)
**obs, $\|F_o\| > 4\sigma(\|F_o\|)$**	9058	5912	5684	5701
**completeness (%)** [a]	98.4	98.9	98.6	99.7
**no. of variables**	731	436	404	403
**R_1(obs), wR_2(all)** [b]	0.0440, 0.1040	0.0368, 0.0921	0.0397, 0.1094	0.0667, 0.1900

Table S6. ...*part 2*

data	3d	3e	3f	4
**formula**	$C_{38}H_{50}N_2Zn$	$C_{45}H_{52}N_2Zn$	$C_{38}H_{52}N_2Zn$	$C_{39}H_{54}ClLiN_3O_2Zn$
**solvent**	—	—	—	C_4H_8O
**formula weight**	600.17	686.25	602.18	776.71
**colour, habit**	colourless	colourless	colourless	colourless
**temperature / K**	173	173	173	173
**crystal system**	orthorhombic	monoclinic	monoclinic	orthorhombic
**space group**	$P2_12_12_1$ (no. 19)	$P2_1/n$ (no. 14)	$I2/a$ (no. 15)	$Cmc2_1$ (no. 36)
***a* / Å**	10.4541(4)	9.3656(5)	22.6811(16)	19.6080(4)
***b* / Å**	14.8464(8)	31.3659(12)	9.2897(3)	14.4047(3)
***c* / Å**	22.2664(8)	13.3287(5)	34.085(3)	14.9310(3)
**α / deg**	90	90	90	90
**β / deg**	90	99.694(4)	103.913(8)	90
**γ / deg**	90	90	90	90
***V* / Å^3**	3455.9(3)	3859.5(3)	6971.0(8)	4217.21(14)
Z	4	4	8	4 [d]
**D_c / g cm^{-3}**	1.154	1.181	1.148	1.223
**radiation used**	Mo-Kα	Mo-Kα	Mo-Kα	Cu-Kα
**μ / mm^{-1}**	0.737	0.668	0.731	1.695
**no. of unique reflns**				
**measured (R_{int})**	6004 (0.0395)	7720 (0.0234)	6983 (0.0319)	2886 (0.0231)
**obs, $\|F_o\| > 4\sigma(\|F_o\|)$**	5230	5959	5529	2743
**completeness (%)** [a]	98.6	98.7	98.8	99.1
**no. of variables**	381	443	431	286
**R_1(obs), wR_2(all)** [b]	0.0403, 0.0895	0.0447, 0.1021	0.0420, 0.1092	0.0334, 0.0853

[a] $R_1 = \Sigma||F_o| - |F_c||/\Sigma|F_o|$; $wR_2 = \{\Sigma[w(F_o^2 - F_c^2)^2] / \Sigma[w(F_o^2)^2]\}^{1/2}$; $w^{-1} = \sigma^2(F_o^2) + (aP)^2 + bP$. [c] There are two crystallographically independent complexes. [d] The molecule has crystallographic C_S symmetry.

Table S6 provides a summary of the crystallographic data for the structures of **1**, **3a**, **3b**, **3c**, **3d**, **3e**, **3f** and **4**. The absolute structure of **4** was determined by use of the Flack parameter [$x^+ = 0.00(3)$]. CCDC 2168747 (**1**), 2004167 (**3a**), 2004168 (**3b**), 2168748 (**3c**), 2168749 (**3d**), 2168750 (**3e**), 2168751 (**3f**) and 2004169 (**4**).

The structure of **1** was found to contain two crystallographically independent complexes (**1-A** and **1-B**) in the asymmetric unit. The N30–H hydrogen atoms of both independent complexes were located from Δ*F* maps and refined freely subject to N–H distance constraints of 0.90 Å.

The C24-based isopropyl group in the structure of **3a** was found to be disordered. Two orientations were identified of *ca*. 56 and 44% occupancy, their geometries were optimised, the thermal parameters of adjacent atoms were restrained to be similar, and only the non-hydrogen atoms of the major occupancy orientation were refined anisotropically (those of the minor occupancy orientation were refined isotropically).

The C31-based oct-4-yne group in the structure of **3b** was found to be disordered. Two orientations were identified of *ca*. 73 and 27% occupancy, their geometries were optimised, the thermal parameters of adjacent atoms were restrained to be similar, and only the non-hydrogen atoms of the major occupancy orientation were refined anisotropically (those of the minor occupancy orientation were refined isotropically).

Reciprocal space analysis of the data set for the structure of **3c** clearly showed the crystal to be severely twinned, with the initial indexing using only *ca*. 40% of the observed spots. Unfortunately, despite numerous efforts, attempts to model this twinning proved unsatisfactory, with the best results coming from the standard, non-twin, data processing. The C12- and C24-based isopropyl groups were both found to be disordered, and in each case two orientations were identified, of *ca*. 81:19 and 71:29% occupancy respectively. The geometries of each pair of orientations were optimised, the thermal parameters of adjacent atoms were restrained to be similar, and only the non-hydrogen atoms of the major occupancy orientations were refined anisotropically (those of the minor occupancy orientations were refined isotropically).

The structure of **3f** was found to be highly disordered, with only the eight core atoms of the zinc beta-diketiminate moiety (ring system + methyl carbon atoms) and the first atom of the C31-based propylphenyl ligand being ordered. Two orientations were identified for each of the C6- and C18-based 2,6-diisopropylphenyl groups and for the C32-based ethyl benzene unit of *ca*. 61:39, 72:28 and 62:38% occupancy respectively. The geometries of each pair of orientations were optimised, the thermal parameters of adjacent atoms were restrained to be similar, and only the non-hydrogen atoms of the major occupancy orientations were refined anisotropically (those of the minor occupancy orientations were refined isotropically).

The structure of **4** was found to sit across a mirror plane that passes through C2, Zn1, Cl1 and Li1, and bisects the N1···N1A vector. Both the N20-based aniline ligand and the O40-based thf solvent molecule were found to be disordered across this mirror plane, and in each case this was modelled by using one complete, 50% occupancy orientation, with a further orientation of the same occupancy being generated by operation of the mirror plane. For both cases the geometry of the unique orientation was optimised, and all the non-hydrogen atoms were refined anisotropically (for the O40-based thf solvent molecule the thermal parameters of adjacent atoms were additionally restrained to be similar).

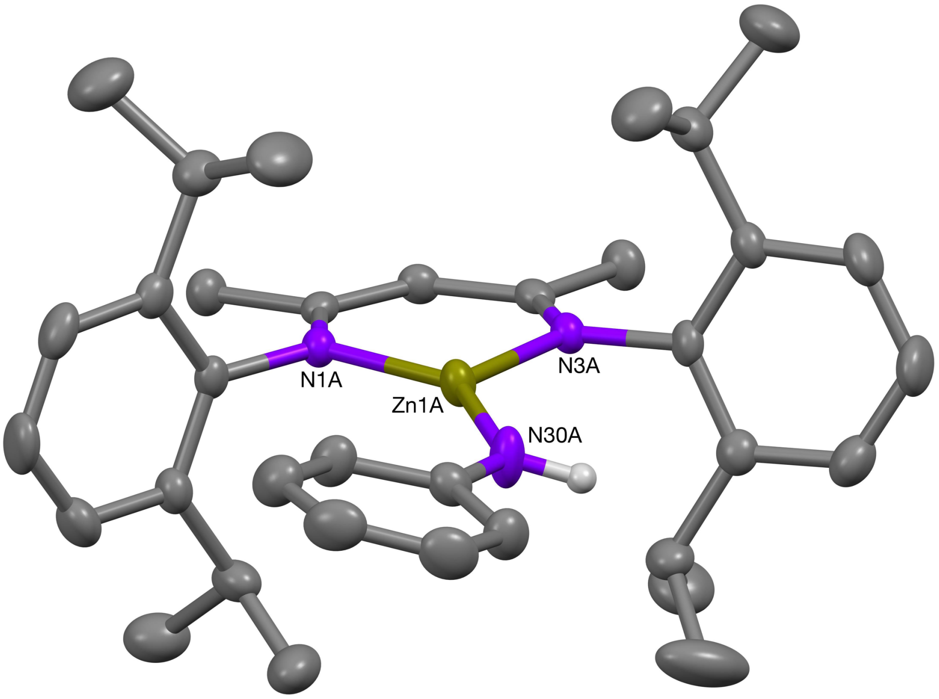

Figure S17. *The structure of **1-A**, one of the two independent complexes present in the crystal of **1** (50% probability ellipsoids).*

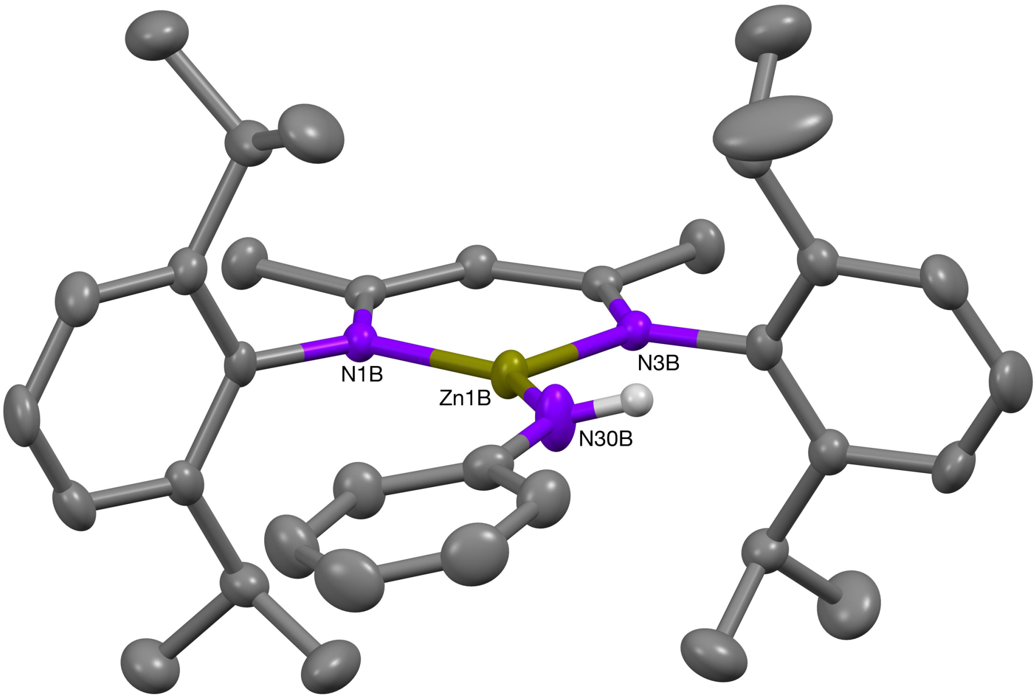

Figure S18. *The structure of **1-B**, one of the two independent complexes present in the crystal of **1** (50% probability ellipsoids).*

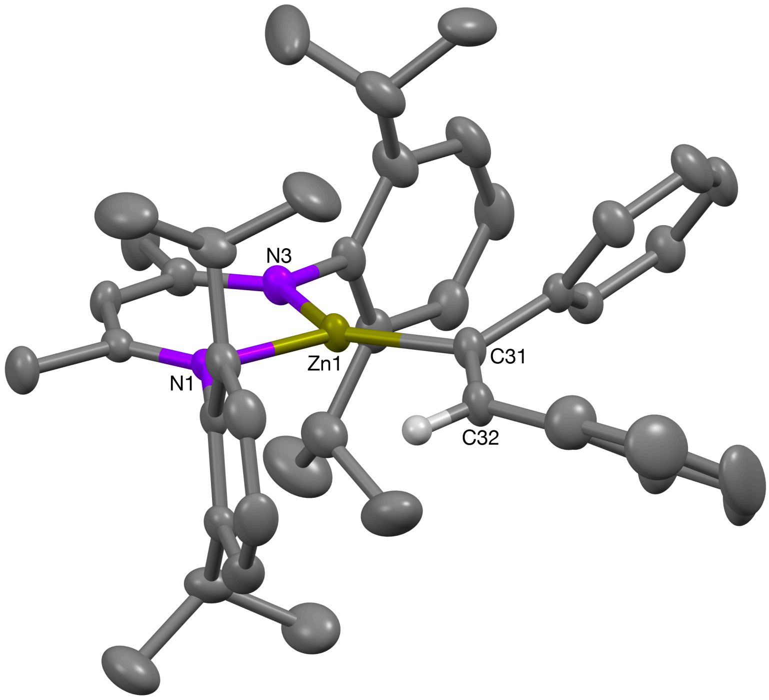

Figure S19. *The crystal structure of **3a** (50% probability ellipsoids).*

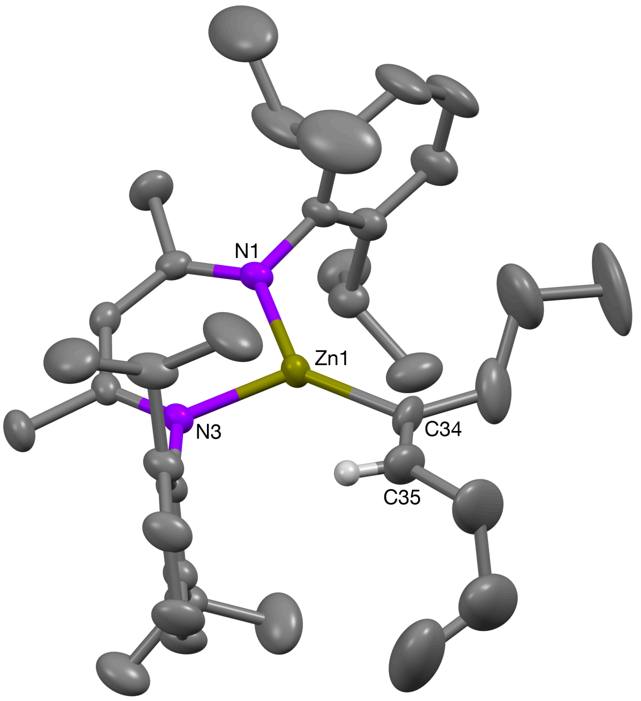

Figure S20. *The crystal structure of **3b** (50% probability ellipsoids).*

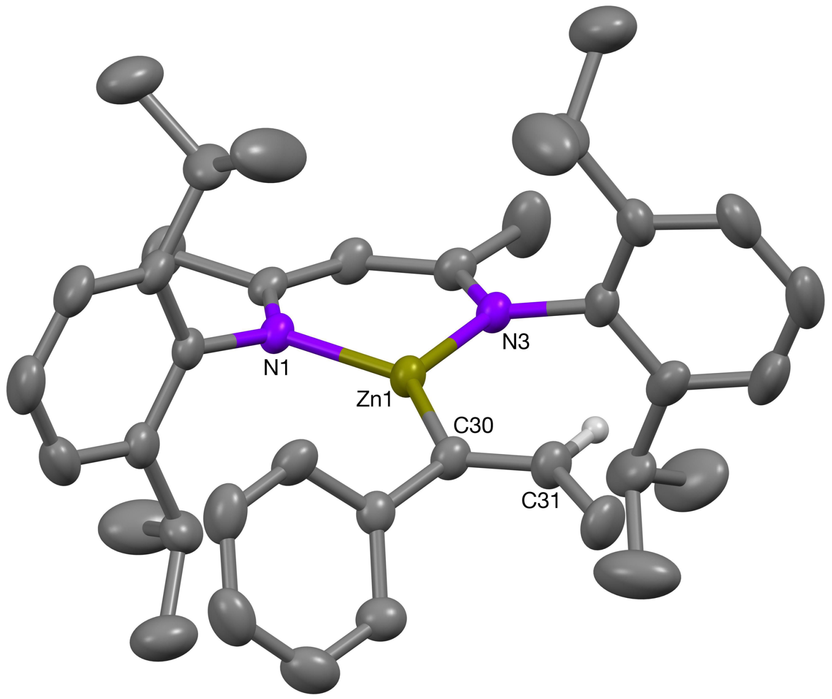

Figure S21. *The crystal structure of **3c** (50% probability ellipsoids).*

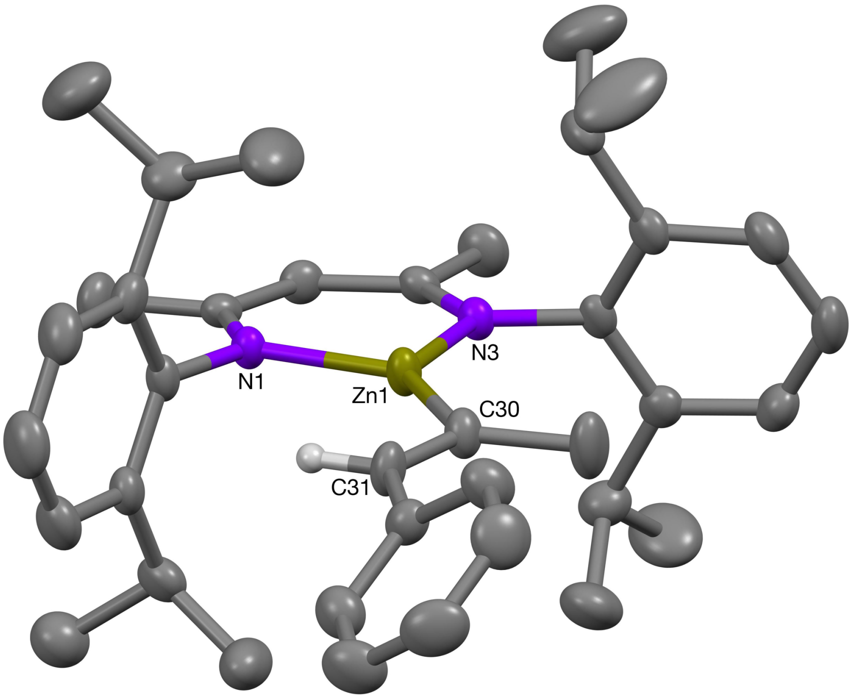

Figure S22. *The crystal structure of* ***3d*** *(50% probability ellipsoids).*

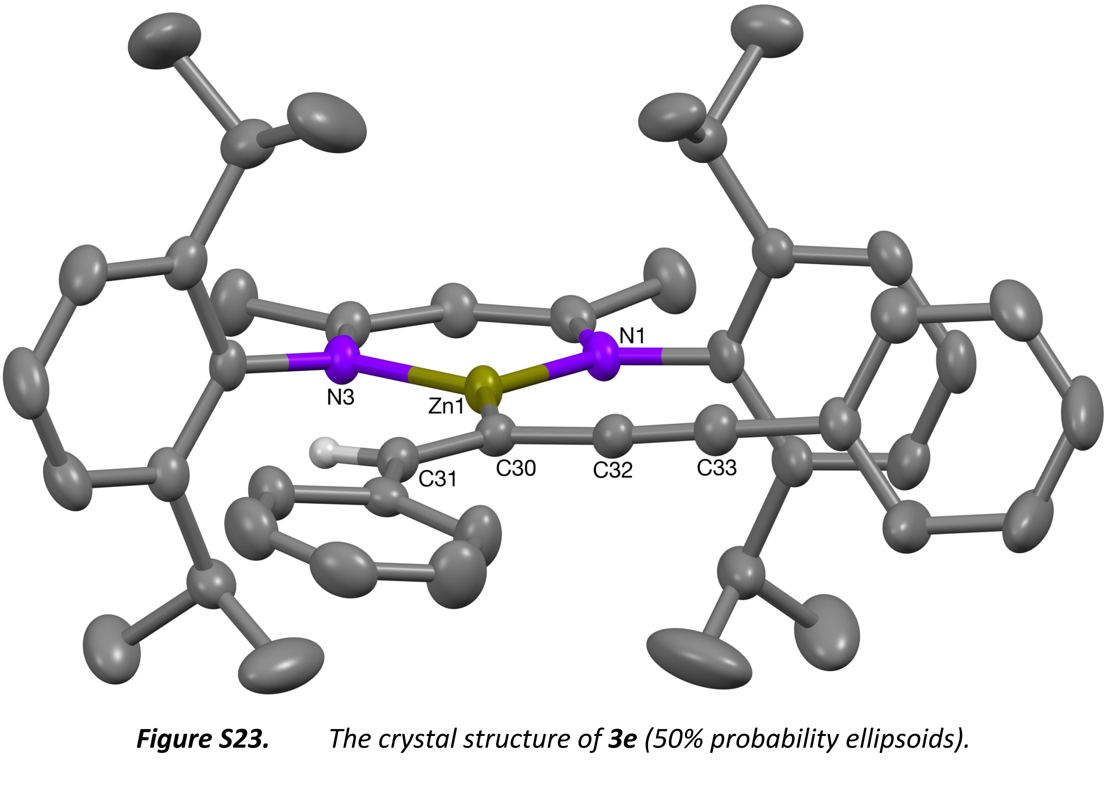

Figure S23. *The crystal structure of* ***3e*** *(50% probability ellipsoids).*

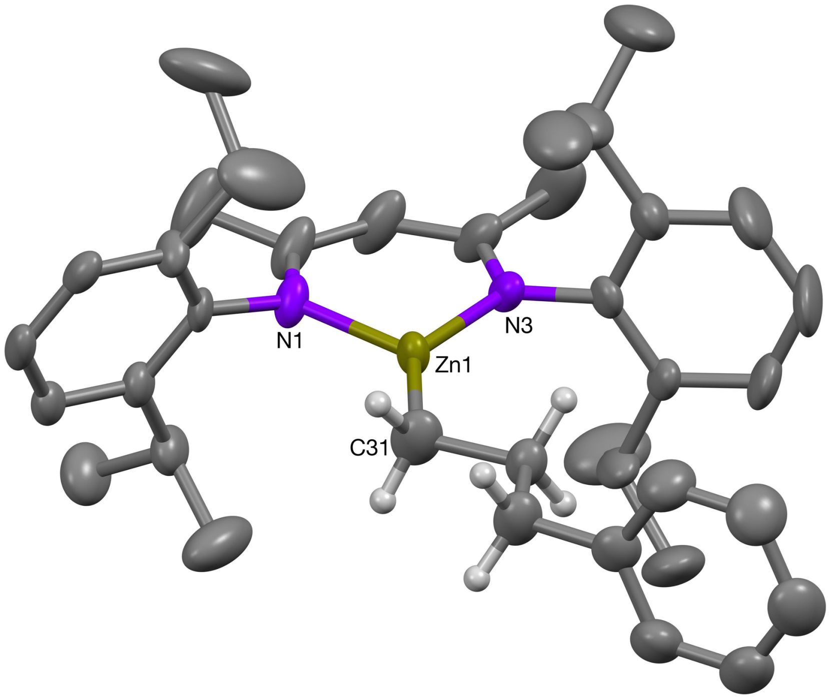

Figure S24. *The crystal structure of **3f** (50% probability ellipsoids).*

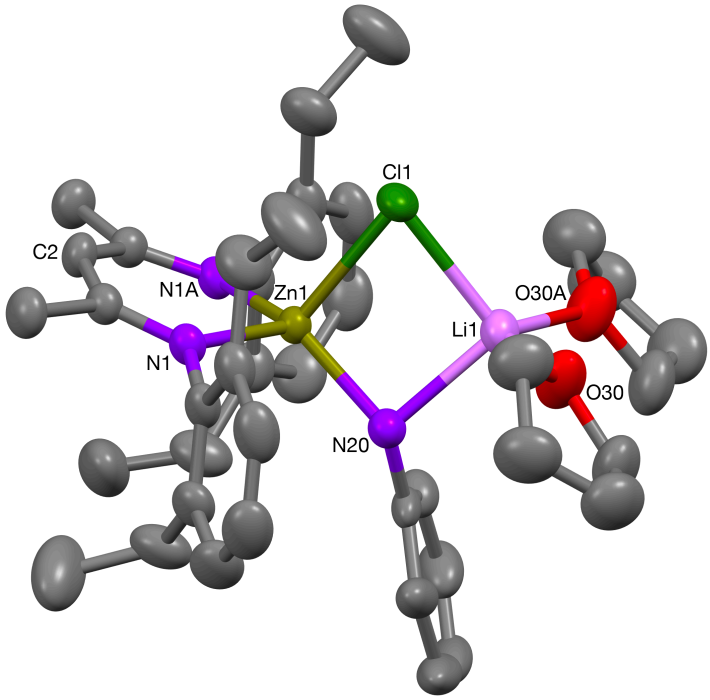

Figure S25. *The crystal structure of **4** (50% probability ellipsoids). The structure sits across a mirror plane that passes through C2, Zn1, Cl1 and Li1, and bisects the N1···N1A vector; the N20-based aniline ligand is disordered across this mirror plane.*

4. Computational Studies

DFT calculations were run using Gaussian 09 (Revision D.01)[15] using the M06L density functional.[16] Zn centres were described with Stuttgart SDDAll RECPs and associated basis sets, 6-31G** basis sets were used for C and H atoms and 6-311+G* basis sets were used for N atoms.[17-19]

The geometries of products were optimised with the M06L hybrid exchange-correlation DFT functional using the Gaussian09 program package. Frequency analyses for all stationary points were performed using the enhanced criteria to confirm the nature of the structures as either minima (no imaginary frequency) or transition states (only one imaginary frequency). Intrinsic reaction coordinate (IRC) calculations followed by full geometry optimisations on final points were used to connect transition states and minima located on the potential energy surface allowing a full energy profile (calculated at 298.15 K, 1 atm) of the reaction to be constructed.[20-21] Free energies reported within the main text are corrected for the effects of benzene solvent (ε=2.2706) using the polarizable continuum model (PCM).[22] In addition, single point dispersion corrections were applied to the M06L optimised geometries employing Grimme's D3 correction.[23] We also tested two other DFT functionals, these were the B3PW91 and ωB97X functionals. For this functional testing single point dispersion corrections were applied to the optimised geometries using Grimme's D3 correction with Becke-Johnson damping (EmpiricalDispersion=GD3BJ) for B3PW91[24] and Grimme's D2 correction for ωB97X (using the keyword wB97XD).[25] The graphical user interface used to visualise the various properties of the intermediates and transition states was GaussView 5.0.9.[26] Natural Bond Orbital analysis was carried out using NBO 6.0.[27]

Calculated Structures

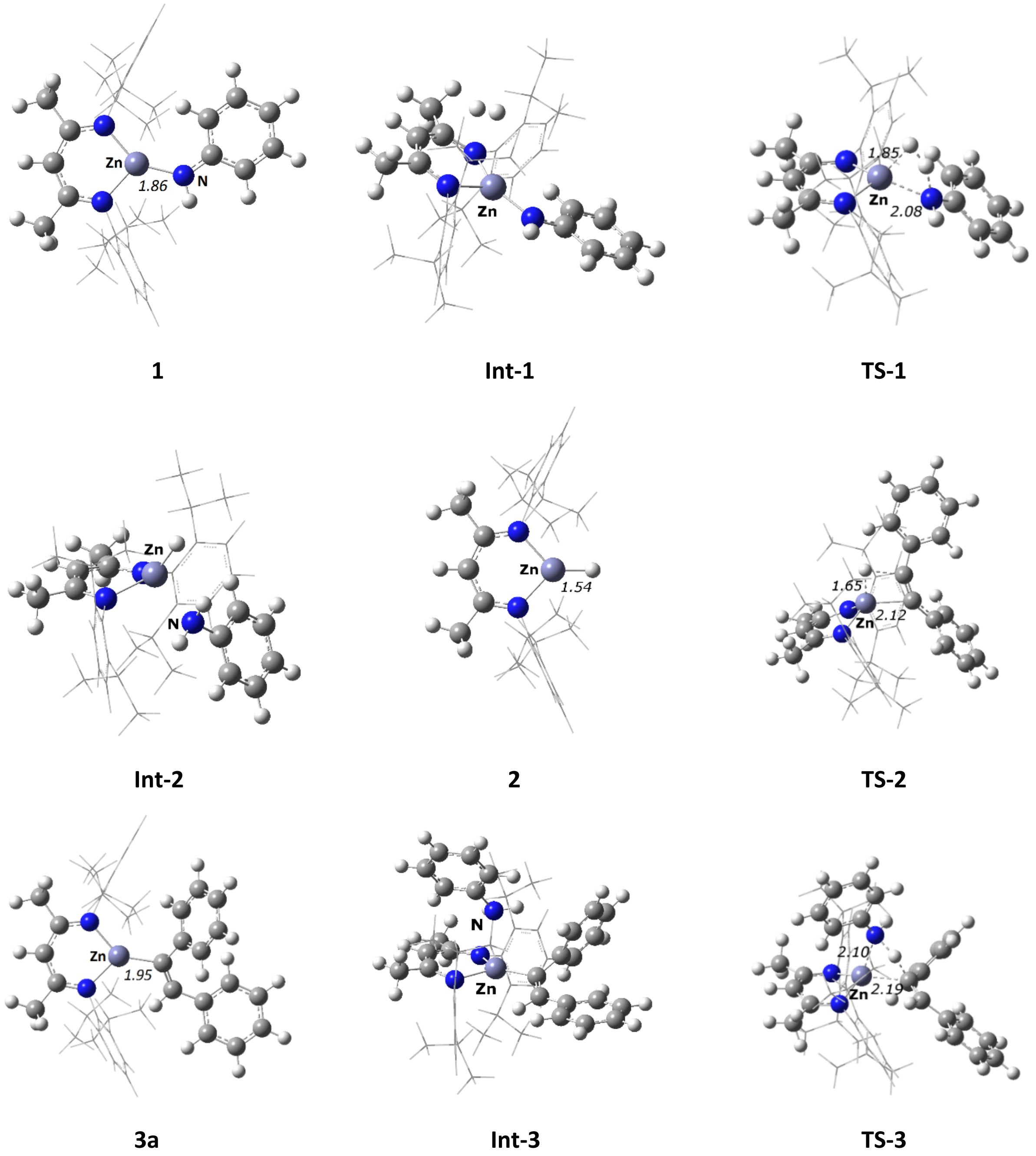

Figure S26. *Structures of* ***1***, ***Int-1***, ***TS-1***, ***Int-2***, ***2***, ***TS-2***, ***3a***, ***Int-3*** *and* ***TS-3*** *as calculated by DFT. Selected distances annotated, values in Å.*

Functional Testing

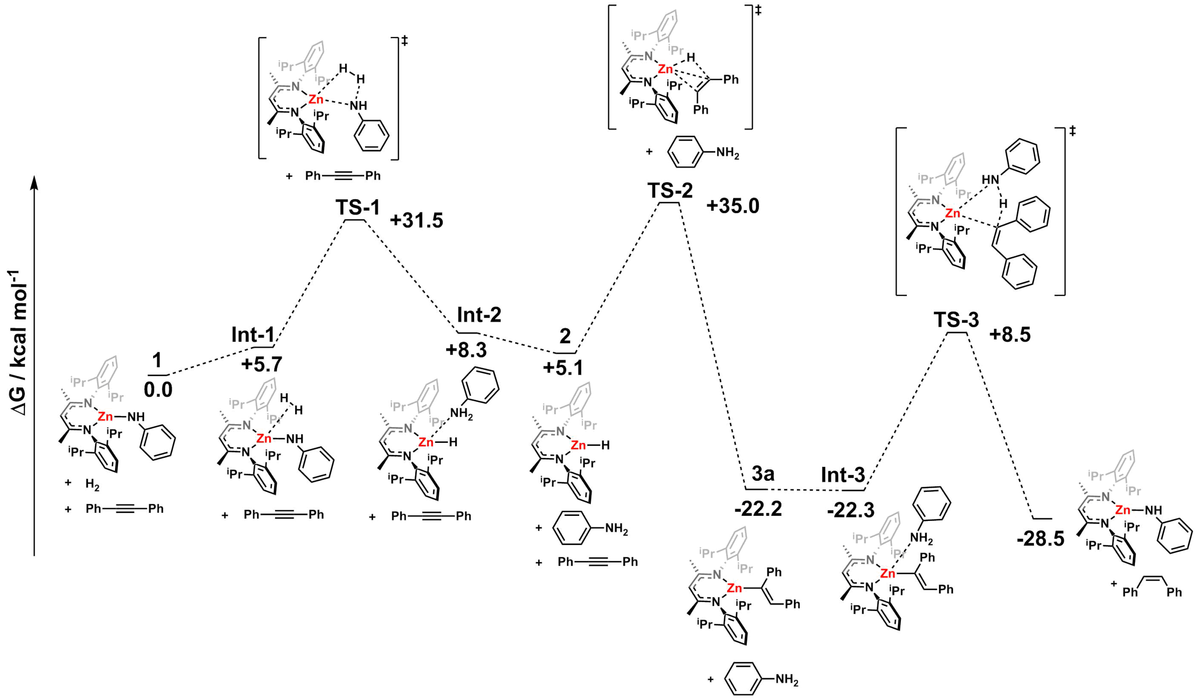

Figure S27. *Free energy reaction profile for the semi-hydrogenation of diphenylacetylene using **1**. Calculated using the M06L functional.*

Table S7. Single point corrected energies calculated with the M06-L functional with basis-set 1 (6-31G**/6-311+G*/SDD) and basis-set 2 (def2TVVPP)

	1	***Int-1***	***TS-1***	***Int-2***	***2***	***TS-2***	***3a***	***Int-3***	***TS-3***	***Prod***
BS1 (6-31G**/6-311+G*/SDD)	0.0	5.7	31.5	8.3	5.1	35.0	-22.2	-22.3	8.5	-28.5
BS2 (def2TZVPP)	0.0	4.6	28.2	9.4	4.4	33.9	-18.5	-17.8	12.6	-27.7

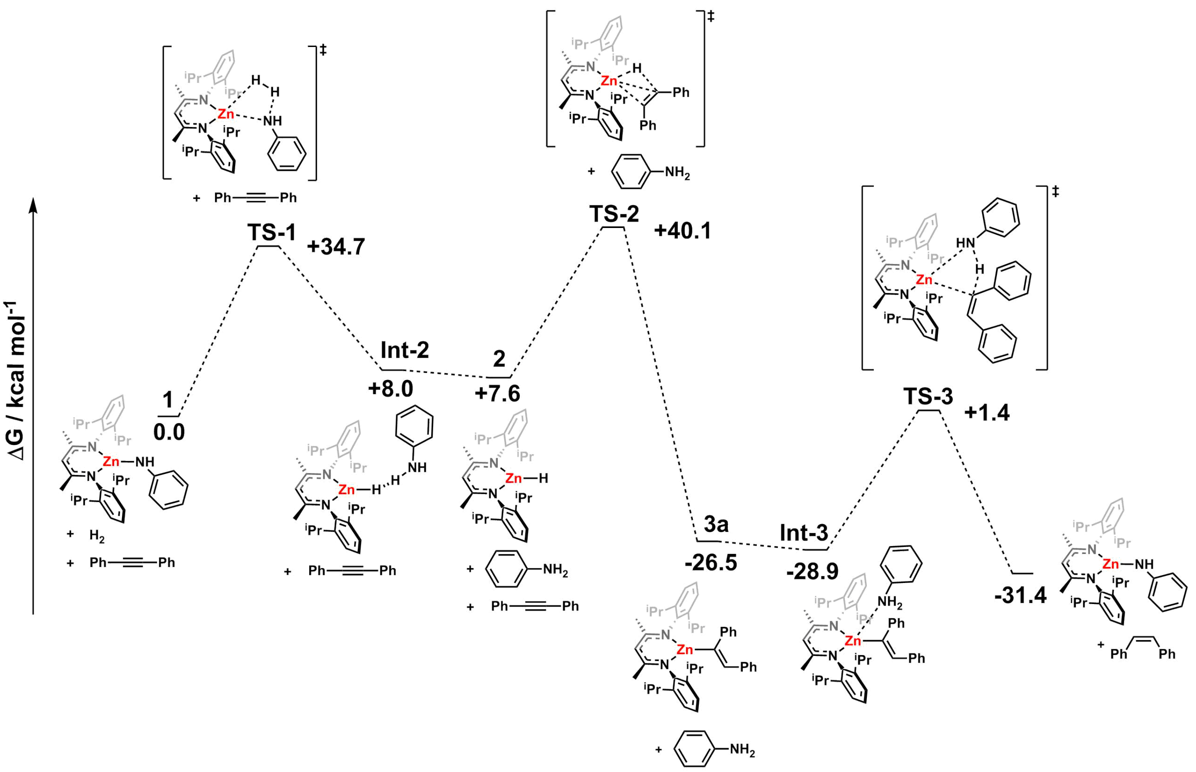

Figure S28. *Free energy reaction profile for the semi-hydrogenation of diphenylacetylene using* ***1****. Calculated using the B3PW91 functional. Int-1 was not found using this functional.*

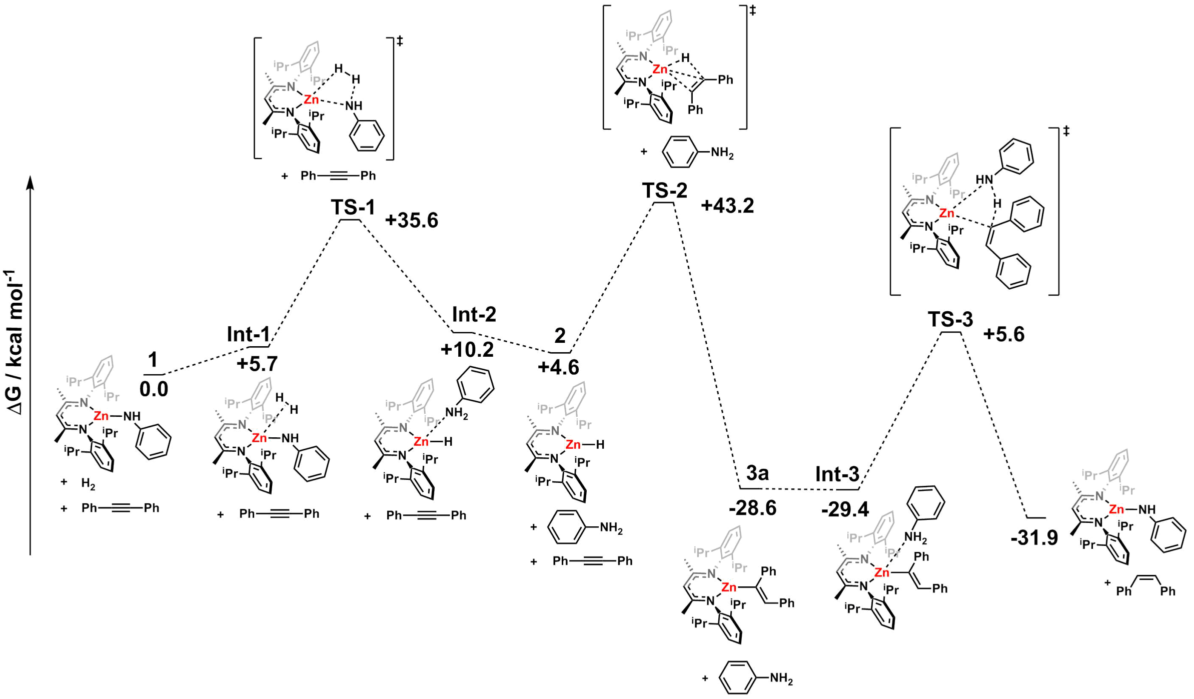

Figure S29. *Free energy reaction profile for the semi-hydrogenation of diphenylacetylene using* ***1****. Calculated using the ωB97X functional.*

NBO Analysis

Table S8. *Calculated NPA charges on key atoms in catalytic intermediates and transition states.*

Atom	***1***	***Int-1***	***TS-1***	***Int-2***	***2***	***TS-2***	***3a***	***Int-3***	***TS-3***
Zn	+1.58	+1.60	+1.57	+1.37	+1.32	+1.62	+1.48	+1.52	+1.61
N	-1.17	-1.17	-1.11	-0.97	-	-	-	-0.93	-1.10
H (1)	-	+0.01	-0.37	-0.56	-0.49	-0.39	+0.22	+0.22	+0.23
H (2)	-	-0.01	+0.24	+0.44	-	-	-	+0.43	+0.37
C (1)	-	-	-	-	-	-0.32	-0.56	-0.56	-0.56
C (2)	-	-	-	-	-	-0.09	-0.23	-0.24	-0.16

Table S9. *Calculated Wiberg bond indices of key bonds in catalytic intermediates and transition states.*

Bond	***1***	***Int-1***	***TS-1***	***Int-2***	***2***	***TS-2***	***3a***	***Int-3***	***TS-3***
Zn-N	0.31	0.30	0.16	0.06	-	-	-	0.05	0.14
Zn-H(1)	-	-	0.15	0.57	0.63	0.17	-	-	-
Zn-C(1)	-	-	-	-	-	0.08	0.45	0.40	0.14
N-H(2)	-	-	0.33	0.78	-	-	-	0.79	0.37
C(2)-H(1)	-	-	-	-	-	0.35	0.90	0.91	0.90
C(1)-H(2)	-	-	-	-	-	-	-	-	0.42
C(1)-C(2)	-	-	-	-	-	2.20	1.81	1.82	1.73
H-H	-	0.98	0.54	-	-	-	-	-	-

Donor-Acceptor Interactions

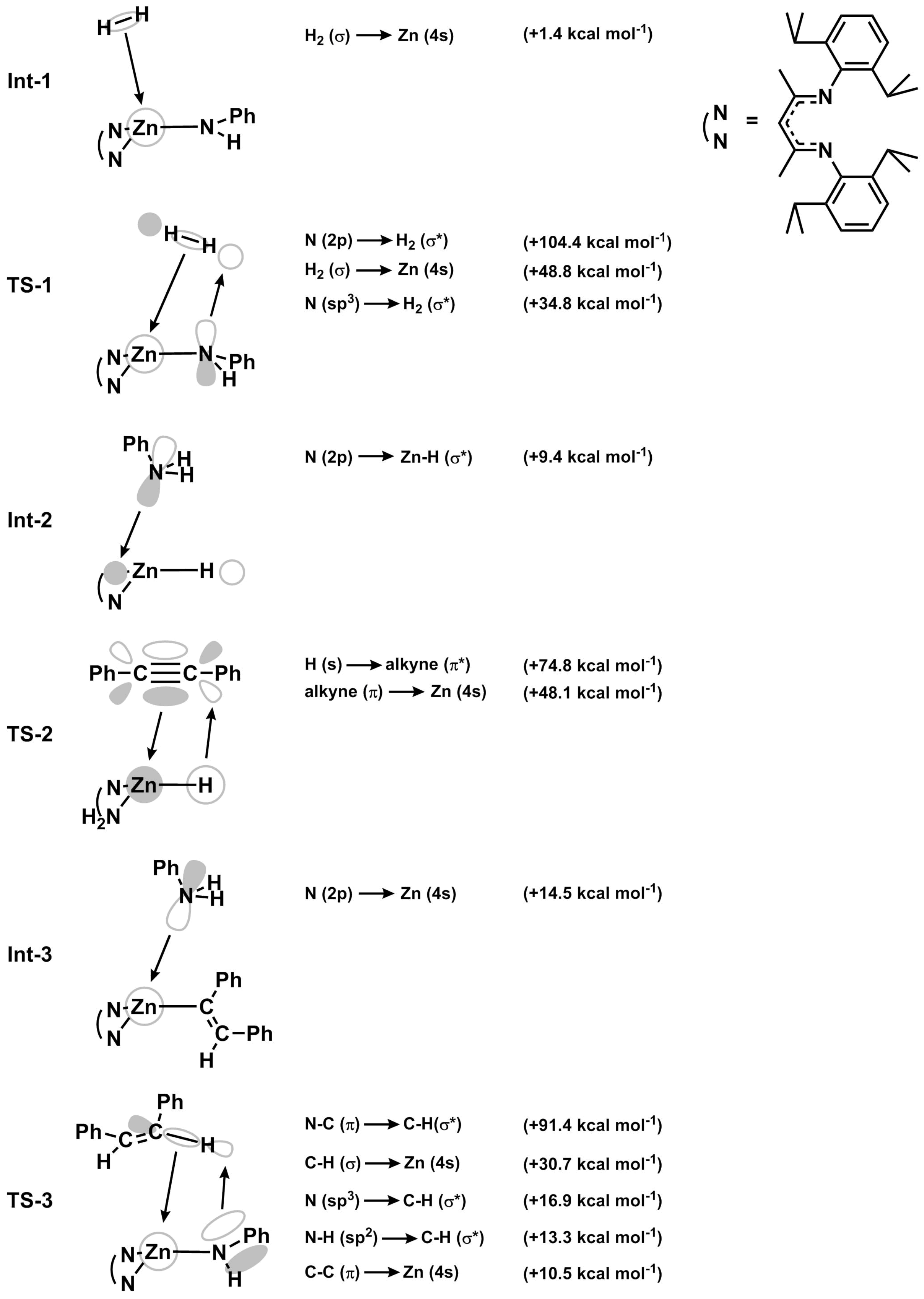

Figure S30. *Calculated donor-acceptor interactions in intermediates and transition states.*

Zn–N Rotation

Due to the dependence of H_2 activation on the planarity of compound **1**, as demonstrated by our NBO analysis, we investigated the energetic feasibility of a deviation from planarity. Calculations showed energy barrier to rotation about the Zn–N bond to the terminal anilide ligand to be relatively low, indicating loss of planarity is probable, however the planar orientations of **1** (where the N–Zn–N–H torsion angle is 0 or 180 °) both lie at energetic minima. These calculations support the idea that the planar geometry adopted by **1** for H_2 activation is the most favourable geometry, and while rotation about Zn–N is possible, there should be no energetic cost associated with a return to planarity.

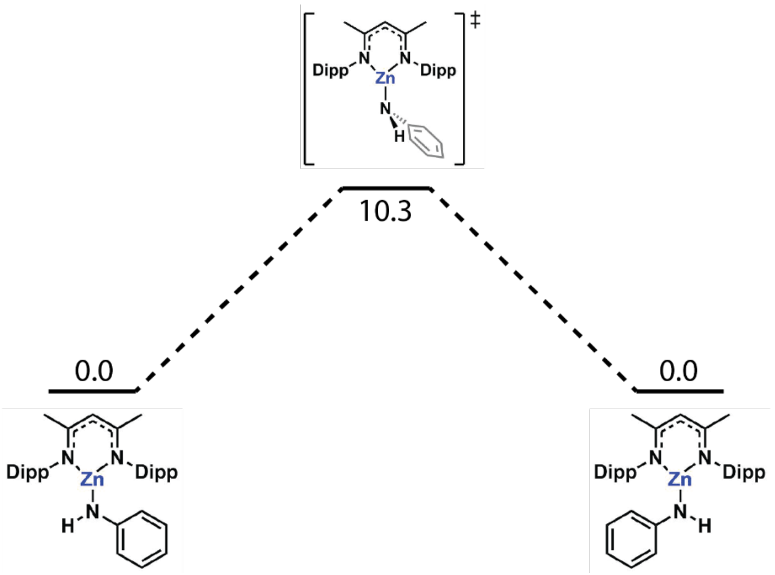

Figure S31. *Calculated energy profile for rotation about Zn–N bond. Values in kcal mol^{-1}.*

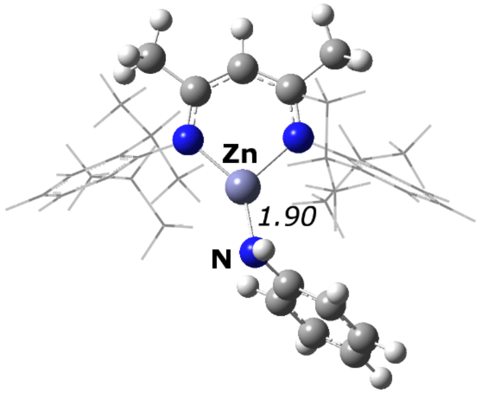

Figure S32. *Calculated transition state for rotation about Zn–N bond.*

5. XYZ Coordinates

Z-Matrix of 1

SCF (M06L) = -1753.45253038
E(SCF)+ZPE(0 K)= -1752.708662
H(298 K)= -1752.665242
G(298 K)= -1752.785042
Lowest Frequency = 13.5820 cm^{-1}

Zn	0.140626	-0.032565	0.181591
N	1.826516	-0.902875	-0.371205
C	3.185959	-2.612312	-1.465464
H	3.688381	-1.892465	-2.120141
H	3.070219	-3.554300	-2.002355
H	3.863886	-2.773308	-0.619956
N	-1.069954	-1.392920	-0.478055
C	1.861944	-2.070684	-1.003537
C	0.710844	-2.832041	-1.282671
H	0.889010	-3.779579	-1.778656
C	-0.638203	-2.516188	-1.063007
C	-1.665332	-3.504339	-1.539820
H	-2.326841	-3.806170	-0.720255
H	-1.197959	-4.394873	-1.960790
H	-2.313230	-3.056228	-2.301634
C	3.010381	-0.139646	-0.164059
C	3.284678	0.942492	-1.022690
C	4.411911	1.726453	-0.759040
H	4.640819	2.560520	-1.419988
C	5.236652	1.461114	0.324969
H	6.109278	2.080621	0.512012
C	4.936115	0.404363	1.178846
H	5.575977	0.211266	2.036057
C	3.823296	-0.407756	0.958604
C	2.401970	1.284699	-2.208709
H	1.554091	0.585098	-2.219174
C	1.834457	2.698997	-2.089034
H	2.634851	3.446866	-2.062420
H	1.234396	2.813409	-1.179934
H	1.195683	2.935472	-2.946176
C	3.153804	1.112152	-3.527862
H	4.007528	1.796825	-3.587087
H	2.500443	1.326322	-4.379897
H	3.540772	0.095212	-3.647397
C	3.438047	-1.498646	1.939027
H	2.926717	-2.291268	1.377682
C	4.629978	-2.134001	2.644153
H	5.386003	-2.483621	1.933667
H	4.306677	-2.990696	3.242743
H	5.118693	-1.434417	3.330721
C	2.435338	-0.953968	2.959277
H	2.893624	-0.163982	3.565023
H	2.092419	-1.742427	3.637632
H	1.553307	-0.516897	2.476207
C	-2.470064	-1.120767	-0.392850
C	-3.090004	-0.406885	-1.437566
C	-4.425938	-0.033384	-1.279072
H	-4.919166	0.528445	-2.068364
C	-5.130012	-0.358284	-0.125599
H	-6.167454	-0.054003	-0.018028
C	-4.504904	-1.070201	0.890946
H	-5.059669	-1.317212	1.792701
C	-3.169943	-1.465236	0.779241
C	-2.302639	0.013385	-2.663323
H	-1.478532	-0.700758	-2.795624
C	-3.131025	-0.002783	-3.943329
H	-3.903760	0.773265	-3.939557
H	-3.629473	-0.965691	-4.093582
H	-2.494473	0.188050	-4.812449
C	-1.678842	1.394363	-2.447911
H	-2.453830	2.152109	-2.287634
H	-1.080597	1.693624	-3.315732
H	-1.024239	1.429796	-1.566531
C	-2.465405	-2.169293	1.921034
H	-1.622422	-2.730352	1.496592
C	-3.356541	-3.164145	2.656039
H	-4.161381	-2.663633	3.204892
H	-2.774362	-3.727495	3.391377
H	-3.819349	-3.880574	1.969838
C	-1.888753	-1.139475	2.895726
H	-1.204744	-0.434550	2.406130
H	-1.338933	-1.627647	3.707533
H	-2.689598	-0.537793	3.340378
N	0.238067	1.616721	1.045626
H	1.160789	2.001446	1.204487
C	-0.765584	2.533704	1.275036
C	-2.118721	2.180174	1.105159
C	-0.487610	3.854176	1.682337
C	-3.135712	3.098082	1.324335
H	-2.362628	1.163525	0.798661
C	-1.512824	4.765545	1.902629
H	0.550390	4.154745	1.819265
C	-2.847125	4.402548	1.725866
H	-4.167352	2.783894	1.178899
H	-1.262319	5.776889	2.215174
H	-3.643976	5.119731	1.897312

Z-matrix of Int-1

SCF (M06L) = -1754.62957131
E(SCF)+ZPE(0 K)= -1753.871601
H(298 K)= -1753.826542
G(298 K)= -1753.947322
Lowest Frequency = 13.3113cm-1

Zn	-0.142763	0.058576	-0.226482
N	-1.849515	-0.906289	0.061528
C	-3.238816	-2.889452	0.421121
H	-3.795419	-2.405474	1.230418
H	-3.125163	-3.948390	0.655895
H	-3.865436	-2.800199	-0.472977
N	1.049134	-1.445899	0.057930
C	-1.904515	-2.224403	0.218016
C	-0.762617	-3.050501	0.227686
H	-0.958774	-4.109736	0.351345
C	0.595532	-2.693335	0.212909
C	1.597955	-3.794904	0.415958
H	2.164743	-3.634289	1.339793
H	2.336443	-3.812322	-0.392927
H	1.113527	-4.770095	0.471616
C	-3.025209	-0.109815	0.149450
C	-3.237922	0.645228	1.323196
C	-4.380840	1.445552	1.391615
H	-4.569827	2.029893	2.287801
C	-5.273992	1.517108	0.329252
H	-6.159641	2.141769	0.405131
C	-5.017487	0.811910	-0.840154
H	-5.700339	0.902121	-1.681197
C	-3.888639	-0.001222	-0.959564
C	-2.237705	0.596697	2.464161
H	-1.236156	0.537538	2.009890
C	-2.249578	1.851490	3.327016
H	-3.172104	1.938838	3.911717
H	-2.143629	2.758230	2.723672
H	-1.421863	1.826371	4.042025
C	-2.402954	-0.652918	3.329809
H	-3.403050	-0.689219	3.777088
H	-1.672017	-0.655422	4.146608
H	-2.254960	-1.572696	2.756307
C	-3.537902	-0.660260	-2.279214
H	-2.954686	-1.565283	-2.067100
C	-4.754150	-1.078886	-3.096501
H	-5.443045	-1.700103	-2.514901
H	-4.444283	-1.650404	-3.976336
H	-5.317911	-0.214159	-3.462626
C	-2.634230	0.271141	-3.091058
H	-3.162417	1.198266	-3.341512
H	-2.319063	-0.201112	-4.027967
H	-1.730412	0.551534	-2.534971
C	2.444784	-1.172136	0.194028
C	2.935122	-0.769587	1.451212
C	4.277038	-0.393837	1.547070
H	4.670781	-0.067212	2.506804
C	5.109576	-0.417357	0.435397
H	6.149923	-0.118218	0.528168
C	4.608054	-0.816920	-0.798390
H	5.262780	-0.824768	-1.666020
C	3.273222	-1.198560	-0.945038
C	2.014191	-0.649573	2.649731
H	1.123339	-1.262698	2.457189
C	2.644509	-1.153012	3.943976
H	3.484247	-0.525048	4.260342
H	3.016937	-2.177389	3.844842
H	1.910965	-1.137417	4.756282
C	1.551927	0.801706	2.805269
H	2.406666	1.462842	2.988177
H	0.857013	0.904590	3.646541
H	1.050441	1.183360	1.906012
C	2.700183	-1.551598	-2.302433
H	1.869135	-2.251513	-2.140768
C	3.703062	-2.235139	-3.224303
H	4.501856	-1.553816	-3.536236
H	3.207688	-2.581067	-4.136294
H	4.172934	-3.100153	-2.745239
C	2.120649	-0.303364	-2.971848
H	1.346612	0.179178	-2.360702
H	1.671649	-0.549107	-3.940606
H	2.901115	0.447847	-3.136630
N	-0.196146	1.897160	-0.537939
H	-1.102696	2.331436	-0.652181
C	0.842778	2.795888	-0.651841
C	2.179663	2.375704	-0.507933
C	0.619998	4.163906	-0.908703
C	3.233546	3.271185	-0.618490
H	2.382229	1.324486	-0.307423
C	1.681171	5.053929	-1.015815
H	-0.403423	4.518160	-1.023236
C	2.999234	4.622268	-0.873806
H	4.250710	2.902513	-0.501294
H	1.472202	6.102790	-1.214638
H	3.824413	5.322577	-0.959543
H	-0.579890	-2.156281	-2.289528
H	-0.467380	-1.544437	-2.707683

Z-matrix of TS-1

SCF (M06L) =	-1754.58762318
E(SCF)+ZPE(0 K)=	-1753.830651
H(298 K)=	-1753.786663
G(298 K)=	-1753.907118
Lowest Frequency =	-1464.4693 cm^{-1}

Zn	-0.246475	-0.343983	-0.532316
N	-1.910321	-0.675794	0.481948
C	-3.256462	-1.870513	2.136358
H	-3.770240	-0.945643	2.416542
H	-3.117186	-2.482595	3.028265
H	-3.933208	-2.403594	1.458752
N	0.999876	-1.318836	0.619764
C	-1.946498	-1.580889	1.455862
C	-0.817392	-2.292541	1.904901
H	-1.014359	-3.019144	2.685266
C	0.538540	-2.139376	1.567187
C	1.532112	-2.943396	2.360417
H	2.134589	-2.292791	3.004612
H	2.239921	-3.456171	1.701452
H	1.034164	-3.680317	2.991309
C	-3.044472	0.121977	0.163849
C	-3.061248	1.460242	0.621846
C	-4.140373	2.269581	0.261957
H	-4.173418	3.298651	0.610035
C	-5.168806	1.784430	-0.538780
H	-5.999765	2.430186	-0.808766
C	-5.121046	0.477724	-1.003944
H	-5.913926	0.107138	-1.650616
C	-4.065237	-0.374725	-0.669659
C	-1.936826	1.992270	1.490851
H	-1.008378	1.520256	1.141542
C	-1.741907	3.498747	1.372310
H	-2.553256	4.057628	1.852358
H	-1.688202	3.822306	0.327721
H	-0.809572	3.800237	1.859580
C	-2.108830	1.590161	2.956506
H	-3.055089	1.971049	3.358217
H	-1.297171	2.001139	3.567068
H	-2.098520	0.504160	3.087711
C	-4.012384	-1.769808	-1.259661
H	-3.221952	-2.328645	-0.744253
C	-5.323781	-2.529762	-1.074407
H	-5.637862	-2.559344	-0.025701
H	-5.226320	-3.560951	-1.427638
H	-6.138813	-2.070316	-1.644175
C	-3.631754	-1.705259	-2.739487
H	-4.380644	-1.142160	-3.308383
H	-3.569700	-2.709147	-3.172337
H	-2.663263	-1.215619	-2.883249
C	2.404334	-1.129534	0.455183
C	3.079114	-0.217636	1.296362
C	4.432002	0.024891	1.057443
H	4.964106	0.733432	1.687408
C	5.108403	-0.612378	0.022610
H	6.162514	-0.409150	-0.145652
C	4.429917	-1.504987	-0.795038
H	4.957610	-2.004721	-1.605429
C	3.072705	-1.778693	-0.600334
C	4.432002	0.024891	1.057443
H	4.964106	0.733432	1.687408
C	5.108403	-0.612378	0.022610
H	6.162514	-0.409150	-0.145652
C	4.429917	-1.504987	-0.795038
H	4.957610	-2.004721	-1.605429
C	3.072705	-1.778693	-0.600334
C	2.340796	0.544876	2.380915
H	1.471230	-0.051110	2.689407
C	3.186960	0.792308	3.625205
H	4.003502	1.495872	3.430953
H	3.631292	-0.131819	4.008827
H	2.575233	1.229086	4.420364
C	1.802341	1.863177	1.822771
H	2.616967	2.503736	1.465202
H	1.244940	2.414077	2.589209
H	1.133882	1.695960	0.971385
C	2.369051	-2.750833	-1.525950
H	1.307585	-2.772134	-1.250365
C	2.923233	-4.165935	-1.363803
H	3.985551	-4.205133	-1.631105
H	2.393516	-4.870391	-2.013037
H	2.831983	-4.524976	-0.333603
C	2.461303	-2.300760	-2.982779
H	2.051320	-1.295488	-3.121273
H	1.901887	-2.979827	-3.634026
H	3.500133	-2.290068	-3.331852
N	-0.143472	1.490776	-1.503711
H	-1.005085	2.031031	-1.488314
C	0.980592	2.315084	-1.626417
C	2.262094	1.739824	-1.670533
C	0.879888	3.712845	-1.693517
C	3.394726	2.535421	-1.777034
H	2.364642	0.657216	-1.601831
C	2.019984	4.504418	-1.789265
H	-0.105596	4.174841	-1.663937
C	3.285675	3.925011	-1.831246
H	4.372414	2.060437	-1.803003
H	1.914494	5.585716	-1.831132
H	4.173721	4.545615	-1.903361
H	-0.326169	-0.660972	-2.355540
H	-0.248367	0.353163	-2.255230

Z-matrix of Int-2
SCF (M06L) = -1754.63150248
E(SCF)+ZPE(0 K)= -1753.868655
H(298 K)= -1753.824429
G(298 K)= -1753.943335
Lowest Frequency = 24.5522cm-1

Zn	-0.346280	-0.945850	-0.774294
N	-1.831315	-0.650679	0.562997
C	-3.108911	-1.457494	2.489243
H	-3.534806	-0.471220	2.701669
H	-2.952355	-1.981306	3.433186
H	-3.867589	-1.999183	1.912596
N	1.031474	-1.160224	0.717372
C	-1.831589	-1.338931	1.702599
C	-0.683455	-1.947691	2.235428
H	-0.830481	-2.511094	3.150301
C	0.657313	-1.766107	1.841614
C	1.708545	-2.266381	2.795510
H	2.138094	-1.436742	3.368320
H	2.540888	-2.738086	2.264660
H	1.286648	-2.977105	3.507826
C	-2.955787	0.131140	0.196602
C	-2.930398	1.517416	0.487678
C	-4.016897	2.299467	0.087026
H	-4.020877	3.362989	0.309990
C	-5.092651	1.745325	-0.599843
H	-5.927128	2.372194	-0.901834
C	-5.085585	0.392855	-0.908903
H	-5.915812	-0.037561	-1.465979
C	-4.027867	-0.435057	-0.521766
C	-1.732747	2.138709	1.188747
H	-0.835786	1.652899	0.770998
C	-1.619778	3.640883	0.948601
H	-2.376874	4.196733	1.513634
H	-1.745409	3.905743	-0.106397
H	-0.640179	4.006741	1.270752
C	-1.713762	1.871900	2.696251
H	-2.632680	2.240216	3.167611
H	-0.869949	2.394040	3.162685
H	-1.607183	0.810864	2.933497
C	-4.036302	-1.895683	-0.925231
H	-3.211899	-2.395901	-0.402278
C	-5.337090	-2.596761	-0.539371
H	-5.562298	-2.488836	0.526729
H	-5.282101	-3.665967	-0.766163
H	-6.191185	-2.192534	-1.094147
C	-3.779578	-2.033338	-2.426318
H	-4.569578	-1.537011	-3.002507
H	-3.757737	-3.086111	-2.726129
H	-2.819344	-1.589921	-2.708816
C	2.404277	-0.854152	0.506308
C	2.984383	0.260124	1.153325
C	4.307159	0.593932	0.856754
H	4.752075	1.461393	1.341643
C	5.057532	-0.148754	-0.045973
H	6.086678	0.126310	-0.261436
C	4.481004	-1.250784	-0.664437
H	5.069110	-1.849508	-1.358240
C	3.160377	-1.625033	-0.403532
C	2.209226	1.132537	2.122817
H	1.239479	0.657964	2.318149
C	2.930374	1.297951	3.459759
H	3.863808	1.860124	3.343801
H	3.186873	0.336716	3.916471
H	2.305095	1.851215	4.168549
C	1.933961	2.501976	1.505116
H	2.870282	3.024915	1.276733
H	1.359403	3.134533	2.192386
H	1.375878	2.415910	0.568280
C	2.605130	-2.872759	-1.062626
H	1.546815	-2.959360	-0.792848
C	3.323304	-4.120219	-0.546651
H	4.390173	-4.093942	-0.797753
H	2.900606	-5.026838	-0.991716
H	3.245976	-4.212922	0.541459
C	2.684860	-2.800470	-2.584986
H	2.126646	-1.943535	-2.974875
H	2.255456	-3.700195	-3.037035
H	3.721842	-2.718823	-2.931217
N	-0.536967	1.143329	-1.881043
H	-1.344579	1.711594	-1.641395
C	0.656656	1.918665	-1.994950
C	1.873322	1.255253	-2.177376
C	0.642063	3.308245	-1.867837
C	3.060289	1.976474	-2.221704
H	1.883714	0.169262	-2.259876
C	1.836531	4.024074	-1.899948
H	-0.305294	3.826611	-1.740997
C	3.048502	3.362806	-2.075619
H	3.998203	1.442803	-2.345803
H	1.813855	5.104896	-1.791592
H	3.978688	3.922697	-2.097410
H	-0.316279	-1.738022	-2.126543
H	-0.747305	0.650196	-2.743240

Z-matrix of 2

SCF (M06L) = -1467.02577469
E(SCF)+ZPE(0 K)= -1466.381855
H(298 K)= -1466.344712
G(298 K)= -1466.449149
Lowest Frequency = 19.3278 cm^{-1}

N	-1.459773	0.046227	0.536923
C	-2.462128	0.138987	2.764423
H	-3.068197	1.021716	2.533672
H	-2.166323	0.180788	3.813214
H	-3.121593	-0.724381	2.622210
N	1.460361	-0.014270	0.536758
C	-1.265759	0.065751	1.855940
C	0.000634	0.022464	2.460551
H	0.000738	0.024716	3.544850
C	1.266688	-0.030760	1.855787
C	2.462241	-0.116578	2.764289
H	3.053393	-1.010916	2.539503
H	2.166282	-0.146675	3.813438
H	3.136018	0.734534	2.615867
C	-2.787670	0.035511	0.016195
C	-3.376528	1.241582	-0.406644
C	-4.642245	1.190403	-0.996782
H	-5.106209	2.114115	-1.336715
C	-5.312673	-0.014364	-1.156279
H	-6.296923	-0.034623	-1.615911
C	-4.721381	-1.196873	-0.724156
H	-5.252868	-2.136811	-0.849029
C	-3.456415	-1.198057	-0.135407
C	-2.653091	2.567049	-0.271250
H	-1.802793	2.418222	0.407426
C	-2.094456	3.009856	-1.624456
H	-2.905851	3.162800	-2.345373
H	-1.419025	2.259261	-2.050375
H	-1.543934	3.952586	-1.535601
C	-3.537286	3.656920	0.328984
H	-4.370911	3.912665	-0.334078
H	-2.962426	4.574342	0.489518
H	-3.964135	3.353310	1.289978
C	-2.787619	-2.491332	0.291285
H	-2.146858	-2.267876	1.154764
C	-3.773604	-3.569734	0.724429
H	-4.472332	-3.202283	1.482825
H	-3.240160	-4.428191	1.143234
H	-4.365455	-3.943904	-0.117911
C	-1.875237	-3.018545	-0.817672
H	-2.456625	-3.266789	-1.712511
H	-1.344084	-3.921136	-0.496361
H	-1.123977	-2.278549	-1.119193
C	2.787802	-0.035978	0.015178
C	3.486682	1.180179	-0.140491
C	4.751422	1.145878	-0.728609
H	5.305746	2.072121	-0.856464
C	5.313590	-0.052247	-1.156595
H	6.298330	-0.057808	-1.615586
C	4.613100	-1.239450	-0.994606
H	5.053757	-2.175256	-1.332520
C	3.346145	-1.257443	-0.405244
C	2.848241	2.490910	0.279043
H	2.201928	2.287021	1.143132
C	3.858627	3.548500	0.707062
H	4.460191	3.903522	-0.136677
H	4.547555	3.169516	1.468768
H	3.344903	4.421801	1.119782
C	1.949278	3.032092	-0.834176
H	2.538004	3.264183	-1.728549
H	1.436228	3.946919	-0.518424
H	1.184402	2.305684	-1.134109
C	2.588929	-2.564153	-0.270712
H	1.737068	-2.392496	0.400741
C	3.441240	-3.673460	0.339998
H	4.275172	-3.949734	-0.314376
H	2.843392	-4.576842	0.496298
H	3.865508	-3.378199	1.304715
C	2.030489	-2.997346	-1.627154
H	1.379402	-2.230328	-2.061909
H	1.453239	-3.924050	-1.539738
H	2.843629	-3.174942	-2.340417
H	0.000786	0.028888	-2.340804
Zn	0.000227	0.022360	-0.804822

Z-matrix of TS-2

SCF (M06L) =	-2006.40425808
E(SCF)+ZPE(0 K)=	-2005.568359
H(298 K)=	-2005.519906
G(298 K)=	-2005.647437
Lowest Frequency =	-644.6456 cm^{-1}

N	-2.065859	-0.052400	-0.909022
C	-3.702190	-0.641716	-2.623453
H	-4.195021	0.336533	-2.648662
H	-4.290997	-1.260637	-1.938322
H	-3.748733	-1.081007	-3.620561
N	0.724304	-0.706429	-1.675704
C	-2.282432	-0.507830	-2.144674
C	-1.266693	-0.915747	-3.026973
H	-1.609358	-1.255881	-3.998642
C	0.098136	-1.155401	-2.756517
C	0.830758	-2.005726	-3.759376
H	0.616971	-3.066960	-3.591335
H	1.913964	-1.873788	-3.703378
H	0.492617	-1.771772	-4.771952
C	-3.149953	0.144339	-0.008619
C	-3.429252	-0.867541	0.939433
C	-4.492044	-0.668071	1.822876
H	-4.730569	-1.437172	2.552200
C	-5.244778	0.501348	1.796329
H	-6.068176	0.634339	2.492759
C	-4.924218	1.504733	0.893761
H	-5.490922	2.434080	0.894969
C	-3.876185	1.351032	-0.017753
C	-2.575253	-2.122218	1.007723
H	-1.529307	-1.796777	0.879853
C	-2.678285	-2.846850	2.345563
H	-3.643618	-3.355465	2.453131
H	-2.563942	-2.170058	3.197241
H	-1.897827	-3.610667	2.420484
C	-2.882555	-3.114558	-0.118091
H	-3.942516	-3.395561	-0.106242
H	-2.297674	-4.032330	0.014216
H	-2.644339	-2.720309	-1.108856
C	-3.508080	2.500921	-0.934194
H	-2.765714	2.137391	-1.655732
C	-4.706809	3.036444	-1.714231
H	-5.210855	2.251804	-2.287884
H	-4.395417	3.817625	-2.414788
H	-5.453555	3.480285	-1.046429
C	-2.855386	3.625414	-0.127680
H	-3.563998	4.045066	0.596104
H	-2.525662	4.438839	-0.783376
H	-1.982920	3.267900	0.428188
C	1.997543	-1.198187	-1.284645
C	2.132229	-2.512511	-0.784759
C	3.389854	-2.929933	-0.339740
H	3.498091	-3.939104	0.054671
C	4.487521	-2.082629	-0.368383
H	5.456301	-2.427863	-0.017224
C	4.336832	-0.782539	-0.840195
H	5.195087	-0.116340	-0.853746
C	3.103330	-0.315220	-1.297045
C	0.963915	-3.472132	-0.650906
H	0.071861	-3.005160	-1.087100
C	1.217086	-4.789406	-1.384574
H	2.024423	-5.357630	-0.908818
H	1.503270	-4.636643	-2.429624
H	0.322125	-5.420496	-1.365690
C	0.661900	-3.751660	0.821898
H	1.523385	-4.212794	1.318888
H	-0.183669	-4.442571	0.920188
H	0.423014	-2.837422	1.373985
C	2.959416	1.090371	-1.845993
H	1.969874	1.463489	-1.548416
C	2.984514	1.082148	-3.375508
H	3.935732	0.682600	-3.746357
H	2.868910	2.096025	-3.772765
H	2.178171	0.469462	-3.789274
C	3.993884	2.066479	-1.298971
H	4.038660	2.041792	-0.204594
H	3.744178	3.089739	-1.595832
H	4.998930	1.855607	-1.682262
H	-0.019451	2.203080	-0.627558
Zn	-0.255298	0.581590	-0.486194
C	0.985360	-0.346646	2.379888
C	2.284798	-0.888893	2.374512
H	3.013001	-0.512932	1.658095
C	2.617697	-1.914597	3.249648
H	3.621373	-2.332024	3.218348
C	1.678492	-2.410276	4.153001
H	1.944530	-3.212019	4.835649
C	0.391778	-1.874298	4.166770
H	-0.350786	-2.256792	4.862595
C	0.039092	-0.861959	3.281996
H	-0.969316	-0.454890	3.277751
C	0.636827	0.671072	1.430709
C	1.483092	3.108170	0.999805
C	1.306978	4.153475	0.089663
H	0.611148	4.011411	-0.734699
C	2.019449	5.338390	0.233288
H	1.873740	6.141852	-0.483250
C	2.917557	5.495850	1.285486
H	3.476634	6.420854	1.393112

C	3.095036	4.460006	2.202177
H	3.793258	4.574179	3.026567
C	2.385074	3.275319	2.066874
H	2.524518	2.461444	2.773936
C	0.824362	1.821328	0.920771

Z-matrix of 3a

SCF (M06L) =	-2006.49837637
E(SCF)+ZPE(0 K)=	-2005.656310
H(298 K)=	-2005.607501
G(298 K)=	-2005.737962
Lowest Frequency =	14.2085 cm^{-1}

Zn	-0.251218	-0.462098	-0.032892
N	0.725239	-2.148040	0.404810
C	0.139293	-3.315677	0.653997
C	-1.249316	-3.518349	0.574650
H	-1.589105	-4.526085	0.786224
C	-2.256461	-2.593220	0.259709
N	-2.031363	-1.304059	-0.002535
C	0.993640	-4.487639	1.050536
H	1.732266	-4.720229	0.275412
H	0.393399	-5.378661	1.238009
H	1.567601	-4.254427	1.954020
C	-3.672026	-3.098163	0.216655
H	-4.304055	-2.564692	0.935467
H	-3.723205	-4.165729	0.432988
H	-4.116474	-2.916958	-0.768092
C	2.142037	-2.013737	0.457952
C	2.901045	-2.306539	-0.696939
C	4.266825	-2.021396	-0.682296
H	4.865990	-2.234325	-1.563896
C	4.872738	-1.461290	0.438563
H	5.936417	-1.239076	0.428388
C	4.114307	-1.192776	1.569902
H	4.589370	-0.757037	2.447072
C	2.744167	-1.466690	1.606536
C	2.226450	-2.849856	-1.942569
H	1.373520	-3.462576	-1.622021
C	1.661963	-1.709137	-2.790998
H	0.949303	-1.092804	-2.229101
H	1.141322	-2.094450	-3.674464
H	2.462433	-1.041920	-3.130966
C	3.135324	-3.739160	-2.782863
H	3.944474	-3.169246	-3.252072
H	2.567632	-4.208191	-3.591912
H	3.592923	-4.533934	-2.185125
C	1.950245	-1.153217	2.859877
H	0.928180	-1.527941	2.715094
C	2.533661	-1.855598	4.084770
H	2.604453	-2.938379	3.940571
H	1.914548	-1.672170	4.968606
H	3.541167	-1.489958	4.312345
C	1.862996	0.354996	3.092414
H	2.862152	0.791435	3.211042
H	1.288891	0.581717	3.997241
H	1.385343	0.861639	2.247997
C	-3.122556	-0.428726	-0.281631
C	-3.769947	0.225369	0.787810
C	-4.753400	1.167804	0.485830
H	-5.256575	1.690789	1.295170
C	-5.090993	1.460322	-0.831358
H	-5.855300	2.202798	-1.044971
C	-4.449953	0.800764	-1.871633
H	-4.714443	1.032878	-2.901254
C	-3.461869	-0.155552	-1.620336
C	-2.735553	-0.819361	-2.774180
H	-2.180720	-1.678637	-2.374824
C	-1.717493	0.147066	-3.382842
H	-2.218779	1.039697	-3.775693
H	-1.168903	-0.320422	-4.207430
H	-0.982614	0.488925	-2.642604
C	-3.689257	-1.341420	-3.845157
H	-4.441251	-2.017519	-3.426563
H	-3.138615	-1.885722	-4.618589
H	-4.222770	-0.525983	-4.345448
C	0.551619	1.304797	-0.262857
C	1.854279	1.354501	-0.640267
H	2.371184	0.403347	-0.798055
C	-0.227579	2.482974	0.165803
C	0.255392	3.335816	1.174888
H	1.251966	3.161708	1.575825
C	-0.515601	4.386634	1.657550
H	-0.120503	5.025818	2.443271
C	-1.788171	4.623969	1.138712
H	-2.389534	5.446835	1.515416
C	-2.282146	3.790613	0.137657
H	-3.275847	3.953614	-0.272581
C	-1.517983	2.725994	-0.330444
H	-1.927305	2.065347	-1.093844
C	2.735787	2.506854	-0.859157
C	2.283735	3.771881	-1.271212
H	1.223164	3.929482	-1.443850
C	3.179342	4.813545	-1.475819
H	2.809312	5.782165	-1.801760
C	4.546053	4.622613	-1.273745
H	5.242259	5.441489	-1.433251
C	5.012402	3.370573	-0.880468
H	6.076728	3.207351	-0.731315
C	4.118109	2.324101	-0.687323
H	4.479158	1.340044	-0.389224

C	-3.366402	-0.054602	2.223391
H	-3.030961	-1.099260	2.277711
C	-4.516312	0.105587	3.211511
H	-5.399099	-0.465887	2.906930
H	-4.215254	-0.239995	4.204863
H	-4.819033	1.152755	3.318493
C	-2.182572	0.821212	2.639298
H	-1.300226	0.649622	2.011104
H	-2.428963	1.885764	2.551035
H	-1.891141	0.619342	3.675948

Z-matrix of Int-3

SCF (M06L) = -2294.10865814
E(SCF)+ZPE(0 K)= -2293.147372
H(298 K)= -2293.091424
G(298 K)= -2293.237185
Lowest Frequency = 17.4208cm-1

Zn	-0.163320	0.067089	0.335813
N	0.939116	1.452615	1.229934
C	0.442464	2.656087	1.488481
C	-0.937314	2.956820	1.472382
H	-1.184961	3.973390	1.759398
C	-2.039213	2.095906	1.317511
N	-1.951271	0.848862	0.850514
C	1.381594	3.772483	1.852859
H	2.076455	3.467425	2.641675
H	0.844062	4.664193	2.178422
H	1.997118	4.038590	0.984625
C	-3.384435	2.625386	1.733039
H	-4.106853	2.583229	0.911179
H	-3.319334	3.654488	2.087657
H	-3.803051	2.006967	2.534654
C	2.304199	1.142024	1.506458
C	2.596160	0.445181	2.703402
C	3.923346	0.102588	2.966033
H	4.165976	-0.429306	3.881404
C	4.940027	0.410358	2.068322
H	5.967154	0.136072	2.292800
C	4.628410	1.040928	0.873270
H	5.414019	1.244324	0.147706
C	3.315278	1.408023	0.564518
C	1.482058	0.044599	3.652289
H	0.631703	-0.260112	3.021750
C	1.839580	-1.146775	4.531926
H	2.178781	-2.003859	3.941127
H	0.966869	-1.460346	5.114054
H	2.625873	-0.900887	5.254258
C	1.007026	1.209126	4.523265
H	1.830501	1.592607	5.137071
H	0.210732	0.883063	5.201906
H	0.611440	2.039182	3.932276
C	3.010901	1.978438	-0.803189
H	1.961882	2.294451	-0.815837
C	3.867133	3.193628	-1.149514
H	3.782013	3.984591	-0.396337
H	3.558239	3.612661	-2.112826
H	4.928605	2.932018	-1.228804
C	3.179474	0.882528	-1.858268
H	4.194246	0.467601	-1.829750
H	3.006636	1.278875	-2.863493
H	2.480650	0.055057	-1.686469
C	-3.085425	-0.018139	0.878952
C	-4.039971	-0.003979	-0.161362
C	-5.055968	-0.961991	-0.152713
H	-5.781488	-0.966617	-0.964127
C	-5.149937	-1.910984	0.856254
H	-5.945307	-2.650989	0.841244
C	-4.220182	-1.900565	1.887371
H	-4.290715	-2.637406	2.685556
C	-3.184412	-0.964069	1.922690
C	-2.184198	-1.016816	3.060990
H	-1.517603	-0.149508	2.966217
C	-1.331905	-2.283140	2.967973
H	-1.962314	-3.179678	3.000294
H	-0.624309	-2.346330	3.801008
H	-0.754902	-2.317240	2.035869
C	-2.862718	-0.921962	4.425983
H	-3.472206	-0.017473	4.516621
H	-2.115272	-0.906770	5.226548
H	-3.517926	-1.780487	4.611010
C	0.391622	-1.644961	-0.421220
C	1.633539	-2.113948	-0.151143
H	2.240257	-1.541696	0.558661
C	-0.507060	-2.194250	-1.452113
C	-0.170167	-2.116192	-2.816954
H	0.808641	-1.722452	-3.090718
C	-1.051012	-2.557000	-3.801407
H	-0.765406	-2.487363	-4.848262
C	-2.286665	-3.097943	-3.449809
H	-2.971383	-3.446301	-4.217923
C	-2.639316	-3.174299	-2.103359
H	-3.606457	-3.578695	-1.813614
C	-1.771416	-2.711490	-1.119328
H	-2.068256	-2.750992	-0.071537
C	2.358768	-3.263942	-0.701193
C	1.744153	-4.385742	-1.283391
H	0.660591	-4.443746	-1.325457
C	2.508174	-5.431665	-1.786408
H	2.012358	-6.293452	-2.225726
C	3.900325	-5.387103	-1.724909

H	4.492101	-6.207618	-2.121386
C	4.525432	-4.288450	-1.139452
H	5.609809	-4.246869	-1.076869
C	3.762169	-3.246681	-0.627241
H	4.246818	-2.388041	-0.163015
C	-3.976824	0.974817	-1.317230
H	-3.227947	1.741931	-1.073245
C	-5.299441	1.707319	-1.536603
H	-5.643605	2.215395	-0.630046
H	-5.199941	2.457053	-2.327604
H	-6.091742	1.016231	-1.842830
C	-3.545893	0.262725	-2.601088
H	-2.640598	-0.336549	-2.456202
H	-4.324261	-0.435396	-2.929582
H	-3.370269	0.976160	-3.414188
C	1.286727	3.371822	-4.392011
C	0.711710	2.265777	-3.773557
C	0.035831	2.410710	-2.557952
C	-0.067223	3.683171	-1.980821
C	0.506338	4.782267	-2.609074
C	1.189467	4.636038	-3.815730
H	1.809956	3.241160	-5.335464
H	0.791799	1.278046	-4.224315
H	-0.583226	3.791051	-1.027360
H	0.420841	5.762552	-2.147409
H	1.637772	5.497073	-4.301748
N	-0.487599	1.288649	-1.884330
H	-1.390692	1.481938	-1.465762
H	-0.573240	0.475263	-2.485855

Z-matrix of TS-3

SCF (M06L) =	-2294.05712252
E(SCF)+ZPE(0 K)=	-2293.101342
H(298 K)=	-2293.046383
G(298 K)=	-2293.188378
Lowest Frequency =	-1363.1825 cm^{-1}

Zn	-0.264062	0.472420	-0.014057
N	0.966448	1.052696	1.459602
C	0.468771	1.792987	2.446117
C	-0.905754	2.069981	2.596806
H	-1.146646	2.784394	3.376308
C	-2.018032	1.427021	2.019921
N	-1.949582	0.502368	1.057513
C	1.399603	2.357561	3.484156
H	1.988605	1.558520	3.947447
H	0.853731	2.887482	4.265484
H	2.122944	3.046055	3.033765
C	-3.373055	1.828231	2.541566
H	-3.946183	2.316217	1.744560
H	-3.291696	2.519727	3.381053
H	-3.965046	0.962919	2.852317
C	2.331120	0.643701	1.476944
C	2.623083	-0.690572	1.845599
C	3.953316	-1.111040	1.830527
H	4.191494	-2.135075	2.105165
C	4.976264	-0.252516	1.442825
H	6.006312	-0.599472	1.437234
C	4.669605	1.037012	1.032093
H	5.463593	1.697628	0.689250
C	3.352432	1.506336	1.032006
C	1.512221	-1.647040	2.229908
H	0.655185	-1.411909	1.578613
C	1.859380	-3.112011	1.992933
H	2.251718	-3.282015	0.984940
H	0.966143	-3.732997	2.116121
H	2.603220	-3.477199	2.710324
C	1.060199	-1.432480	3.675477
H	1.904943	-1.548956	4.364385
H	0.299034	-2.169257	3.957288
H	0.627698	-0.438812	3.832061
C	3.049930	2.868955	0.446371
H	2.015349	3.128111	0.697499
C	3.949961	3.976344	0.985573
H	3.929737	4.028822	2.079439
H	3.630875	4.947999	0.594320
H	4.994167	3.835292	0.685234
C	3.143114	2.795965	-1.078943
H	4.165762	2.550522	-1.390449
H	2.860609	3.748287	-1.540601
H	2.479992	2.023348	-1.483975
C	-3.106037	-0.284115	0.773869
C	-4.004914	0.084883	-0.243684
C	-5.127212	-0.717992	-0.467617
H	-5.826520	-0.436454	-1.253070
C	-5.362635	-1.858447	0.286395
H	-6.247592	-2.462520	0.105661
C	-4.444846	-2.235098	1.260822
H	-4.613722	-3.144780	1.832660
C	-3.301576	-1.474359	1.510862
C	-2.288512	-1.929081	2.544517
H	-1.329067	-1.468349	2.272716
C	-2.092512	-3.442773	2.547227
H	-2.975483	-3.971576	2.922594
H	-1.258953	-3.716755	3.203484
H	-1.873712	-3.830231	1.546071
C	-2.640112	-1.441654	3.951444
H	-2.605597	-0.351170	4.029657
H	-1.936579	-1.843289	4.688931
H	-3.646566	-1.769985	4.236817
C	0.740876	-0.814083	-1.474209

C	2.055876	-1.145917	-1.366647
H	2.724203	-0.356909	-1.008015
C	-0.330640	-1.680891	-2.009320
C	-0.572510	-1.774358	-3.390483
H	0.117719	-1.289500	-4.078463
C	-1.670791	-2.478358	-3.877922
H	-1.837314	-2.535423	-4.950768
C	-2.557520	-3.101491	-3.001890
H	-3.421121	-3.637795	-3.384306
C	-2.332002	-3.020873	-1.628662
H	-3.022566	-3.484980	-0.928161
C	-1.235564	-2.317138	-1.142618
H	-1.070177	-2.244799	-0.064633
C	2.770173	-2.390863	-1.634155
C	2.155970	-3.604503	-1.998448
H	1.079897	-3.650667	-2.124952
C	2.914166	-4.753525	-2.181031
H	2.420383	-5.681236	-2.457354
C	4.297618	-4.727441	-2.007478
H	4.882964	-5.631803	-2.149207
C	4.922863	-3.535740	-1.647189
H	5.999993	-3.504567	-1.506587
C	4.167099	-2.385885	-1.463273
H	4.647906	-1.455780	-1.165493
C	-3.766476	1.284688	-1.135441
H	-2.864320	1.799358	-0.781629
C	-4.924375	2.279692	-1.102103
H	-5.133609	2.633230	-0.087430
H	-4.701787	3.154042	-1.721823
H	-5.847401	1.831241	-1.487003
C	-3.509439	0.815866	-2.568871
H	-2.727576	0.048117	-2.608286
H	-4.410954	0.365927	-3.001603
H	-3.217957	1.654728	-3.212583
C	0.395144	4.895132	-3.298871
C	0.092605	3.541775	-3.202997
C	-0.115800	2.941730	-1.950012
C	-0.044164	3.751062	-0.805394
C	0.271835	5.101571	-0.905333
C	0.499434	5.682172	-2.151889
H	0.559255	5.338491	-4.277825
H	0.032358	2.926276	-4.098593
H	-0.248485	3.300548	0.167191
H	0.329866	5.705558	-0.002608
H	0.742682	6.737599	-2.230972
N	-0.360080	1.566716	-1.803550
H	-0.996423	1.232310	-2.521879
H	0.490005	0.541937	-1.810434

6. NMR Spectra

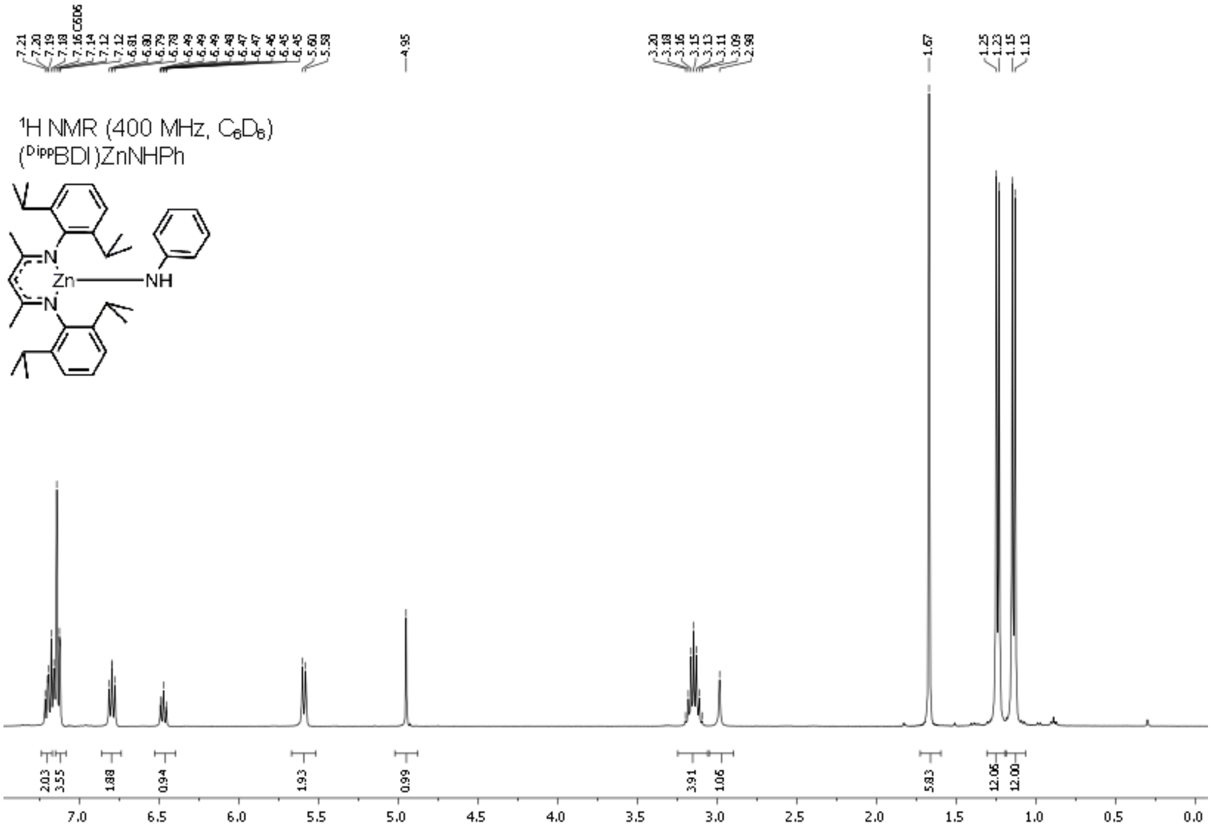

Figure S33. *^{1}H NMR spectrum of (^{Dipp}BDI)ZnNHPh **1** in C_6D_6.*

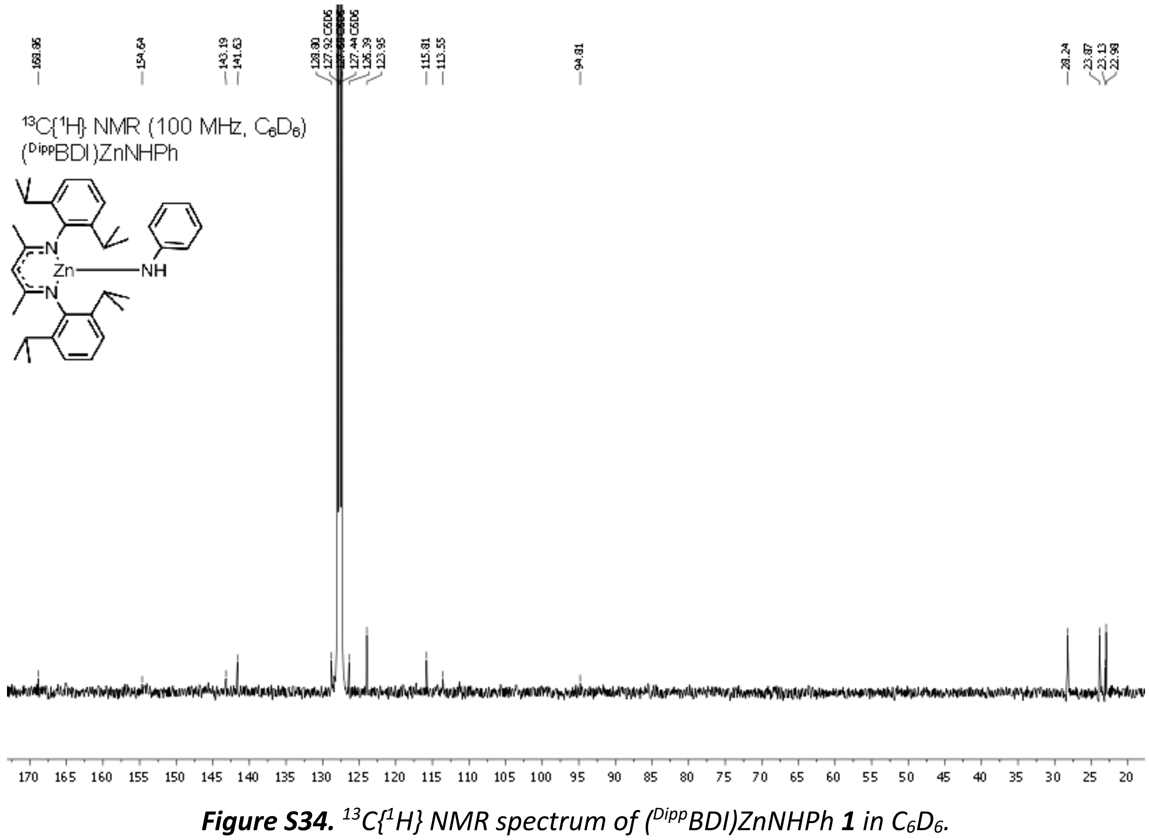

Figure S34. *^{13}C{^{1}H} NMR spectrum of (^{Dipp}BDI)ZnNHPh **1** in C_6D_6.*

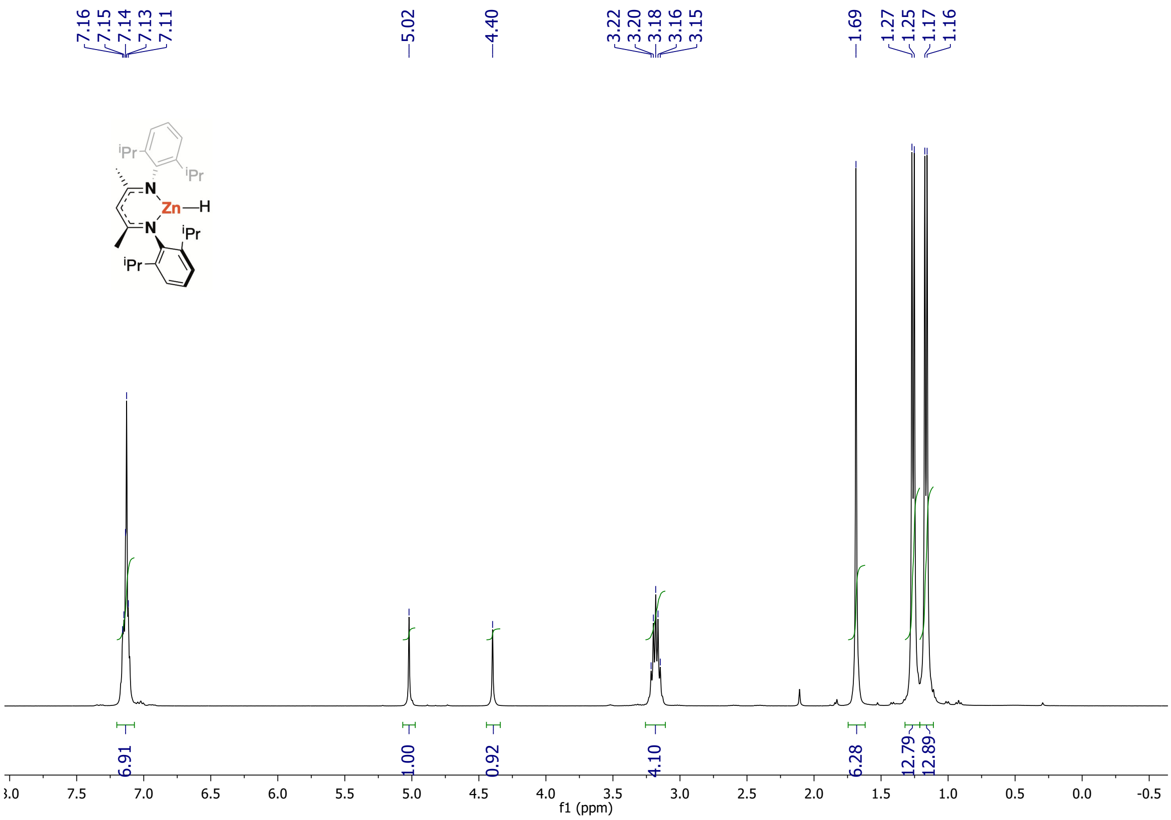

Figure S35. *^{1}H NMR spectrum of (^{Dipp}BDI)ZnH* ***2*** *in C_6D_6.*

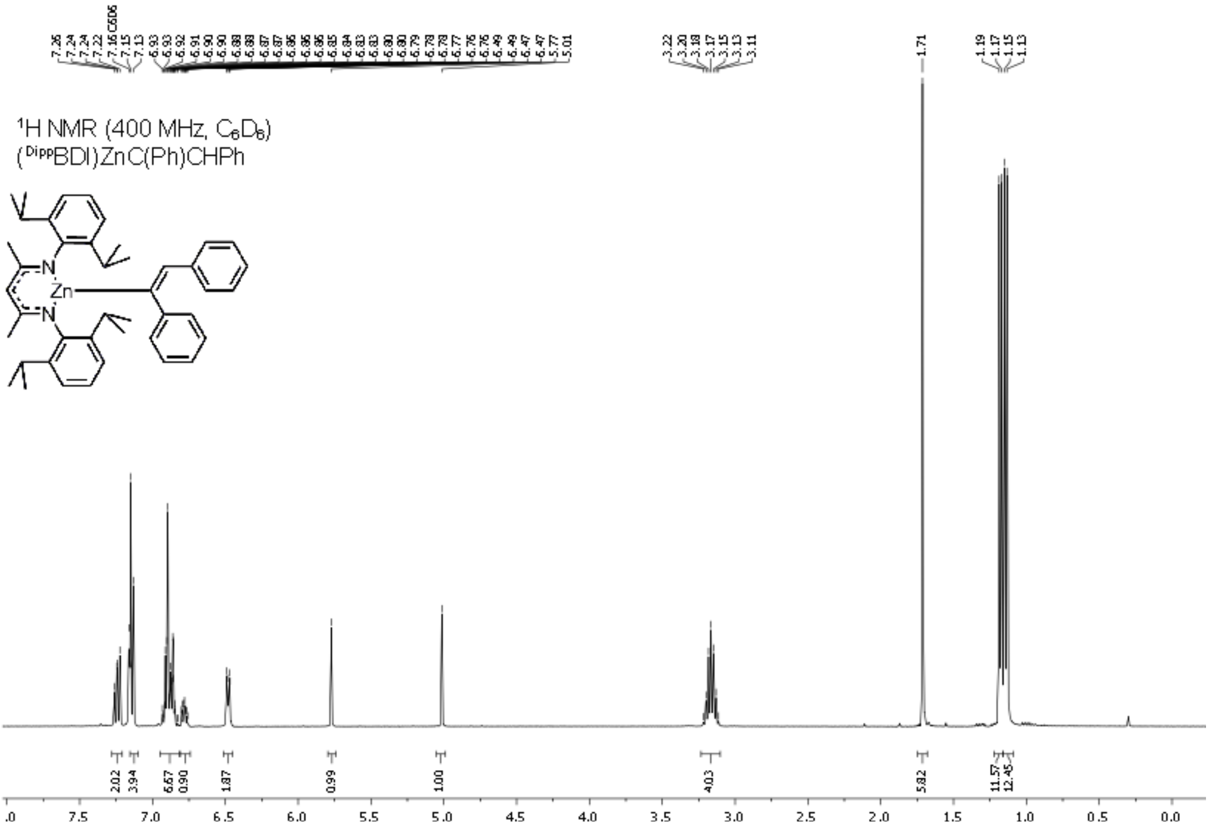

Figure S36. *^{1}H NMR spectrum of (^{Dipp}BDI)ZnC(Ph)CHPh* ***3a*** *in C_6D_6.*

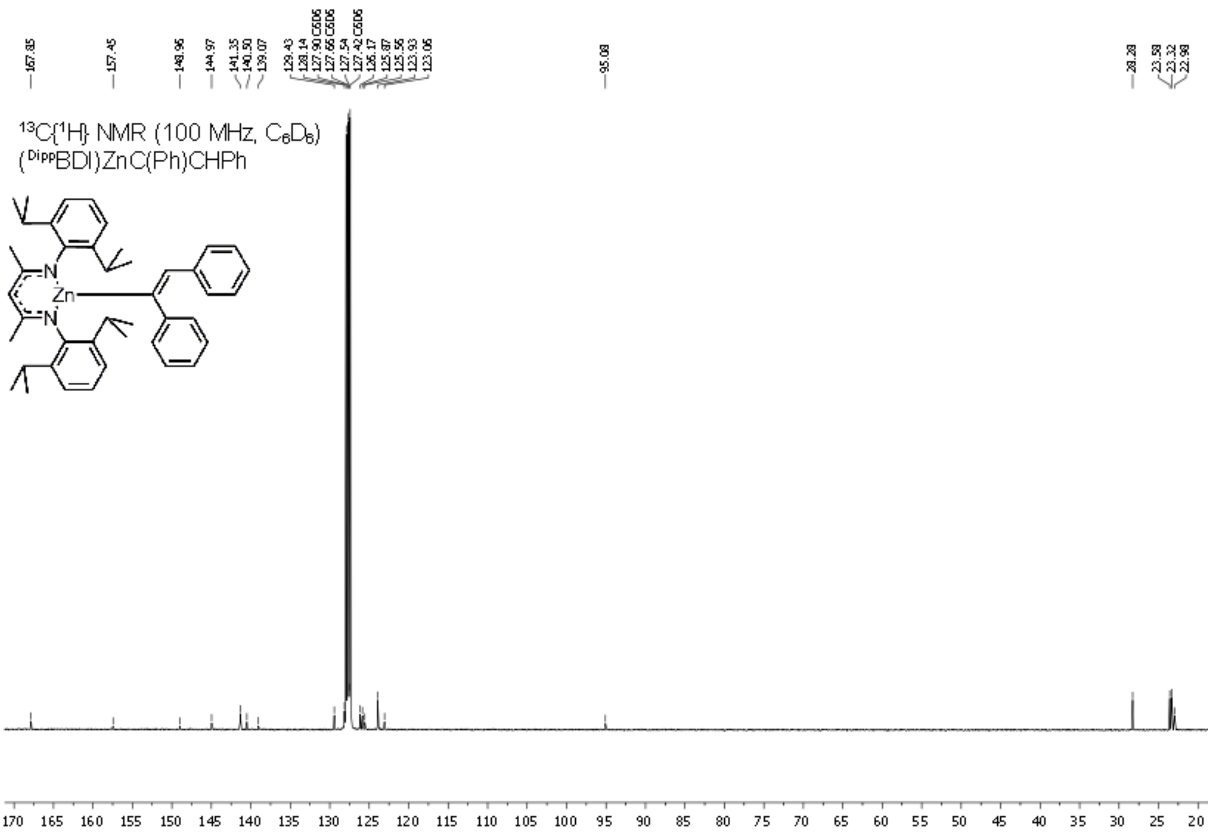

Figure S37. *$^{13}C\{^{1}H\}$ NMR spectrum of (^{Dipp}BDI)ZnC(Ph)CHPh **3a** in C_6D_6.*

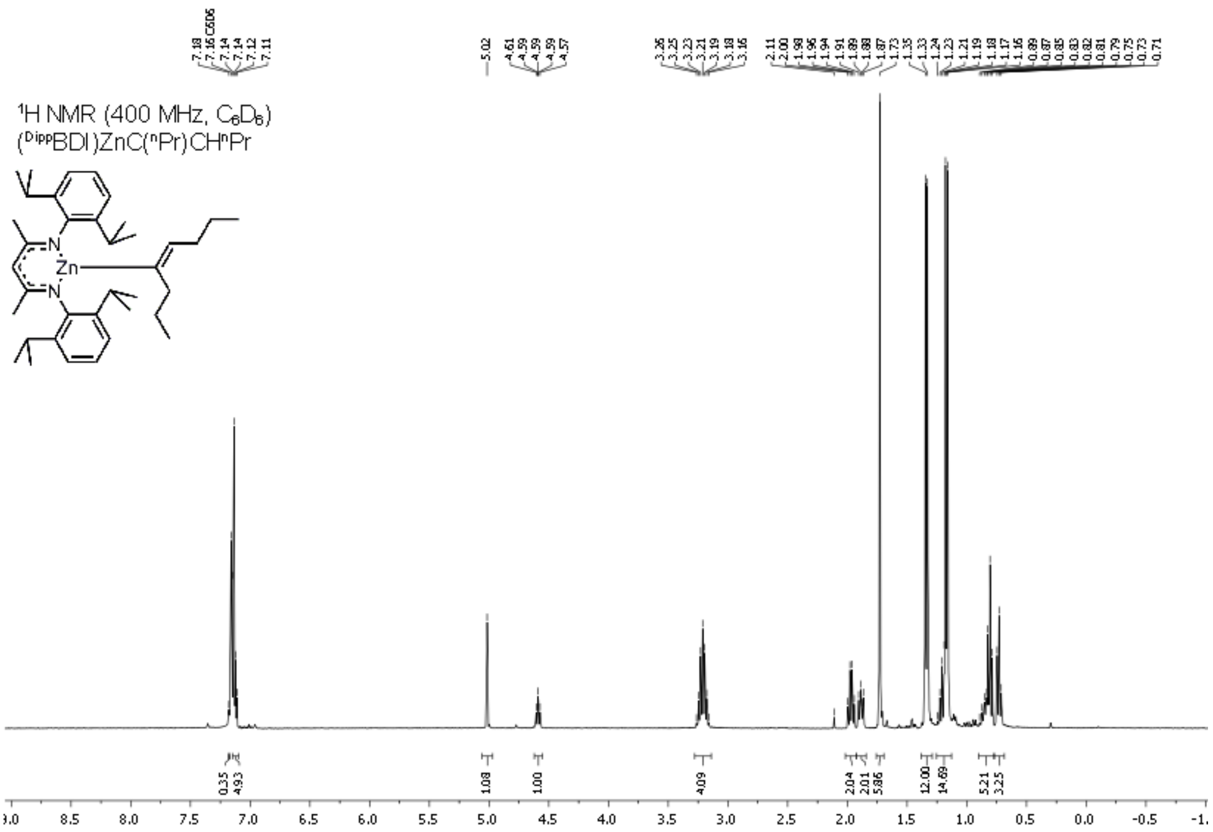

Figure S38. *^{1}H NMR spectrum of (^{Dipp}BDI)ZnC(^{n}Pr)CH^{n}Pr **3b** in C_6D_6.*

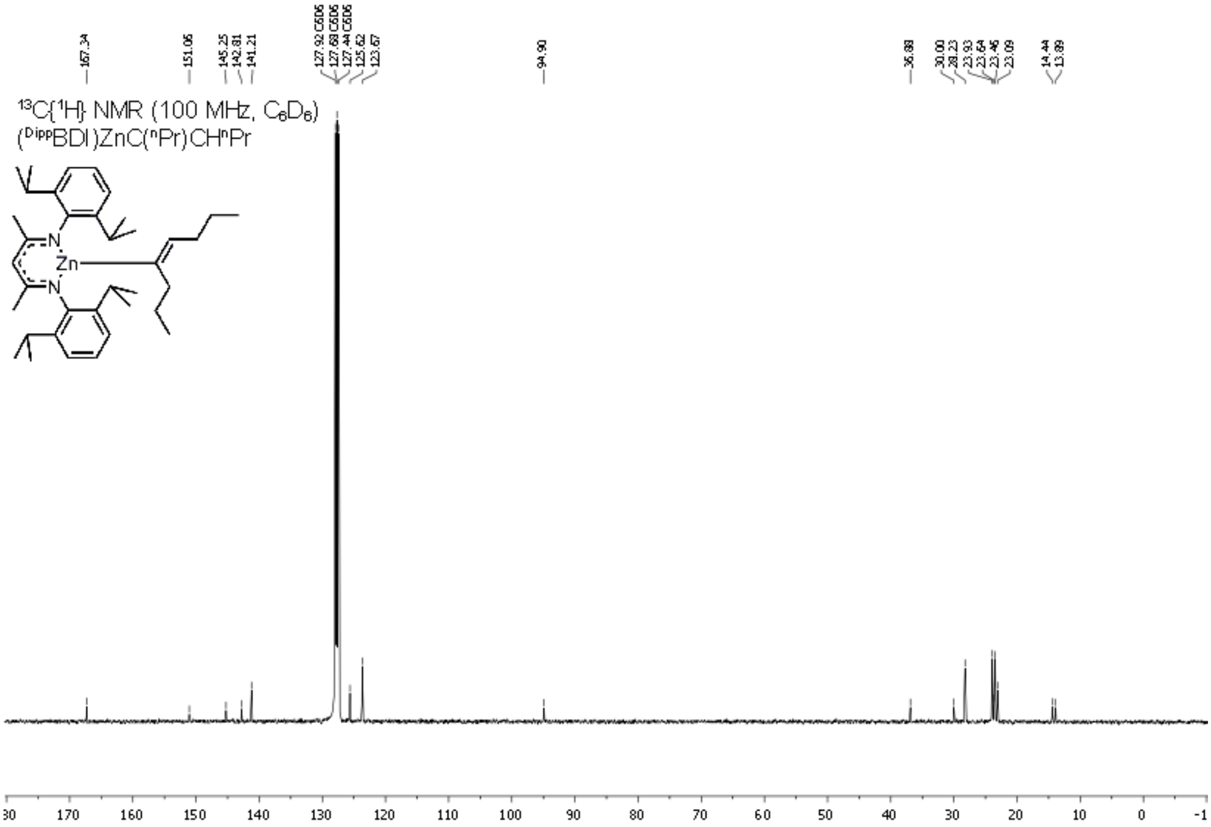

Figure S39. *$^{13}C\{^1H\}$ NMR spectrum of (^{Dipp}BDI)ZnC(^{n}Pr)CH^{n}Pr* ***3b*** *in C_6D_6.*

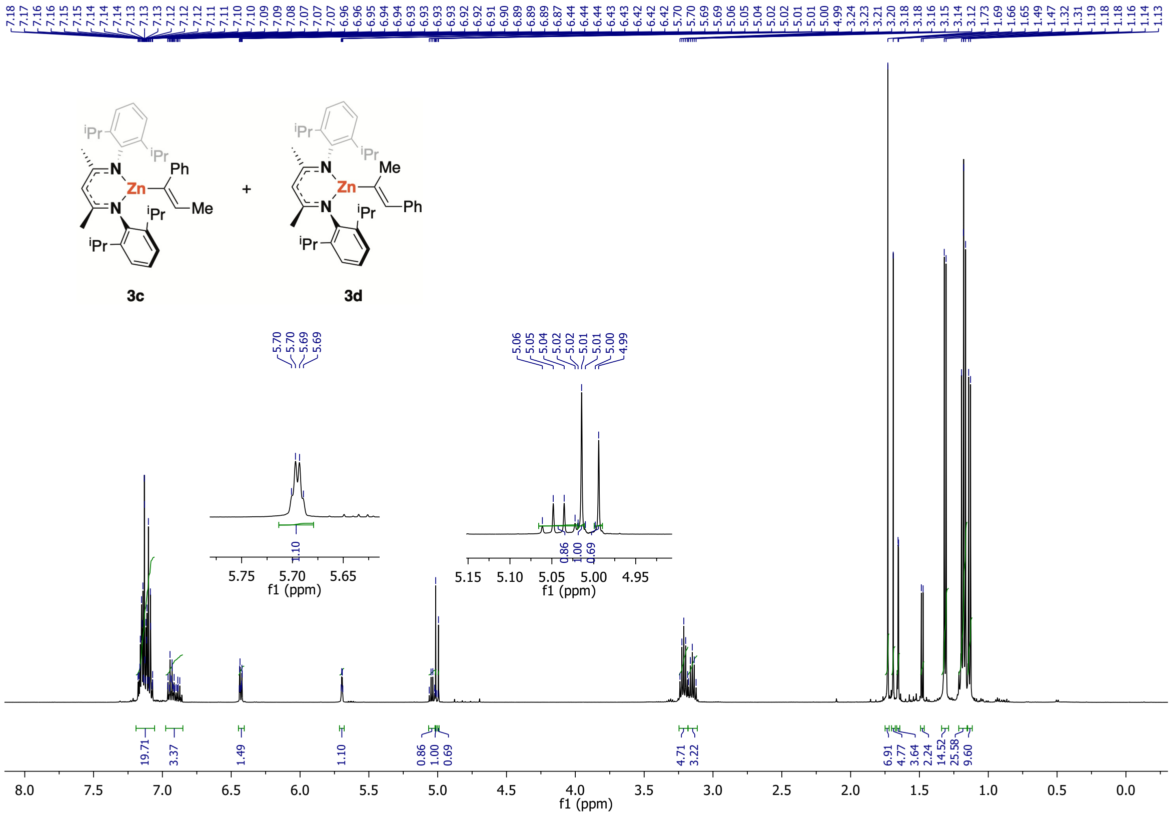

Figure S40. *^1H NMR spectrum of* ***3c/3d*** *in C_6D_6.*

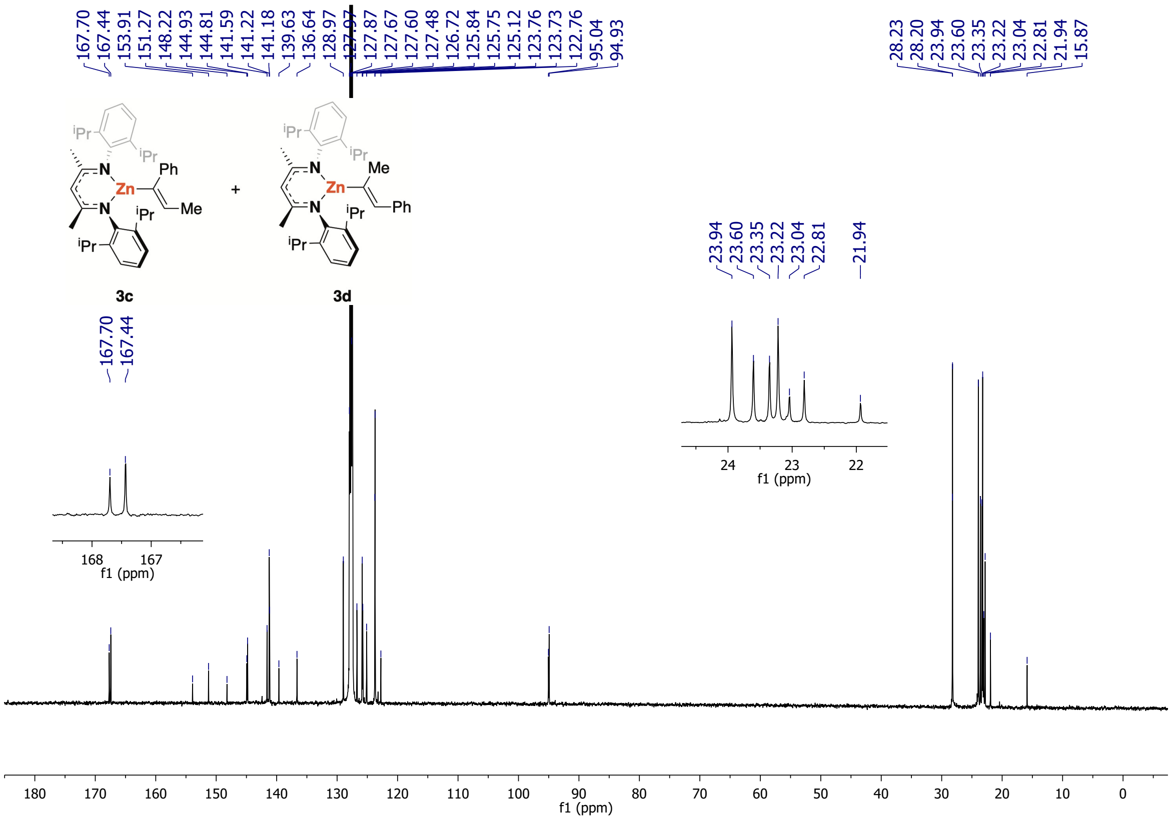

Figure S41. *$^{13}C\{^1H\}$ NMR spectrum of* ***3c/3d*** *in C_6D_6.*

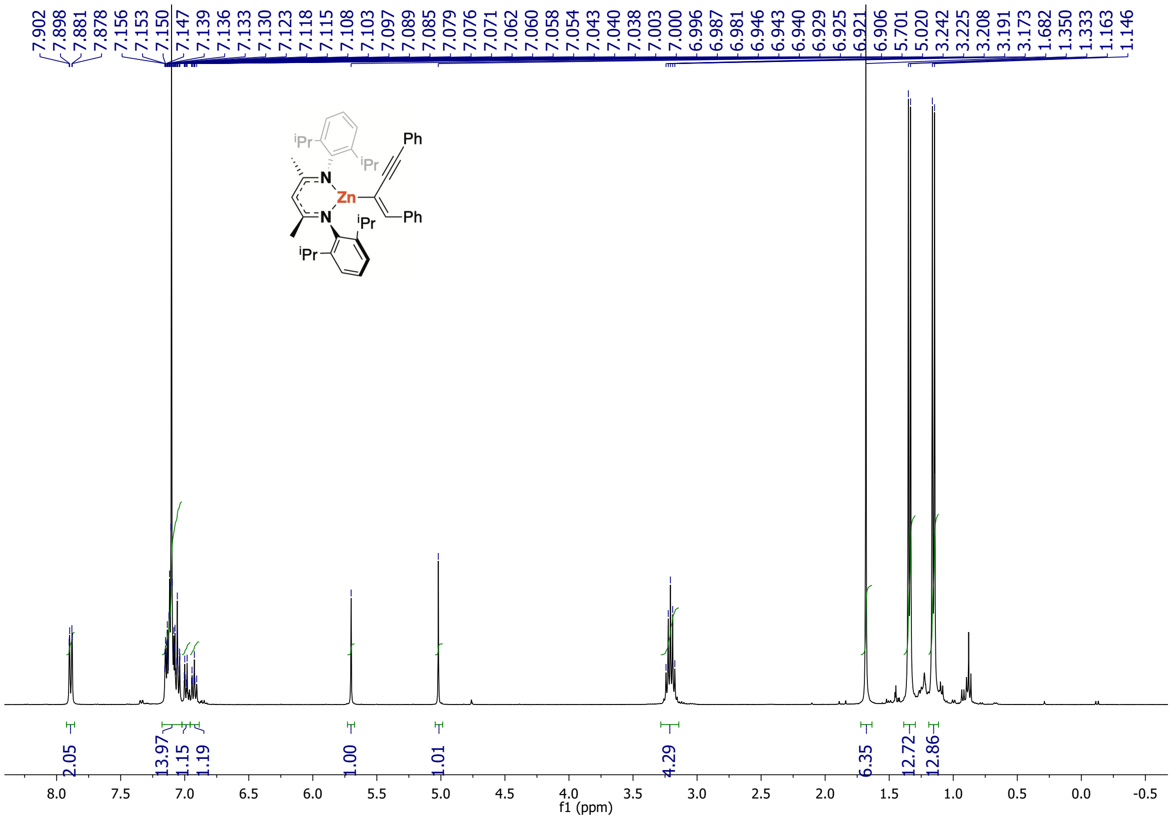

Figure S42. *^1H NMR spectrum of* ***3e*** *C_6D_6.*

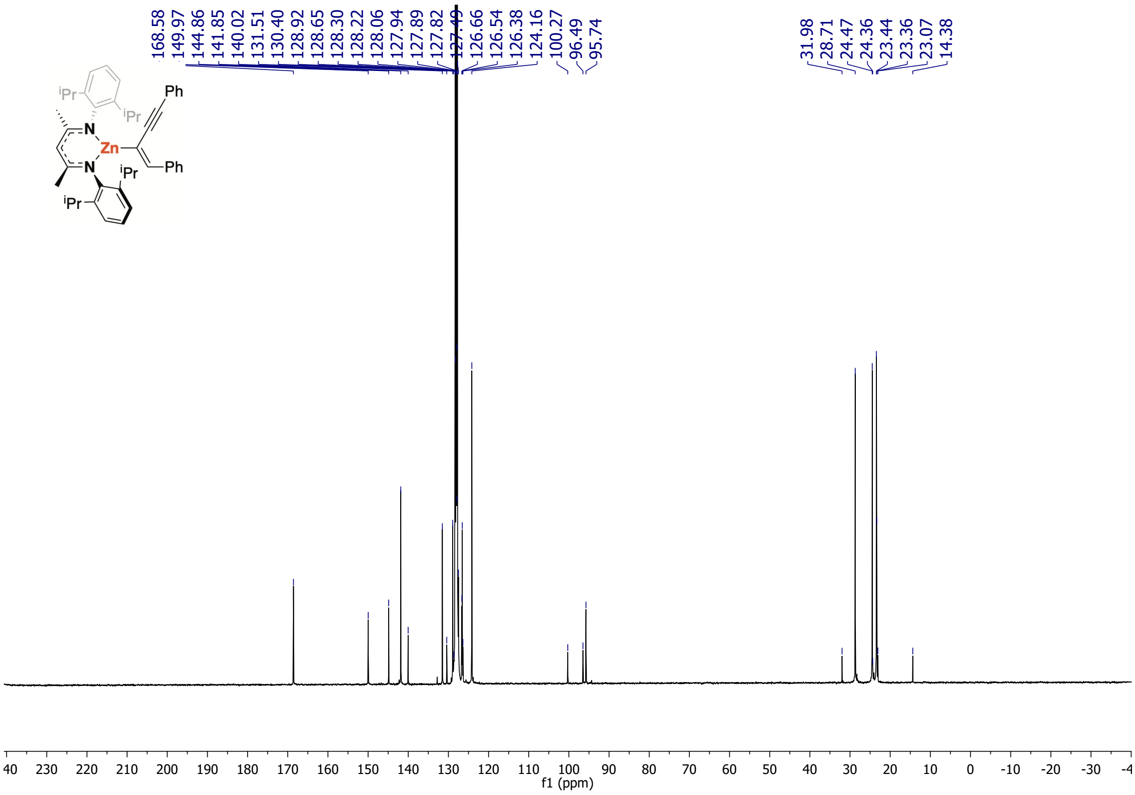

Figure S43. *$^{13}C\{^1H\}$ NMR spectrum of **3e** in C_6D_6.*

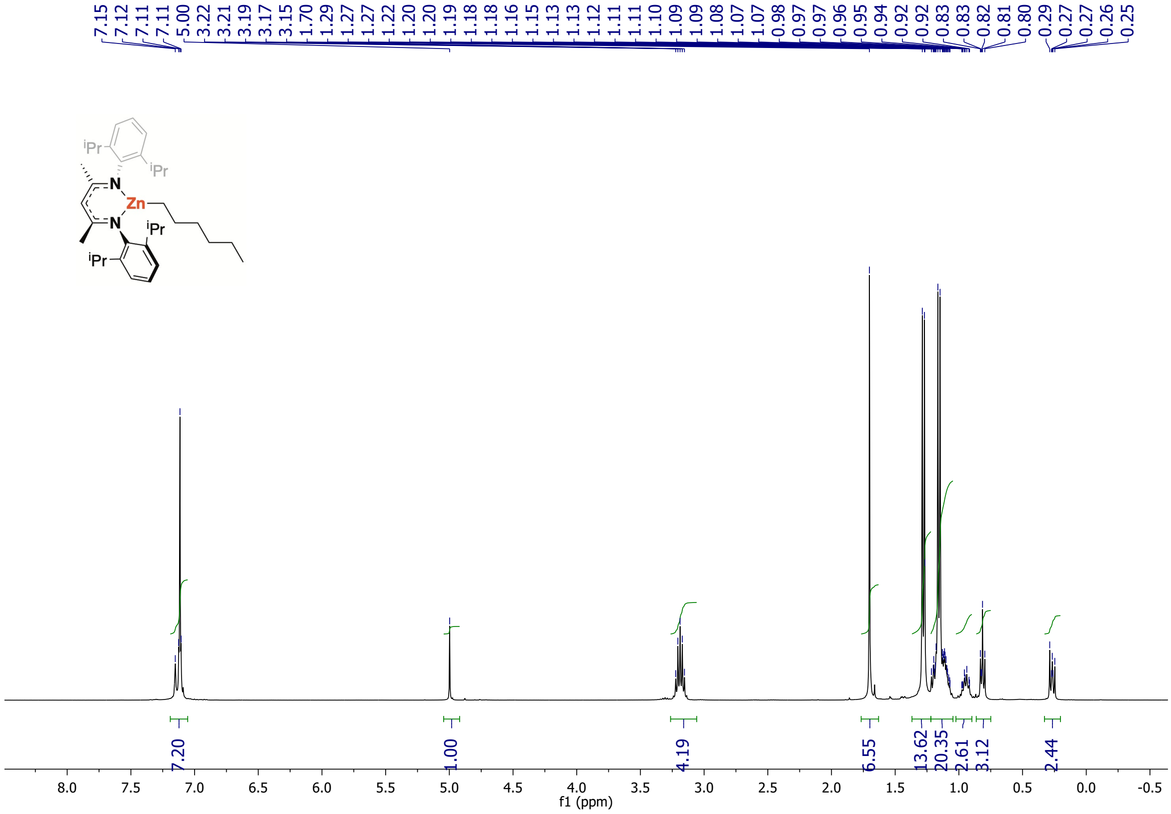

Figure S44. *^1H NMR spectrum of **3f** C_6D_6.*

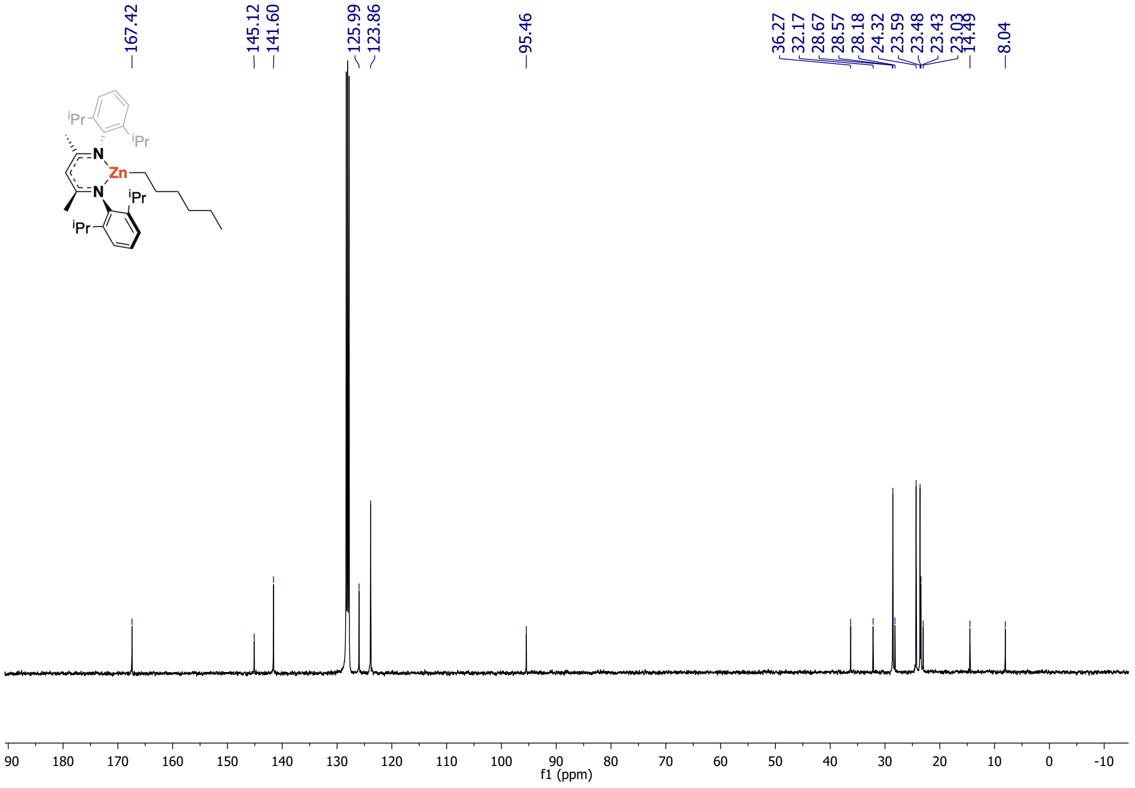

Figure S45. *$^{13}C\{^{1}H\}$ NMR spectrum of **3f** in C_6D_6.*

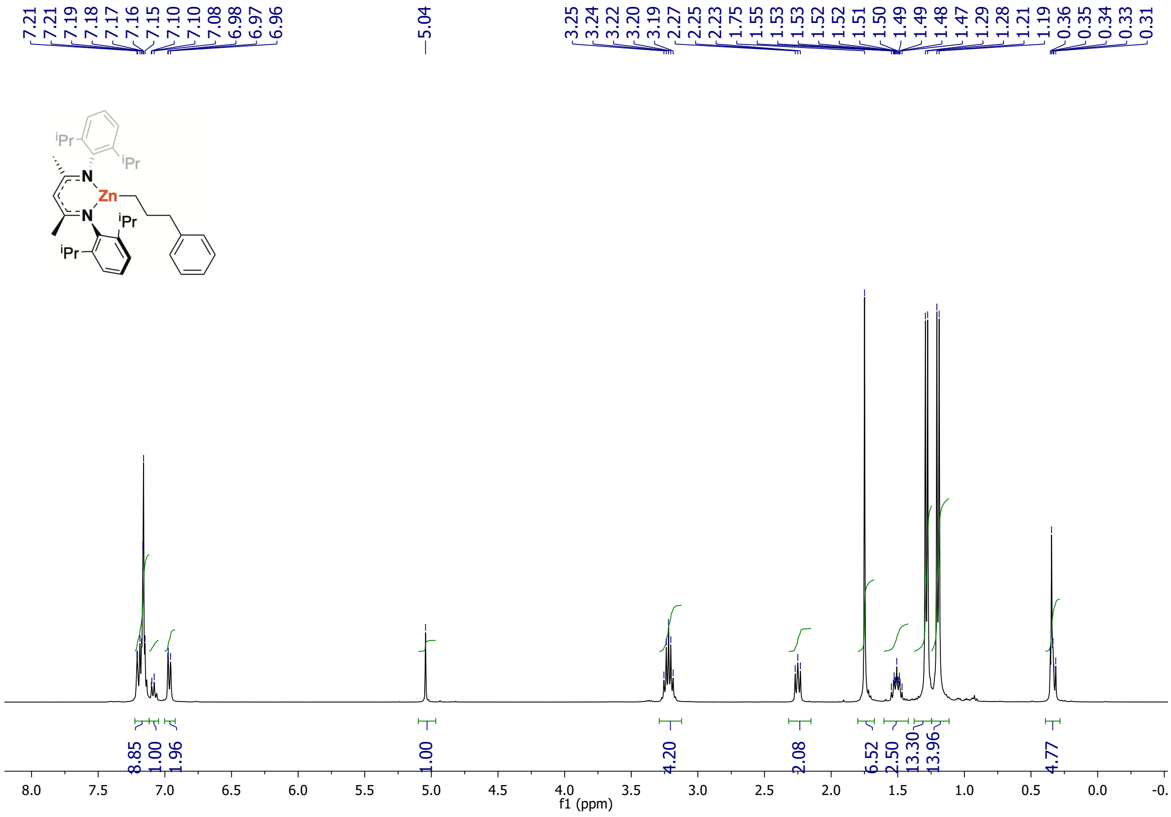

Figure S46. *^{1}H NMR spectrum of **3g** C_6D_6.*

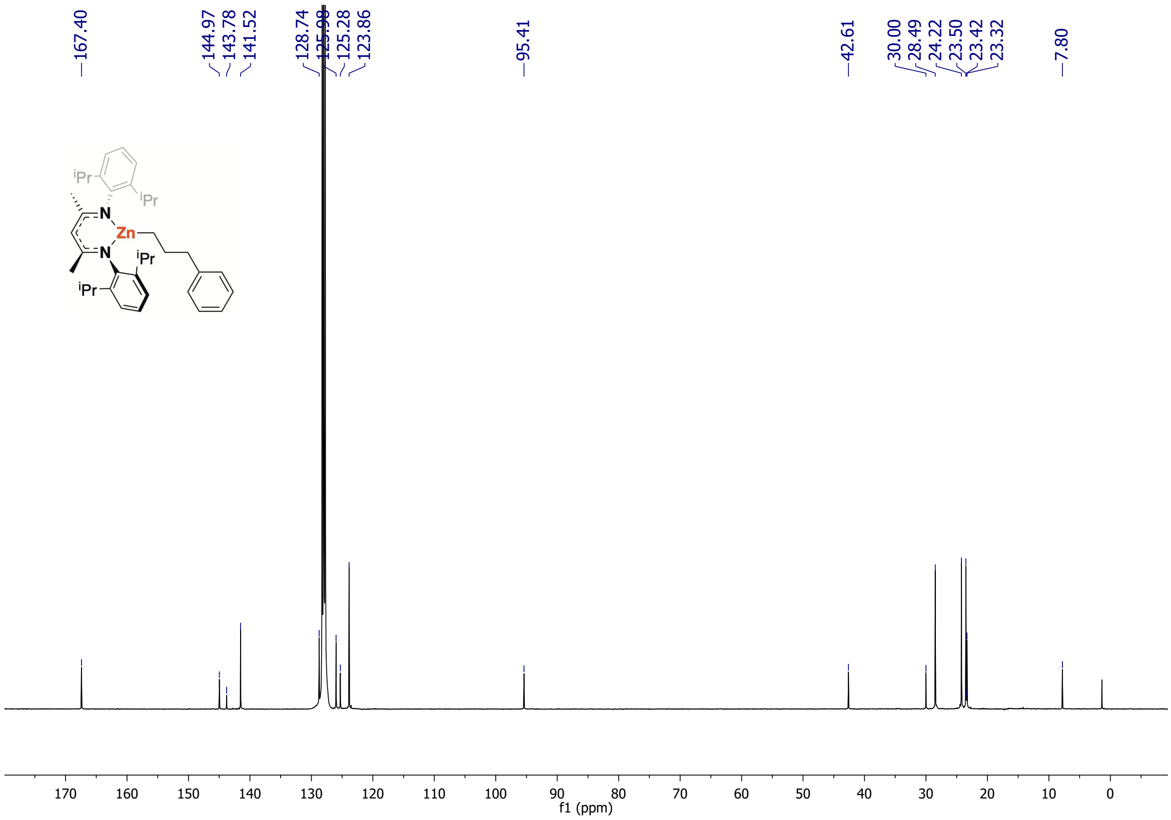

Figure S47. *$^{13}C\{^{1}H\}$ NMR spectrum of **3g** in C_6D_6.*

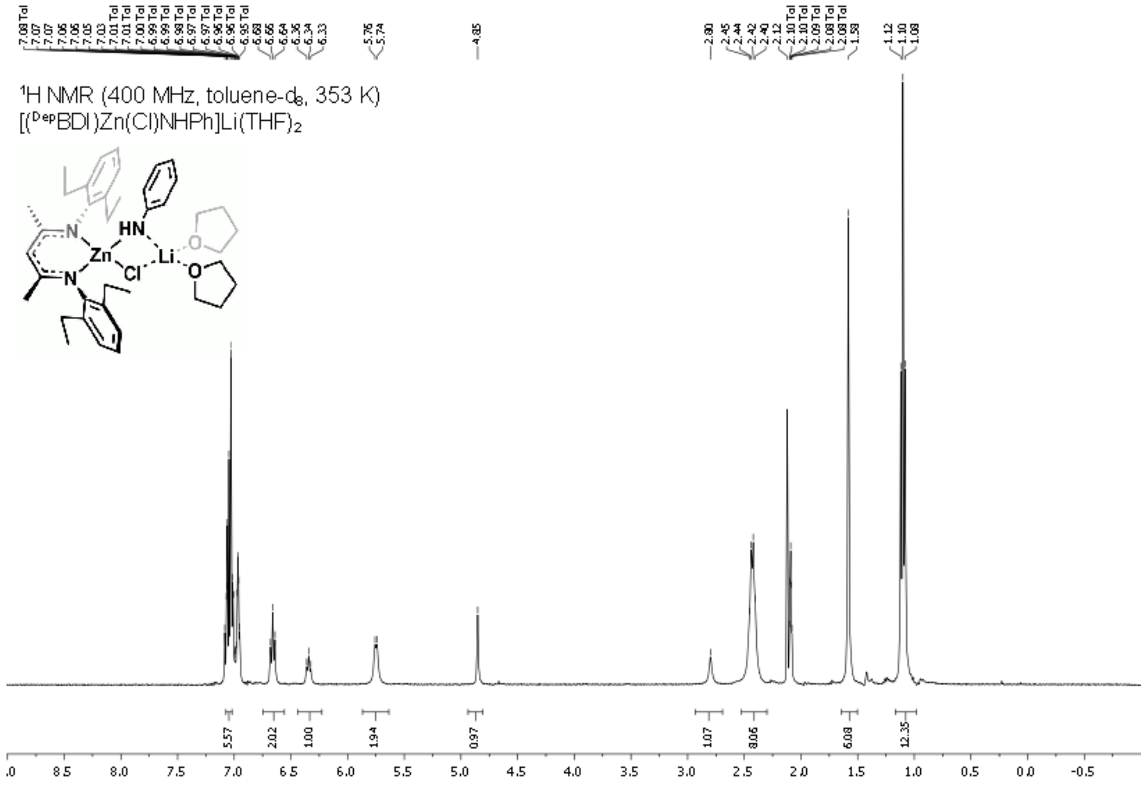

Figure S48. *^{1}H NMR spectrum of [(^{Dep}BDI)Zn(Cl)NHPh]Li **4** in d_8-toluene at 353 K.*

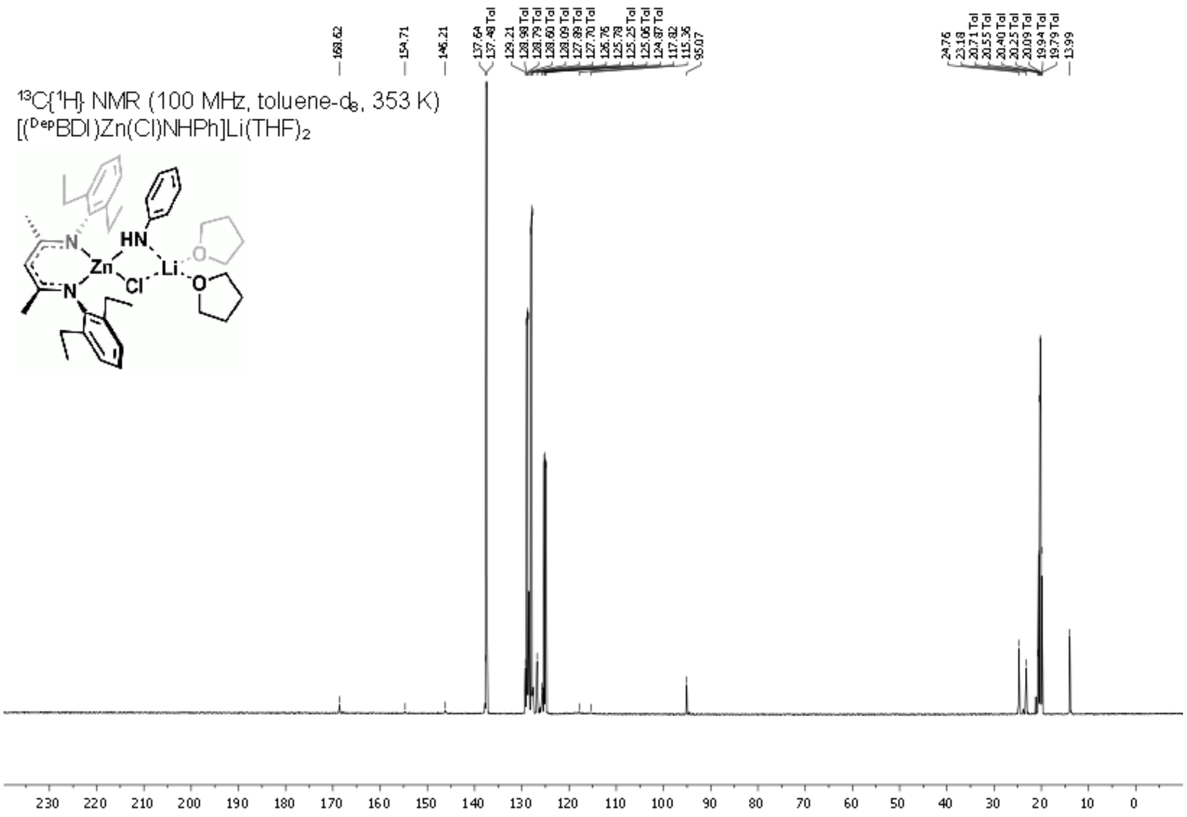

Figure S49. *$^{13}C\{^{1}H\}$ NMR spectrum of [(^{Dep}BDI)Zn(Cl)NHPh]Li* ***4*** *in d_8-toluene at 353 K.*

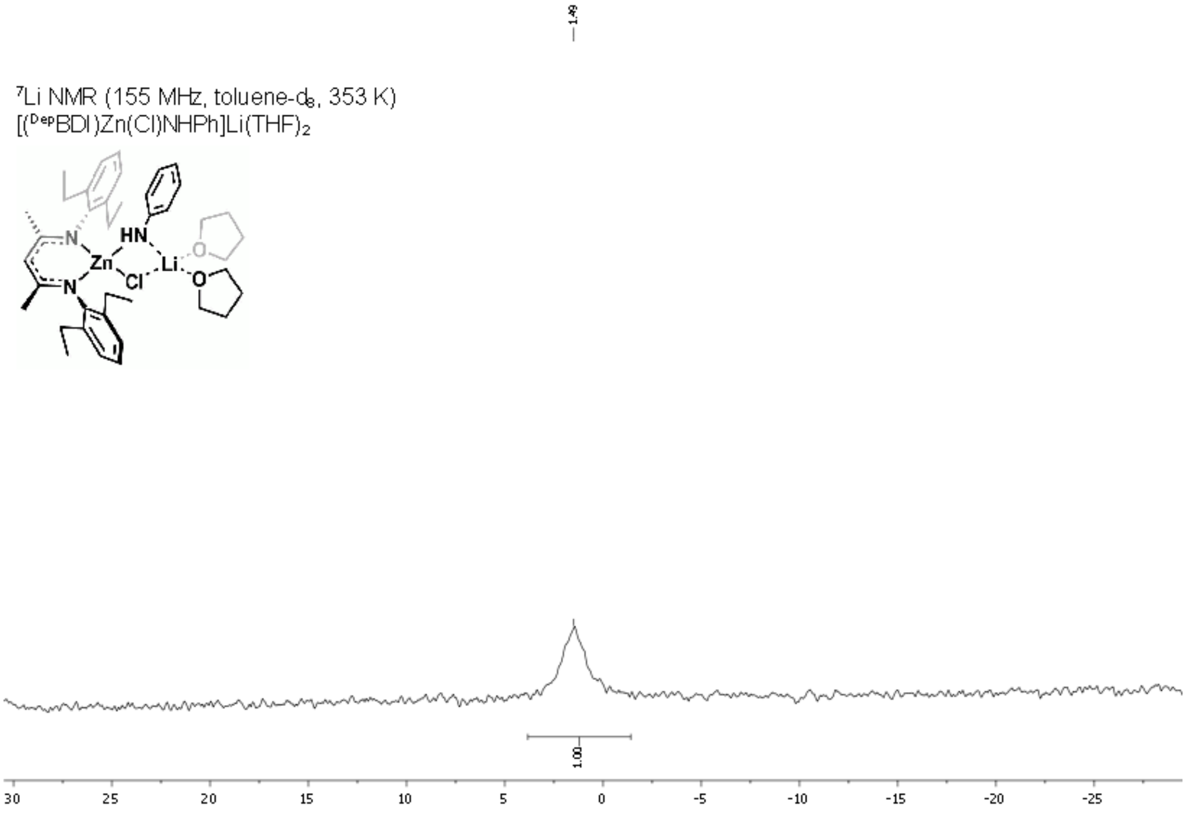

Figure S50. *^{7}Li NMR spectrum of [(^{Dep}BDI)Zn(Cl)NHPh]Li* ***4*** *in d_8-toluene at 353 K.*

7. References

(1) Sheldrick, G. M. SHELXTL v5.1. Bruker AXS: Madison, WI 1998.

(2) Sheldrick, G. M. Crystal Structure Refinement with SHELXL. *Acta Cryst. C* **2015**, *71* (1), 3–8.

(3) Feldman, J.; McLain, S. J.; Parthasarathy, A.; Marshall, W. J.; Calabrese, J. C.; Arthur, S. D. Electrophilic Metal Precursors and a β-Diimine Ligand for Nickel(II)- and Palladium(II)-Catalyzed Ethylene Polymerization. *Organometallics* **1997**, *16* (8), 1514–1516.

(4) Cheng, M.; Moore, D. R.; Reczek, J. J.; Chamberlain, B. M.; Lobkovsky, E. B.; Coates, G. W. Single-Site β-Diiminate Zinc Catalysts for the Alternating Copolymerization of CO_2 and Epoxides: Catalyst Synthesis and Unprecedented Polymerization Activity. *J. Am. Chem. Soc.* **2001**, *123* (36), 8738–8749.

(5) Spielmann, J.; Piesik, D.; Wittkamp, B.; Jansen, G.; Harder, S. Convenient Synthesis and Crystal Structure of a Monomeric Zinc Hydride Complex with a Three-Coordinate Metal Center. *Chem. Commun.* **2009**, *23*, 3455–3456.

(6) Mio, M. J.; Kopel, L. C.; Braun, J. B.; Gadzikwa, T. L.; Hull, K. L.; Brisbois, R. G.; Markworth, C. J.; Grieco, P. A. One-Pot Synthesis of Symmetrical and Unsymmetrical Bisarylethynes by a Modification of the Sonogashira Coupling Reaction. *Org. Lett.* **2002**, *4* (19), 3199–3202.

(7) Monfredini, A.; Santacroce, V.; Marchiò, L.; Maggi, R.; Bigi, F.; Maestri, G.; Malacria, M. Semi-Reduction of Internal Alkynes with Prototypical Subnanometric Metal Surfaces Bridging Homogeneous and Heterogeneous Catalysis with Trinuclear All-Metal Aromatics. *ACS Sus. Chem. Eng.* **2017**, 5, 8205−8212.

(8) Shi, M.; Xu, B. VO(acac)$_2$-Catalyzed Oxidative Coupling Reactions of Phosphonium Salts, *J. Org. Chem.* **2002**, *67*, 294–297.

(9) Zhou, H.; Moberg, C. Tunable Cross Coupling of Silanols: Selective Synthesis of Heavily Substituted Allenes and Butadienes, *J. Am. Chem. Soc.* **2012**, *134*, 15992–15999.

(10) Fu, S.; Chen, N.-Y.; Liu, X.; Shao, Z.; Luo, S.-P.; Liu, Q. Ligand-Controlled Cobalt-Catalyzed Transfer Hydrogenation of Alkynes: Stereodivergent Synthesis of Z - and E- Alkenes. *J. Am. Chem. Soc.* **2016**, *138*, 8588–8594.

(11) Shen, R.; Chen, T.; Zhao, Y.; Qiu, R.; Zhou, Y.; Yin, S.; Wang, X.; Goto, M.; Han, L.-B. Facile Regio- and Stereoselective Hydrometalation of Alkynes with a Combination of Carboxylic Acids and Group 10 Transition Metal Complexes: Selective Hydrogenation of Alkynes with

Formic Acid. *J. Am. Chem. Soc.* **2011**, *133*, 17037–17044.

(12) Li, J.; Hua, R.; Liu, T. Highly Chemo- and Stereoselective Palladium-Catalyzed Transfer Semihydrogenation of Internal Alkynes Affording Cis -Alkenes. *J. Org. Chem.* **2010**, *75*, 2966–2970.

(13) Dong, D.-J.; Li, H.-H.; Tian, S.-K. A Highly Tunable Stereoselective Olefination of Semistabilized Triphenylphosphonium Ylides with N -Sulfonyl Imines. *J. Am. Chem. Soc.* **2010**, *132*, 5018–5020.

(14) Chernichenko, K.; Madarász, A.; Pápai, I.; Nieger, M.; Leskelä, M.; Repo, T. A Frustrated-Lewis-pair Approach to Catalytic Reduction of Alkynes to cis-Alkenes. *Nature Chem.* **2013**, *5*, 718-723.

(15) Gaussian 09, Revision D.01, M. J. Frisch, G. W. Trucks, H. B. Schlegel, G. E. Scuseria, M. A. Robb, J. R. Cheeseman, G. Scalmani, V. Barone, B. Mennucci, G. A. Petersson, et al. Gaussian, Inc. Wallingford CT, **2009**.

(16) Zhao, Y.; Truhlar, D. G. A new local density functional for main-group thermochemistry, transition metal bonding, thermochemical kinetics, and noncovalent interactions. *J. Chem. Phys.* **2006**, *125*, 194101.

(17) Hehre, W. J.; Ditchfield, R.; Pople, J. A. Self—Consistent Molecular Orbital Methods. XII. Further Extensions of Gaussian—Type Basis Sets for Use in Molecular Orbital Studies of Organic Molecules. *J. Chem. Phys.* **1972**, *56*, 2257–2261.

(18) Hariharan, P. C.; Pople, J. A. The influence of polarization functions on molecular orbital hydrogenation energies. *Theor. Chim. Acta.* **1973**, *28*, 213–222.

(19) Clark, T.; Chandrasekhar, J.; Spitznagel, G. W.; Schleyer, P. V. R. Efficient diffuse function augmented basis sets for anion calculations. III. The 3-21+G basis set for first-row elements, Li–F. *J. Comput. Chem*. **1983**, *4*, 294–301.

(20) Fukui, K. The path of chemical reactions - the IRC approach. *Acc. Chem. Res.*, **1981**, *14*, 363–368.

(21) Hratchian, H. P.; Schlegel, H. B. Chapter 10 - Finding minima, transition states, and following reaction pathways on ab initio potential energy surfaces. *Theory and Applications of Computational Chemistry – The First Forty Years*, 1; Elsevier: London, 2005, 195–249.

(22) Tomasi, J.; Mennucci, B.; Cammi, R. Quantum Mechanical Continuum Solvation Models. *Chem. Rev.* **2005**, *105*, 2999–3094.

(23) Grimme, S.; Antony, J.; Ehrlich, S.; Krieg, H. A consistent and accurate ab initio parametrization of density functional dispersion correction (DFT-D) for the 94 elements H-Pu. *J. Chem. Phys.* **2010**, *132*, 154104.

(24) Head-Gordon, M.; Chai, J. D. Long-range corrected hybrid density functionals with damped atom–atom dispersion corrections. *Phys. Chem. Chem. Phys.* **2008**, *10*, 6615–6620.

(25) Grimme, S. Semiempirical GGA-type density functional constructed with a long-range dispersion correction. *J. Comp. Chem.* **2006**, *27*, 1787-1799.

(26) GaussView, Version 5, Dennington, R.; Keith, T.; Millam, J. Semichem, Inc., Shawnee Mission, KS, **2009**.

(27) Glendening, E. D.; Landis, C. R.; Weinhold, F. NBO 6.0: Natural Bond Orbital Analysis Program, *J. Comput. Chem.* **2013**, *34*, 1429-1437.
